# Carfilzomib modulates tumor microenvironment to potentiate immune checkpoint therapy for cancer

**DOI:** 10.15252/emmm.202114502

**Published:** 2021-12-13

**Authors:** Qian Zhou, Jinxia Liang, Tong Yang, Jin Liu, Bo Li, Yingchang Li, Zhenzhen Fan, Weida Wang, Wensheng Chen, Sujing Yuan, Meng Xu, Qigui Xu, Zhidong Luan, Zhongjun Xia, Penghui Zhou, Yadong Huang, Liang Chen

**Affiliations:** ^1^ Key Laboratory of Functional Protein Research of Guangdong Higher Education Institutes and MOE Key Laboratory of Tumor Molecular Biology Institute of Life and Health Engineering College of Life Science and Technology Jinan University Guangzhou China; ^2^ MOE Key Laboratory of Glucolipid Metabolic Diseases Guangdong Metabolic Diseases Research Center of Integrated Chinese and Western Medicine College of Chinese Medicine Research Guangdong Pharmaceutical University Guangzhou China; ^3^ State Key Laboratory of Oncology in Southern China Collaborative Innovation Center for Cancer Medicine Sun Yat‐sen University Cancer Center Guangzhou China; ^4^ Department of Oncology The First Affiliated Hospital Jinan University Guangzhou China; ^5^ Translational medicine laboratory People’s Hospital of Yangjiang City Guangdong China; ^6^ Guangdong Province Key Laboratory of Bioengineering Medicine Jinan University Guangzhou China

**Keywords:** immunotherapy, M1 macrophage, M2 macrophage, tumor microenvironment, tumor‐associated macrophage, Cancer, Immunology

## Abstract

Impressive clinical benefit is seen in clinic with PD‐1 inhibitors on portion of cancer patients. Yet, there remains an urgent need to develop effective synergizers to expand their clinical application. Tumor‐associated macrophage (TAM), a type of M2‐polarized macrophage, eliminates or suppresses T‐cell‐mediated anti‐tumor responses. Transforming TAMs into M1 macrophages is an attractive strategy of anti‐tumor therapy. Here, we conducted a high‐throughput screening and found that Carfilzomib potently drove M2 macrophages to express M1 cytokines, phagocytose tumor cells, and present antigens to T cells. Mechanistically, Carfilzomib elicited unfolded protein response (UPR), activated IRE1α to recruit TRAF2, and activated NF‐κB to transcribe genes encoding M1 markers in M2 macrophages. *In vivo*, Carfilzomib effectively rewired tumor microenvironment through reprogramming TAMs into M1‐like macrophages and shrank autochthonous lung cancers in transgenic mouse model. More importantly, Carfilzomib synergized with PD‐1 antibody to almost completely regress autochthonous lung cancers. Given the safety profiles of Carfilzomib in clinic, our work suggested a potentially immediate application of combinational treatment with Carfilzomib and PD‐1 inhibitors for patients with solid tumors.

## Graphical abstract

Tumor‐associated macrophages (TAMs) are highly immunosuppressive. A high‐throughput drug screening was performed to identify FDA‐approved drugs that can reprogram TAMs into immunostimulatory M1 macrophages.

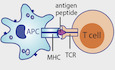

**The paper explained**

**Problem**

Tumor‐associated macrophages (TAM) are highly immunosuppressive. It is of high therapeutic value to target TAM with small molecular drugs capable of reprogramming TAM into immunostimulatory M1 cells.

**Results**

We conducted a high‐throughput screening for this purpose by checking the ability of a chemical to activate luciferase activity in M2‐polarized macrophages derived from bone marrow cells of IL1B‐luciferase transgenic mice. Carfilzomib was identified through this screening and was validated for reprogramming TAM into immunostimulatory M1 cells *in vitro* and *in vivo*. We also elucidated the underlying mechanism: Carfilzomib induced ER stress and activated IRE1a to activate NF‐κB through TRAF2, and therefore the expression of M1‐related genes. Carfilzomib was found to shrink tumors by itself or in combination with PD‐1 inhibitors.

**Impact**

A combination of Carfilzomib and PD‐1 inhibitors is worthy of further clinical trial to treat patients with solid tumors.

## Introduction

Antibodies against immunocheckpoint (ICP) prevent interaction between ICPs and their ligands and thereby restore the activity of cytotoxic T cells (CTLs) to lyse target cells expressing ICP ligands. Impressive clinical benefit was seen in oncological clinic with ICP inhibitors, typified by antibodies against PD‐1 or CTLA‐4 (Robert, 2020). Unfortunately, a significant portion of patients failed to respond to this type of treatment. EGFR mutation‐positive lung cancer patients, for example, were found to not only be unresponsive to PD‐1/PD‐L1 inhibition (Calles *et al*, 2020) but even to hyper‐progress after immunotherapy (Kato *et al*, 2017). Hence, there remains an urgent need to develop effective drugs to expand the clinical application of ICP inhibitors.

Most tissue macrophages, with a few exceptions, arise from yolk sac progenitors (Kierdorf *et al*, 2015; Munro & Hughes, 2017; Wu & Hirschi, 2020). Instead, pathogen‐fighting macrophages are likely derived from circulating bone marrow (BM) monocytes (Ginhoux & Jung, 2014). Studies have revealed the presence of both resident yolk sac‐derived and recruited BM‐derived macrophages (BMDM) in the tumor microenvironment. They execute distinct function in tumor immunology and respond differently to anti‐macrophage therapies like inhibitors against growth factor colony stimulating factor‐1 (CSF1; Kielbassa *et al*, 2019).

Macrophages derived from monocyte precursors undergo specific differentiation depending on the local tissue environment (Orecchioni *et al*, 2019). LPS, IFNγ, and granulocyte‐macrophage colony‐stimulating factor (GM‐CSF) polarize macrophages toward the M1 phenotype, which secrete cytokines such as IL‐1β, IL‐6, tumor necrosis factor (TNF), IL‐12, IL‐18, and IL‐23. M1 macrophages produce reactive nitrogen and oxygen intermediates and promote Th1 responses and strong microbicidal activity. Phenotypically, M1 macrophages express high levels of major histocompatibility complex class II (MHC II), CD68, and co‐stimulatory molecules, CD80 and CD86. In contrast, M2 macrophage activation is induced by IL‐4, IL‐13, IL‐10, and TGF‐β. These M2 cells, thereby, secrete high amounts of IL‐10 and low levels of IL‐12 (Orecchioni *et al*, 2019). Subsequent studies revealed that M2 macrophages can be further categorized into M2a, M2b, M2c, and M2d (Mantovani *et al*, 2004).

Immune system surveys organism for tumor development. Early phase of the surveillance features engagement of innate immunocytes like macrophages and acquired immune system like CD8^+^ T cells. Despite accumulating data suggesting better prognosis for patients with tumors infiltrated with CD8^+^ T cell, established tumors tolerate the attacks from immune system, suggesting that tumor microenvironment (TME) is immunosuppressive. Large amounts of macrophages are frequently detected in tumor foci (Pathria *et al*, 2019). It is now clear that rather than being tumoricidal, Tumor‐associated macrophages (TAMs) adopt a protumoral phenotype *in vivo* (Redente *et al*, 2010).

Tumor‐educated macrophages promote angiogenesis, cancer cell invasion, and intravasation in the primary site, as well as extravasation and persistent growth in the secondary site (Qian & Pollard, 2010; Kitamura *et al*, 2015). Tumor‐associated macrophages protect cancer cells through eliminating the anti‐tumor T cells by overexpressing PD‐L1, PD‐L2, CD80, and Siglec‐15 (Noy & Pollard, 2014; Mantovani *et al*, 2017; Wang *et al*, 2019). Tumor‐associated macrophages can also directly suppress T‐cell responses by either secreting cytokines or transforming tumor microenvironment metabolically (Curiel *et al*, 2004; Denning *et al*, 2007). Therefore, eliminating TAMs has been an attractive field. Antibodies and kinase inhibitors against CSF1R have been developed for this purpose (Ries *et al*, 2014). Unfortunately, clinical success of these efforts has so far been limited. Another strategy is to transform TAM into M1 macrophages. Targeting PI3Kγ and class IIa HDAC has been shown to transform TAM into M1 (Kaneda *et al*, 2016; Guerriero *et al*, 2017). A systemic identification of small molecules capable of transforming TAMs into M1 macrophages would lead to identification of not only compounds but also protein targets suitable for potential drug development.

Carfilzomib, a tetrapeptide epoxyketone, irreversibly inhibits the proteasome in its ChT‐L site and thus disrupts the cellular protein homeostasis (Kuhn *et al*, 2007). Myeloma cells, featuring production and secretion of large amounts of proteins, are especially sensitive to Carfilzomib (Muchtar *et al*, 2016). American food and drug administration (FDA) approved Carfilzomib for treating patients with relapsed/refractory multiple myeloma in 2012.

In this study, we conducted a high‐throughput screening of chemicals, aiming to repurpose FDA‐approved drugs for reprograming M2 macrophages into M1‐like population. We found that Carfilzomib, and other proteasome inhibitors to a lesser degree, were capable of effectively inducing M1‐like properties from M2 macrophages. These Carfilzomib‐induced M1‐like macrophages (Ci‐M1) secreted IL‐1β, upregulated CD80 and CD86, and exhibited enhanced ability to present antigens to T cells. Mechanistically, Carfilzomib treatment elicited ER stress (or unfolded protein response UPR), activated IRE1a to recruit TRAF2, and activated NF‐κB pathway for transcribing genes encoding M1 cytokines. Carfilzomib treatment increased infiltration of anti‐tumor inflammatory macrophages and CD8^+^ T cells in tumor loci and partially shrank mutant EGFR‐driven lung cancers. More importantly, Carfilzomib synergizes with PD‐1 antibody to treat this type of lung cancer, resulting in nearly complete tumor regression. Our work revealed a novel mechanism underlying anti‐tumor effect of Carfilzomib and may expand the usage of ICP inhibitors in cancer clinic.

## Results

### High‐throughput Screening of FDA drugs capable of inducing IL‐1β expression in M2 macrophages

**Figure 1:**
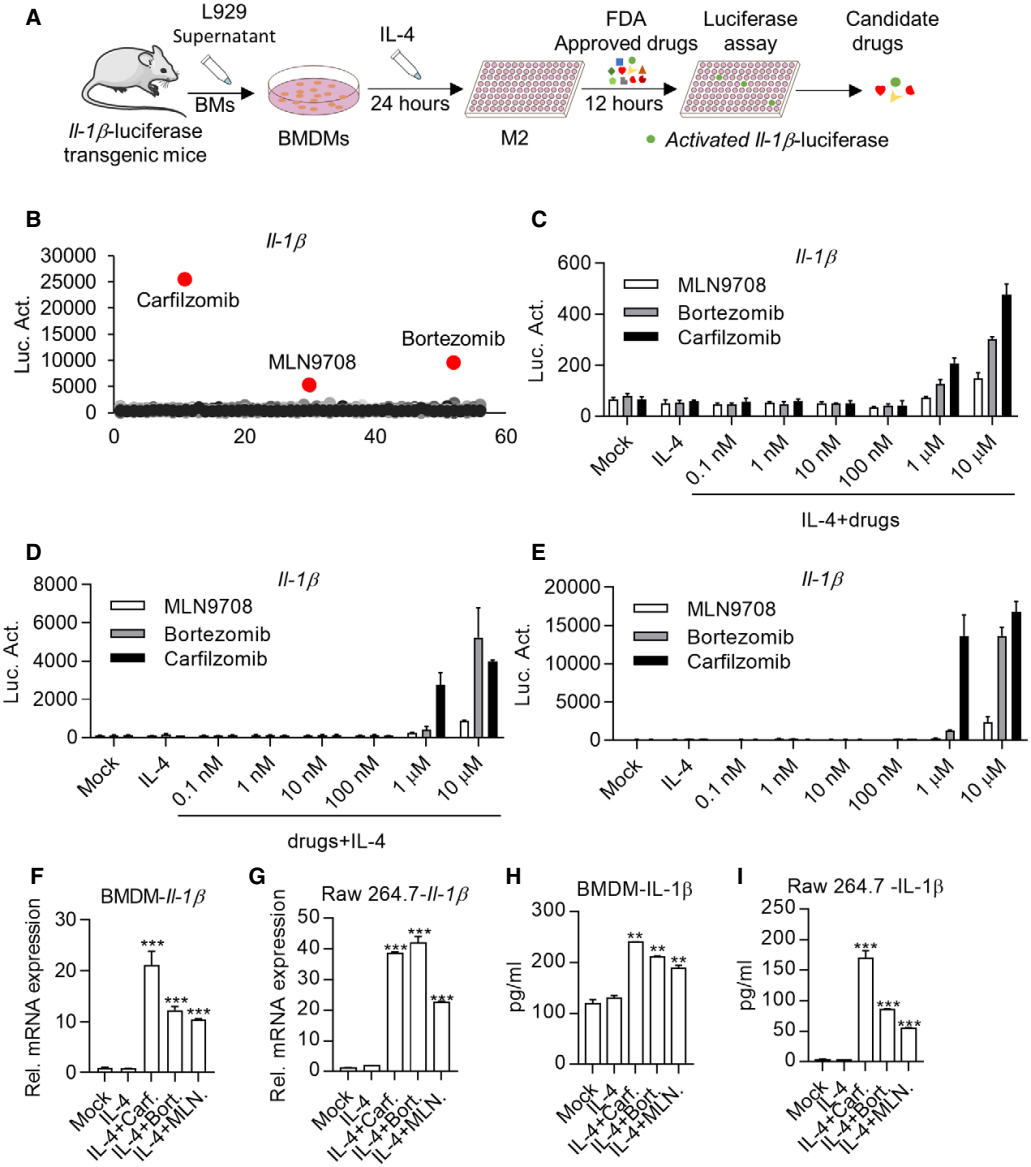
High‐throughput Screening of FDA drugs capable of inducing IL‐1β expression in M2 macrophages ASchematic diagram of high‐throughput screening.BCarfilzomib, Bortezomib, and MLN9708 activate luciferase activity in M2 macrophages derived from *Il‐1β*‐luciferase transgenic mice. BMDMs from IL‐1β‐luciferase transgenic mice were treated by IL‐4 (20 ng/ml) for 24 h to differentiate into mature M2 macrophages, and then used for screening of FDA compounds (5 μM). The X‐axis stands for the code of the drug.CCarfilzomib, Bortezomib, and MLN9708 activate *Il‐1β*‐luciferase in M2 macrophages in a dose‐dependent manner. BMDMs from *Il‐1β*‐luciferase transgenic mice were treated by IL‐4 (20 ng/ml) for 24 h, then treated by DMSO, Carfilzomib, Bortezomib, or MLN9708 at indicated concentrations. Luciferase activity was monitored 12 h later. Data from the three experiments are presented as the mean ± SD.DCarfilzomib, Bortezomib, and MLN9708 activate *Il‐1β*‐luciferase in a dose‐dependent manner as well as inhibit the transformation of M2 macrophages. BMDMs from *Il‐1β*‐luciferase transgenic mice were pretreated with DMSO, Carfilzomib, Bortezomib, or MLN970 at indicated concentrations for 1 h, followed by treatment with IL‐4 (20 ng/ml). Luciferase activity was monitored 12 h later. Data from three experiments are presented as the mean ± SD.ECarfilzomib, Bortezomib, and MLN9708 activate *Il‐1β*‐luciferase in M0 macrophages in a dose‐dependent manner. BMDMs from *Il‐1β*‐luciferase transgenic mice were stimulated by DMSO, Carfilzomib, Bortezomib, or MLN970 at indicated concentrations. Luciferase activity was monitored 12 h later. Data from three experiments are presented as the mean ± SD.F, GCarfilzomib, Bortezomib, and MLN9708 promote the mRNA expression of *Il‐1β* in M2 macrophages. BMDMs (F) or Raw264.7 cells (G) were pretreated by IL‐4 (20 ng/ml) for 24 h, then treated by DMSO, Carfilzomib (1 μM), Bortezomib (1 μM), or MLN9708 (2 μM). RNA was extracted from BMDMs or Raw264.7 cells 6 h after stimulation and the expression of *Il‐1β* was quantified through RT‐qPCR. Data from three experiments are presented as the mean ± SD. *T*‐test was used for statistical analysis of differences between groups. ***P < 0.001 (Student’s *t*‐test).H, ICarfilzomib, Bortezomib, and MLN9708 promote the secretion of inflammatory cytokine IL‐1β in M2 macrophages. BMDMs (H) or Raw264.7 cells (I) were pretreated by IL‐4 (20 ng/ml) for 24 h, then stimulated by DMSO, Carfilzomib (500 nM), Bortezomib (500 nM), or MLN9708 (500 nM). Secretion of IL‐1β was measured through ELISA 24 h later. Data from three experiments are presented as the mean ± SD. *T*‐test was used for statistical analysis of differences between groups. **P < 0.01, ***P < 0.001 (Student’s *t*‐test).

**Figure EV1:**
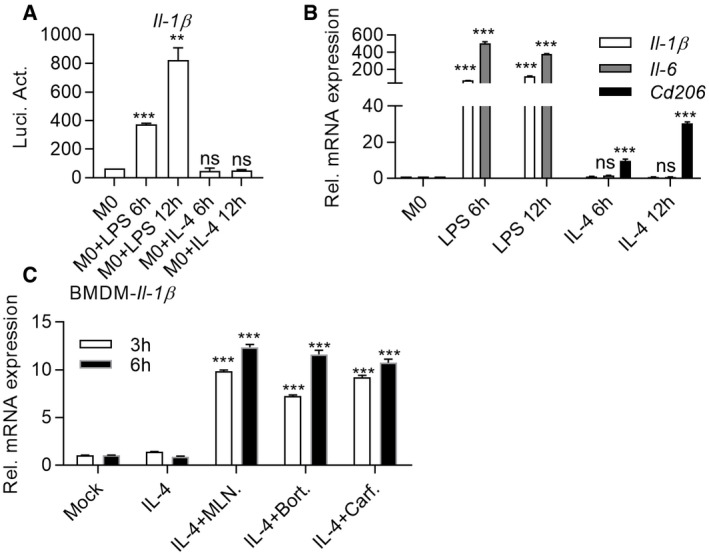
High‐throughput Screening of FDA drugs capable of inducing IL‐1β expression in M2 macrophages LPS but not IL‐4 significantly activates luciferase activity in M0 *Il‐1β*‐luciferase transgenic macrophages. BMDMs from *Il‐1β*‐luciferase transgenic mice were induced by LPS (100 ng/ml) or IL‐4 (20 ng/ml). Luciferase assays were performed 6 or 12 h after stimulation. Data are means ± SD of three independent experiments. **P < 0.01, ***P < 0.001 (Student’s *t*‐test).M1 or M2 markers induced in macrophages by LPS or IL‐4. BMDMs were induced by LPS (100 ng/ml) or IL‐4 (20 ng/ml) for indicated time. RT‐qPCR was then performed to quantify the expression of M1/M2 markers. Data are means ± SD of three independent experiments. ***P < 0.001 (Student’s *t*‐test).Carfilzomib, Bortezomib, and MLN9708 promote the expression of *Il‐1β*. BMDMs were pretreated by IL‐4 (20 ng/ml) for 24 h, followed by stimulation with DMSO, Carfilzomib (1 μM), Bortezomib (1 μM), or MLN9708 (2 μM) for indicated time. RNA was extracted from cells for quantification of *Il‐1β* expression through RT‐qPCR. Data are means ± SD of three independent experiments. ***P < 0.001 (Student’s *t*‐test).

To repurpose FDA‐approved drugs for reprogramming M2 macrophages into M1 cells, we performed a high‐throughput screening to identify drugs capable of activating *Il‐1β* expression in IL‐4‐activated BMDMs derived from transgenic mice, in which the luciferase reporter gene was placed under the control of *Il‐1β* promoter (*Il‐1β*‐luc) (Li *et al*, 2008) (Figs 1A and EV1A and B). These efforts led to identification of Carfilzomib, Bortezomib, and MLN9708, which effectively activated the *Il‐1β* promoter activity in M2 macrophages (Fig 1B). Further experiments showed that these drugs activated *Il‐1β* promoter activity in IL4‐induced M2 macrophages in dose‐ and time‐dependent manners (Figs 1C and EV1C). We also found that pretreatment with these drugs endowed M0 macrophages the ability to resist M2 induction by IL‐4 and kept M1 characteristics as indicated by strong *Il‐1β* promoter activity (Fig 1D). Interestingly, these drugs, by their own, activated expression of *Il‐1β* on M0 macrophages as judged by luciferase activity (Fig 1E).

To confirm that IL‐1β is indeed produced as suggested by its promoter activity, we quantified mRNA and protein levels of IL‐1β. Consistently, we found that these drugs strongly activated transcription of *Il‐1β* gene in IL4‐activated BMDMs and Raw264.7 cells (Fig 1F and G). Likewise, ELISA (enzyme‐linked immunosorbent assay) revealed high level of protein secreted by both cell lines treated by these drugs (Fig 1H and I).

In all cases, Carfilzomib consistently elicited the strongest IL‐1β expression in macrophages. Therefore, we focused our further experimental efforts on Carfilzomib unless stated. We named macrophages initially activated with IL‐4, followed by Carfilzomib treatment that have transformed from M2 to M1‐like macrophages as Ci‐M1 (for Carfilzomib‐induced M1‐like cells).

### Carfilzomib reprogrammed M2 macrophages into M1‐like population

**Figure 2:**
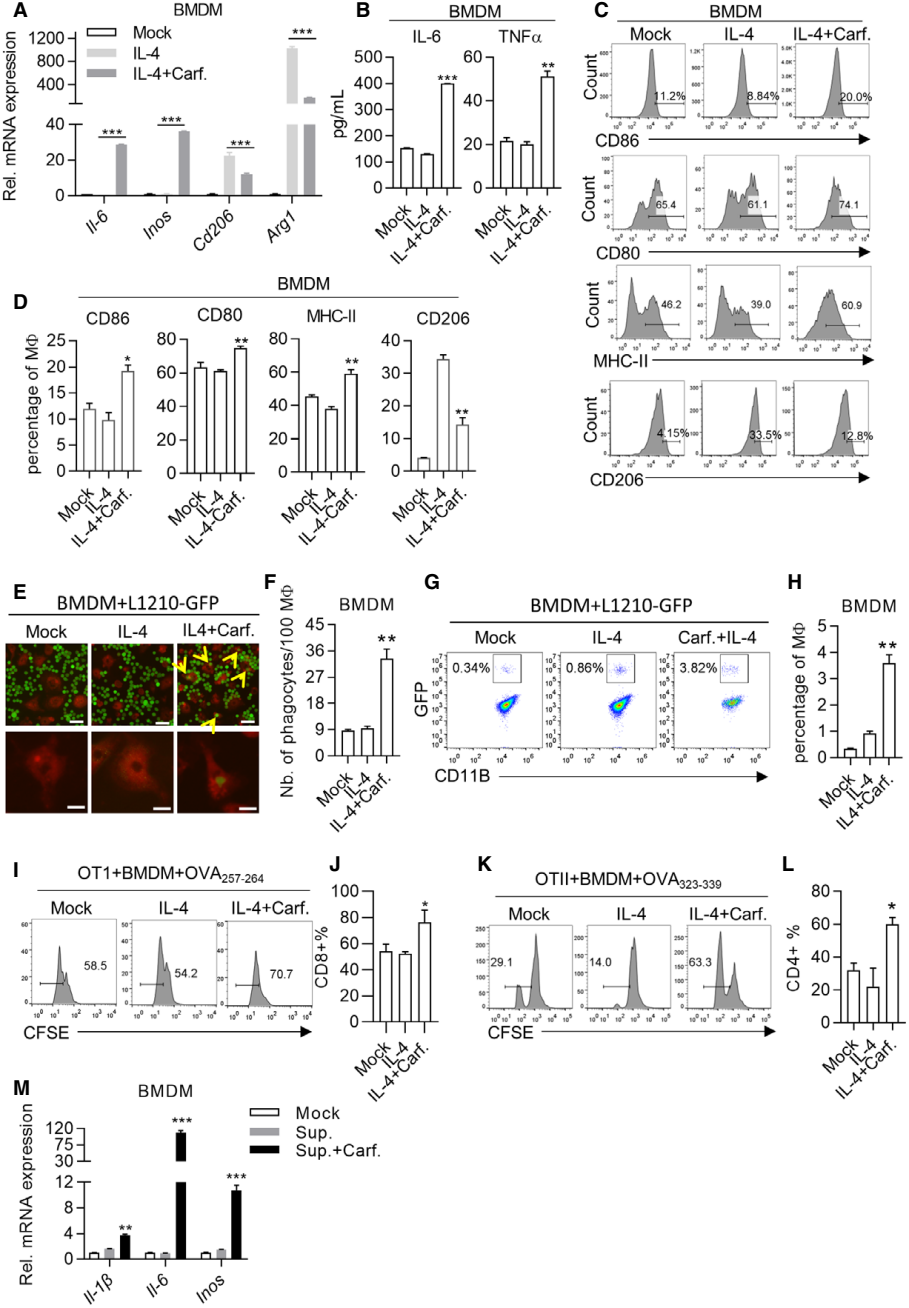
Carfilzomib reprogrammed M2 macrophages into M1‐like population AThe most effective one among the candidate compounds, Carfilzomib, significantly promotes the expression of M1 macrophage markers, as well as reduces the expression of M2 macrophage markers. BMDMs were pretreated by IL‐4 (20 ng/ml) for 24 h, then stimulated by DMSO or Carfilzomib (1 μM). RNA was extracted from cells 6 h after stimulation and the expression of M1 (*Il‐6*/*Inos*) or M2 (*Cd206*/*Arg1*) macrophage markers was quantified through RT‐qPCR. Data from three experiments are presented as the mean ± SD. *T*‐test was used for statistical analysis of differences between groups. ***P < 0.001 (Student’s *t*‐test).BCarfilzomib significantly promotes the secretion of IL‐6 and TNFα in M2 macrophages. BMDMs were pretreated by IL‐4 (20 ng/ml) for 24 h, then stimulated by DMSO or Carfilzomib (500 nM). The secretion of IL‐6 and TNFα was detected through ELISA 24 h after stimulation. Data from three experiments are presented as the mean ± SD. *T*‐test was used for statistical analysis of differences between groups. **P < 0.01, ***P < 0.001 (Student’s *t*‐test).CCarfilzomib significantly promotes the expression of membrane protein CD86, CD80, and MHC‐II and reduces the expression of CD206. BMDMs were pretreated by IL‐4 (20 ng/ml) for 24 h, then stimulated by DMSO or Carfilzomib (500 nM). The representative histogram of CD86, CD80, MHC‐II, and CD206 expression was analyzed through flow cytometry 12 h after stimulation. The gating of CD86^+^, CD80^+^, CD206^+^, and MHC‐II^+^ population was determined against those of isotype‐matched staining control.DStatistics for proportion of CD86‐, CD80‐, MHC‐II‐, or CD206‐positive cells in BMDMs. Data are means ± SD of three independent experiments. *P < 0.05, **P < 0.01 (Student’s *t*‐test).ECarfilzomib promotes the phagocytosis of macrophages. BMDMs were pretreated by IL‐4 (20 ng/ml) for 24 h, then stimulated by DMSO or Carfilzomib (500 nM) for 12 h. After starving for 2 h and stained with Red membrane dye, BMDMs (red) were incubated with L1210‐GFP cells (green) in serum‐free medium for another 2 h. Phagocytosis effect was observed and photographed under fluorescence microscope. Scale bars: 50 μm (up) and 20 μm (down). The yellow arrows indicated L1210 that is phagocytosed by macrophages.FStatistics represent the number of phagocytosis L1210 in 100 macrophages. Data are means ± SD of three independent experiments. **P < 0.01 (Student’s *t*‐test).G, HPhagocytosis is measured by flow cytometry. After co‐incubation, BMDMs were stained with fluorochrome‐conjugated anti‐CD11B antibody and analyzed through flow cytometry. Phagocytosing efficiency was determined as the percentage of CD11B and GFP double‐positive population. Statistics represents the percentage of phagocytosing against total macrophage population. Statistics represents the percentage of phagocytosing against total macrophage population and data are means ± SD of three independent experiments. **P < 0.01 (Student’s *t*‐test).I–LCi‐M1 drives proliferation of CD8^+^ or CD4^+^ T cells. BMDMs treated as indicated were transfected with 10 μg/ml of OVA_257‐264_ peptide or OVA_323‐339_ peptide for 1 h. BMDMs were then washed and co‐incubated with CFSE‐labeled OT‐I or OT‐II cells, respectively. After 72 h, OT‐I (I) or OT‐II (K) cells were stained with anti‐CD8a or anti‐CD4 fluorochrome‐conjugated antibodies and CFSE was detected through flow cytometry to evaluate the dilution of CFSE in T cells. Statistics represents the percentage of divided T cells. (J, L) are statistical analysis of Fig 2I and K. The data are means ± SD of three independent experiments. *P < 0.05 (Student’s *t*‐test).MCarfilzomib promotes the M1 polarization of tumor‐associated macrophages *in vitro*. BMDMs were cultured with tumor culture supernatant (TSN) produced by L1210 cells, followed by activation with DMSO or Carfilzomib (1 μM). RNA was extracted from cells and the expression of *Il‐1β*, *Il‐6,* and *Inos* was quantified through RT‐qPCR 6 h after stimulation. The data are means ± SD of three independent experiments. **P < 0.01, ***P < 0.001 (Student’s *t*‐test).

**Figure EV2:**
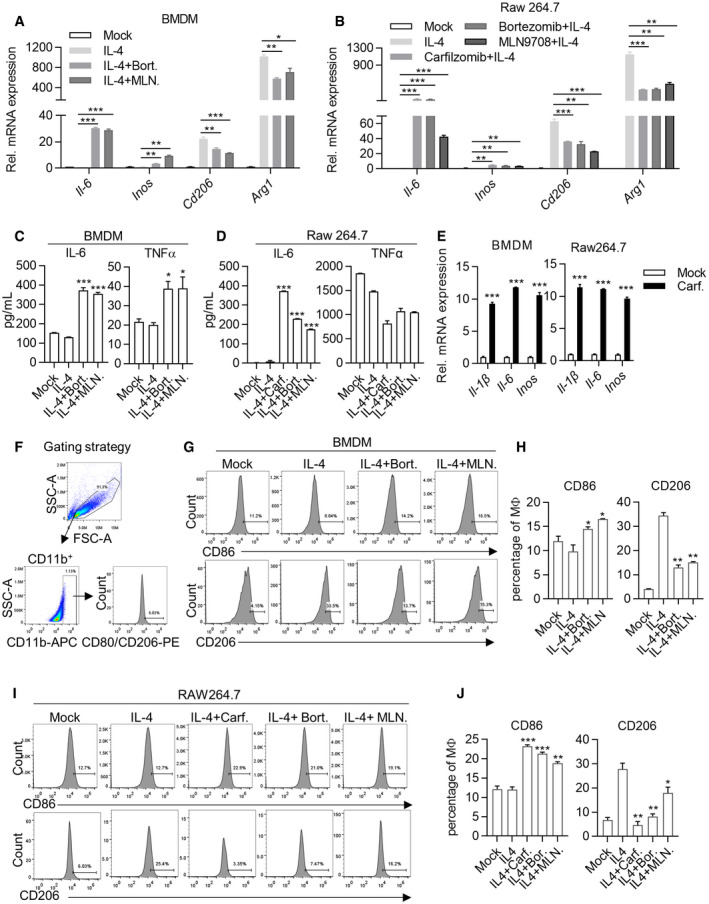
Carfilzomib, Bortezomib and MLN9708 reprogram M2 macrophages into M1‐like population A, BCarfilzomib, Bortezomib, and MLN9708 promote the expression of M1 macrophage markers and reduce the expression of M2 macrophage markers in macrophages. BMDMs (A) and Raw264.7 cells (B) were treated as Fig 1F and G. RNA was extracted from cells and the expression of M1 (*Il‐6/Inos*) or M2 (*Cd206/Arg1*) macrophage markers was quantified through RT‐qPCR 6 h after stimulation. Data are means ± SD of three independent experiments. *P < 0.05, **P < 0.01, ***P < 0.001 (Student’s *t*‐test).C, DCarfilzomib, Bortezomib, and MLN9708 promote the secretion of IL‐6 and TNFα in M2 macrophages. BMDMs (C) and Raw264.7 cells (D) were pretreated by IL‐4 (20 ng/ml) for 24 h, then stimulated by DMSO, Carfilzomib (500 nM), Bortezomib (500 nM), or MLN9708 (500 nM). Secretion of IL‐6 and TNFα in culture media was detected through ELISA 24 h after stimulation. Data are means ± SD of three independent experiments. *P < 0.05, ***P < 0.001 (Student’s *t*‐test).ECarfilzomib alone promotes the expression of proinflammatory cytokines. BMDMs and Raw264.7 cells were treated by DMSO or Carfilzomib (1 μM) for 6 h, then RNA was extracted from cells, and the expression of *Il‐1β*/*Il‐6/Inos* were quantified through RT‐qPCR. Data are means ± SD of three independent experiments. ***P < 0.001 (Student’s *t*‐test).FGating strategy for analyzing M1/M2 macrophage surface markers in macrophages. CD86‐ or CD206‐positive cells were gated on CD11B^+^ cells.G–JCarfilzomib, Bortezomib, and MLN9708 promote the expression of membrane protein CD86 and reduce CD206. BMDMs (G) and Raw264.7 cells (I) were treated as described in Fig S2C and D. The representative histogram of CD86 and CD206 expression was shown for flow cytometry 12 h after stimulation. (H, J) Statistics represent the proportion of CD86‐ or CD206‐positive cells in BMDMs (H) or Raw264.7 cells (J) under different treatment conditions. Data are means ± SD of three independent experiments. *P < 0.05, **P < 0.01, ***P < 0.001 (Student’s *t*‐test).

We went on to test whether Ci‐M1 behaved like typical M1 macrophages. Classically activated M1 macrophages have been reported to express large amounts of inflammatory cytokines while expressing low or no level of anti‐inflammatory cytokine (Fig EV1B). We found that Ci‐M1, reprogrammed from both IL‐4‐activated BMDMs and Raw264.7, expressed high level of M1 biomarkers and inhibited M2 biomarkers at mRNA level (Figs 2A and EV2A and B). ELISA confirmed that Ci‐M1 cells secreted inflammatory cytokines like IL‐6 and TNFα (Figs 2B and EV2C and D). Consistent with results shown in Fig 1E, Carfilzomib activated M0 macrophages to express high amounts of proinflammatory cytokines (*Il‐1β*, *Il‐6,* and *Inos*) at mRNA level (Fig EV2E). We also found that Ci‐M1 expressed elevated levels of CD86, CD80, and MHC‐II (M1 surface marker) and reduced levels of CD206 (M2 surface marker) in comparison to IL‐4‐activated M2 macrophages (Figs 2C and D, and EV2F–J). Of note, Ci‐M1 reprogrammed from RAW264.7 expressed limited level of TNFα protein, suggestive of difference between RAW264.7 and BMDM (Fig EV2C and D). We also found that Carfilzomib did not significantly alter the expression of MHC‐I in M2 macrophages (Appendix Fig S1A and B).

M1 macrophages exhibit enhanced phagocytosis and ability to present antigen to T cells. We then went on to test these activities. For this purpose, macrophages were stained with CellMask™ Deep Red and co‐cultured with GFP expressing L1210, a lymphocytic leukemia cell line (designated L1210‐GFP), such that phagocytosing macrophages can be captured under fluorescence microscopy as red cells containing green vacuoles. We found that Ci‐M1 exhibited enhanced phagocytosis as compared to M2 macrophages (Fig 2E and F, and Appendix Fig S1C and D). This was further confirmed through FACS analysis (Fig 2G and H).

Our FACS analysis with 25‐D1.16, an antibody reacting with the ovalbumin‐derived peptide SIINFEKL bound to H‐2Kb (MHC‐I), revealed that Ci‐M1 cells exhibited enhanced ability to present antigens (Appendix Fig S1E and F). Significantly, we found that Ci‐M1 loaded with respective peptides drove more robust proliferation of OT‐I and OT‐II cells than peptide‐loaded M2 macrophages (Fig 2I–L). Notably, we excluded the possibility that Carfilzomib *per se* promoted proliferation of CD4^+^ and CD8^+^ T cells (Appendix Fig S1G and H). Collectively, our results argued that Ci‐M1 cells exhibited stronger antigen‐presenting ability.

As an *in vitro* model for inducing TAM, tumor cell culture supernatants (TSN) are able to educate monocytes to become immunosuppressive macrophage (Kuang *et al*, 2007; Zhou *et al*, 2009). To confirm Carfilzomib could promote TAM polarization to Ci‐M1, BMDM and Raw264.7 were first exposed to TSN for TAM differentiation, following protocols reported earlier (Kuang *et al*, 2007). The resultant TAMs were then treated with Carfilzomib. Our results showed that Carfilzomib potently transformed TAM to Ci‐M1 (Fig 2M and Appendix Fig S1I–K). Of note, Carfilzomib treatment increased CD206 expression in TAMs, suggesting the microenvironment‐dependent effects (Appendix Fig S1I–K).

Collectively, Carfilzomib potently transforms M2 and TAMs into M1‐like macrophages. Notably, Bortezomib and MLN9708 had similar capacity, although they were less potent (Fig EV2A–D and G–J, and Appendix Fig S1C–F).

### Proteasome inhibitors reprogrammed M2 macrophages toward M1‐like macrophages by inducing ER stress signaling

**Figure 3:**
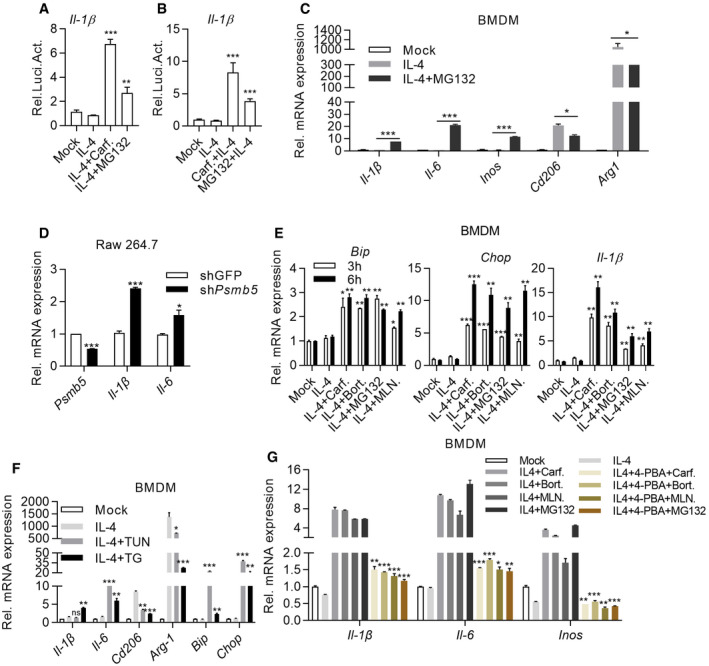
Proteasome inhibitors reprogram M2 macrophages toward M1‐like macrophages by inducing ER stress signaling A, BCarfilzomib and proteasome inhibitor MG132 activate *Il‐1β* luciferase in macrophages. (A) BMDMs were treated with IL‐4 (20 ng/ml) for 24 h and then stimulated by DMSO, Carfilzomib (1 μM), or MG132 (5 μM). (B) BMDMs were stimulated by DMSO, Carfilzomib (1 μM), or MG132 (5 μM)) for 1 h, then induced with IL‐4 (20 ng/ml). Luciferase assays were monitored 12 h after stimulation. The data are means ± SD of three independent experiments. ***P < 0.001 (Student’s *t*‐test).CMG132 promotes the expression of M1 macrophage markers and inhibits the expression of M2 macrophage markers. BMDMs were pretreated with IL‐4 (20 ng/ml) for 24 h and then stimulated by DMSO or MG132 (5 μM). RNA was extracted from BMDMs 6 h after stimulation and the expression of M1 (*Il‐1β*/*Il‐6*/*Inos*) or M2 (*Cd206*/*Arg1*) macrophage markers was quantified through RT‐qPCR. The data are means ± SD of three independent experiments. *P < 0.05, ***P < 0.001 (Student’s *t*‐test).DKnockdown of β5 subunit of proteasome in macrophages promotes the expression of proinflammatory factors. RNA was extracted from Raw264.7 (shGFP or sh*Psmb5*) and the expression of *Il‐1β* and *Il‐6* was quantified through RT‐qPCR. The data are means ± SD of three independent experiments. *P < 0.05, ***P < 0.001 (Student’s *t*‐test).ECarfilzomib, Bortezomib, MLN9708, and MG132 induce ER stress response. BMDMs were pretreated with IL‐4 (20 ng/ml) for 24 h and then stimulated by DMSO or Carfilzomib (1 μM), Bortezomib (1 μM), MLN9708 (2 μM), and MG132 (5 μM). RNA was extracted from cells 3 or 6 h after stimulation and the expression of ER stress‐related genes (*Bip*, *Chop*) was detected through RT‐qPCR. The data are means ± SD of three independent experiments. *P < 0.05, **P < 0.01, ***P < 0.001 (Student’s *t*‐test).FER stress agonists, TUN and TG, promote the expression of M1 macrophage markers, as well as reduce the expression of M2 macrophage markers. BMDMs were pretreated with IL‐4 (20 ng/ml) for 24 h and then stimulated by DMSO, TUN (500 nM), or TG (500 nM). RNA was extracted from cells 6 h after stimulation and indicated genes were quantified through RT‐qPCR. The data are means ± SD of three independent experiments. *P < 0.05, **P < 0.01, ***P < 0.001 (Student’s *t*‐test).GInhibition of ER stress impairs the ability of Carfilzomib, Bortezomib, MLN9708, and MG132 to reprogram M2 toward M1‐like macrophages. BMDMs were induced by IL‐4 (20 ng/ml) for 24 h, then pretreated with 4‐PBA (5 mM) for 1 h and stimulated by Carfilzomib (1 μM), Bortezomib (1 μM), MLN9708 (2 μM), and MG132 (5 μM). RNA was extracted from cells 6 h after stimulation and the expression of *Il‐1β*, *Il‐6,* and *Inos* was detected through RT‐qPCR. The data are means ± SD of three independent experiments. *P < 0.05, **P < 0.01, ***P < 0.001 (Student’s *t*‐test).

Interestingly, Carfilzomib, Bortezomib, and MLN9708 are all proteasome inhibitors (PIs), which are the only three PIs in our library. Likewise, MG132, a tool compound for proteasomal inhibition, was also able to activate *Il‐1β* promoter activity in IL‐4‐activated M2 macrophages (Fig 3A and B), suggesting that proteasomal inhibition transformed M2 to M1‐like macrophages. Q‐PCR analysis indicated that MG132 was able to activate M2 macrophages to express *Il‐1β*, *Il‐6,* and *Inos*, and inhibit the expression of M2 macrophage markers, such as *Cd206* and *Arg1* (Fig 3C). To further show whether proteasomal inhibition *per se* is the driving force for reprogramming M2 into M1‐like macrophages, we went on to genetically disable proteasomal subunit. Interestingly, Psmb5 knockdown upregulated expression of *Il‐1β* and *Il‐6* in IL‐4 activated Raw264.7 cells (Fig 3D), consistent with an earlier observation in THP‐1 cells (Wang *et al*, 2017). Of note, *Cd206* and *Arg‐1* expression remained upregulated, suggestive of difference between chemical inhibition and genetic inhibition of proteasomal activity (Appendix Fig S2A). Taken together, these data indicated that PIs were able to reprogram M2 macrophages toward M1‐like macrophages through inhibition of proteasomal activity.

Previous studies indicated that PIs caused the accumulation of misfolded proteins, leading to endoplasmic reticulum (ER) stress and thereby eliciting the unfolded protein response (UPR) (Obeng *et al*, 2006). Q‐PCR analysis revealed that besides *Il‐1β*, PIs were able to induce expression of typical UPR biomarkers, *Bip* and *Chop*, in IL‐4‐activated M2 macrophage (Fig3E and Appendix Fig S2B). These data indicated that PIs might mediate macrophage polarization through ER stress. We were able to further confirm this point, since classic ER stress inducers, tunicamycin (TUN) and thapsigargin (TG), induced expression of M1 markers, including *Il‐1β*, *Il‐6* alongside ER stress markers in IL‐4‐activated M2 macrophage (Fig 3F and Appendix Fig S2C). Meanwhile, the expression of M2 markers, *Cd206* and *Arg1*, was significantly inhibited (Fig 3F). Sodium 4‐phenylbutyrate (4‐PBA) has been reported to effectively alleviate ER stress (Rubenstein & Zeitlin, 2000). Consistently, we found that 4‐PBA treatment inhibited upregulation of M1 markers elicited by PIs in M2 macrophages derived from BMDM (Fig 3G). Taken together, these results suggested that PIs reprogrammed M2 macrophages into M1‐like macrophages by inducing ER stress signaling.

### IRE1α‐TRAF2‐NF‐κB axis is essential for polarizing M2 to M1‐like macrophages

**Figure EV3:**
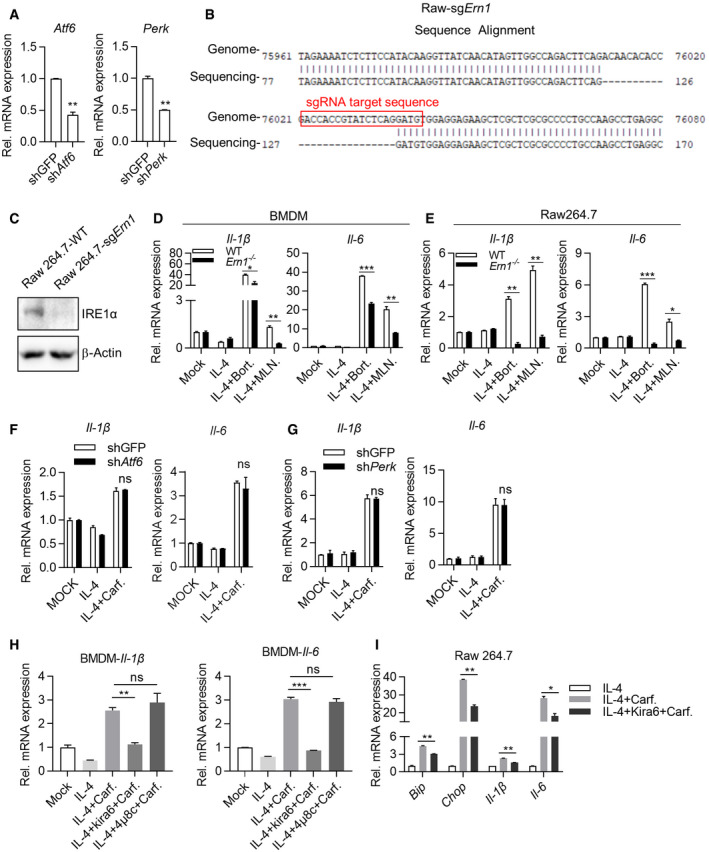
IRE1α‐TRAF2‐NF‐κB axis is essential for polarizing M2 to M1‐like macrophages AKnockdown efficiency of sh*Atf6* or sh*Perk*. RNA was extracted from Raw264.7‐shGFP, Raw264.7‐sh*Atf6*, and Raw264.7‐sh*Perk* cells, and the expression of *Atf6* and *Perk* was quantified through RT‐qPCR. Data are means ± SD of three independent experiments. **P < 0.01 (Student’s *t*‐test).BDetails of sgRNA‐mediated knockout of IRE1α in Raw264.7.CIRE1α knockout efficiency of sgRNA. Raw264.7‐sg IRE1α monoclonal cells were picked out and the WCLs were analyzed by immunoblots with the indicated antibodies.D, EDeficiency of *Ern1* represses the expression of inflammatory‐related genes activated by Bortezomib, MLN9708. Wild‐type and *Ern1* ^−/−^ BMDMs (D) or Raw264.7 cells (E) were pretreated by IL‐4 (20 ng/ml) for 24 h, and then stimulated with DMSO, Bortezomib (1 μM), or MLN9708 (2 μM) for 6 h. Expression of *Il‐1β* and *Il‐6* was quantified through RT‐qPCR. Data are means ± SD of three independent experiments. *P < 0.05, **P < 0.01, ***P < 0.001 (Student’s *t*‐test).F, GKnockdown of *Atf6* (F) or *Perk* (G) does not significantly change the ability of Carfilzomib to promote M1‐like macrophages polarization. Engineered cell lines, including Raw264.7‐shGFP, Raw264.7‐sh*Atf6,* or Raw264.7‐sh*Perk*, were pretreated by IL‐4 (20 ng/ml) for 24 h, then stimulated by DMSO or Carfilzomib (1 μM). RNA was extracted from cells and the expression of *Il‐1β* and *Il‐6* was quantified through RT‐qPCR 6 h after stimulation. Data are means ± SD of three independent experiments.HIRE1α kinase activity plays a role in mediating expression of M1 markers induced by Carfilzomib in M2 macrophages. BMDMs were activated by IL‐4 (20 ng/ml) for 24 h, followed by treatment with IRE1α inhibitors including Kira6, 4μ8c for 1 h, and then stimulated by Carfilzomib (1 μM) for 6 h. Expression of *Il‐1β* and *Il‐6* was quantified through RT‐qPCR. Data are means ± SD of three independent experiments. **P < 0.01, ***P < 0.001 (Student’s *t*‐test).IImpact of inhibition of IRE1α kinase activity on the expression of M1 marker genes and ER stress‐related genes in Raw264.7 activated by Carfilzomib. Raw264.7 cells were activated with IL‐4 (20 ng/ml) for 24 h, followed by treatment with Kira6 for 1 h, and then stimulated by Carfilzomib (1 μM) for 6 h. The mRNA expression of *Bip, Chop, Il‐1β,* and *Il‐6* was quantified through RT‐qPCR. Data are means ± SD of three independent experiments. *P < 0.05, **P < 0.01 (Student’s *t*‐test). Source data are available online for this figure.

**Figure 4:**
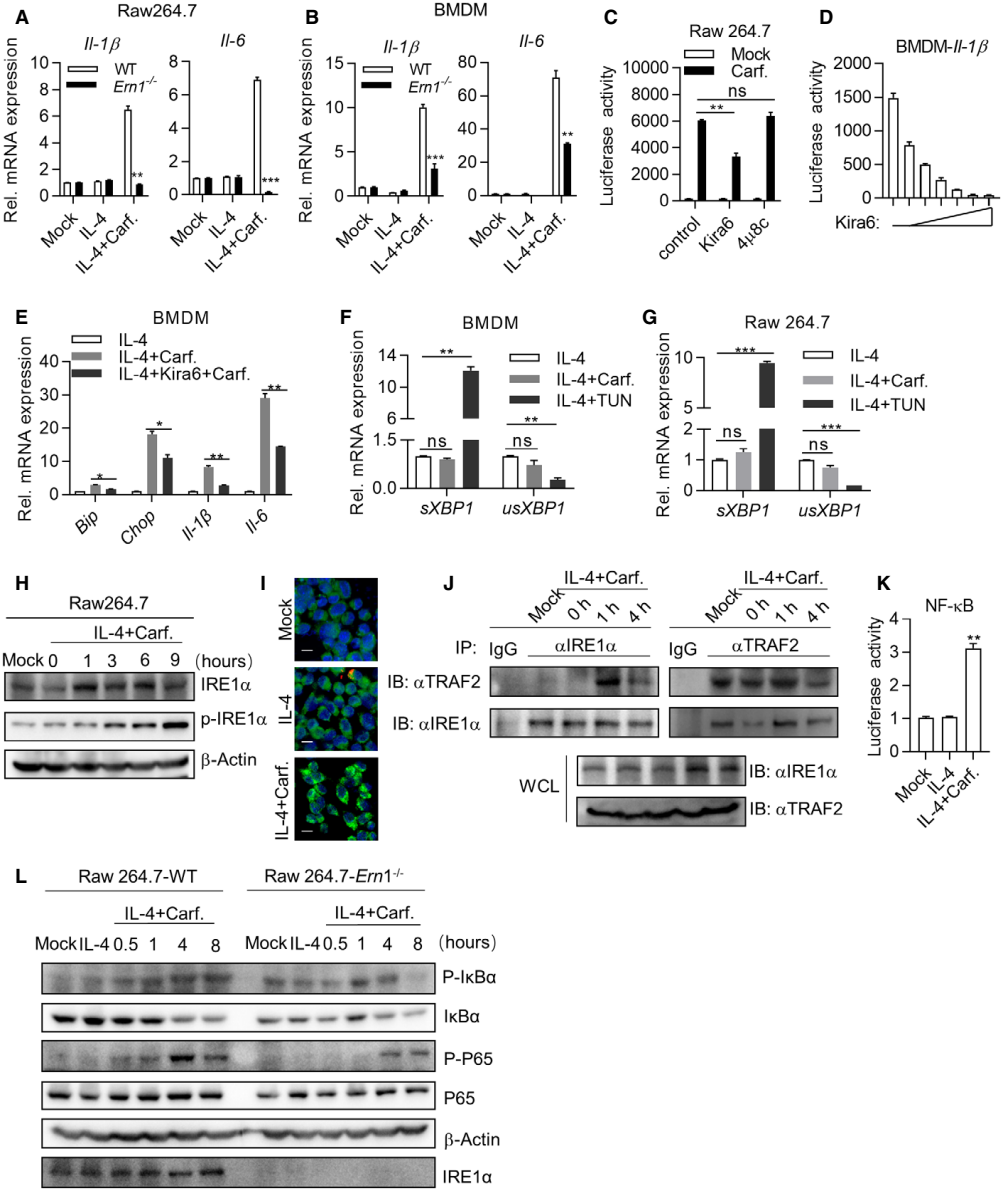
IRE1α‐TRAF2‐NF‐κB axis is essential for polarizing M2 to M1‐like macrophage A, BDeficiency of *Ern1* reduces the expression of inflammatory‐related genes. Wild‐type and *Ern1* ^−/−^ Raw264.7 cells (A) or BMDMs (B) were pretreated by IL‐4 (20 ng/ml) for 24 h, then stimulated by DMSO or Carfilzomib (1 μM). The mRNA of *Il‐1β* and *Il‐6* was quantified through RT‐qPCR 6 h after stimulation. The data are means ± SD of three independent experiments. **P < 0.01, ***P < 0.001 (Student’s *t*‐test).CInhibition of IRE1α kinase activity represses luciferase of Carfilzomib‐treated M2 macrophages derived from *Il‐1β*‐luciferase transgenic mice. BMDMs derived from *Il‐1β*‐luciferase transgenic mice were differentiated into M2 macrophages by IL‐4 (20 ng/ml). M2 macrophages were treated by different inhibitors of IRE1α, including Kira6 (inhibitor of IRE1α kinase activity, 100 nM) and 4μ8c (inhibitor of IRE1α RNase activity, 100 nM) for 1 h, followed by stimulation with Carfilzomib (1 μM) for 12 h. Luciferase activity was then monitored. The data are means ± SD of three independent experiments. **P < 0.01 (Student’s *t*‐test).DKira6 inhibits Carfilzomib‐induced *Il‐1β*‐luciferase in M2 macrophages in a dose‐dependent manner. *Il‐1β*‐luciferase BMDMs were differentiated into M2 macrophages by IL‐4 (20 ng/ml), treated by Kira6 at indicated concentrations (0, 50, 100, 200, 400, 800, and 1,600 nM) for 1 h, and then stimulated by Carfilzomib (1 μM) for 12 h. Luciferase activity was measured. The data are means ± SD of three independent experiments.EKira6 inhibits the expression of ER stress‐related genes and inflammatory‐related genes. BMDMs were treated by IL‐4 (20 ng/ml) for 24 h, treated with Kira6 (100 nM) for 1 h, and then stimulated by Carfilzomib (1 μM) for 6 h. RNA was extracted from cells and the expression of *Bip, Chop, Il‐1β,* and *Il‐6* was quantified through RT‐qPCR. The data are means ± SD of three independent experiments. *P < 0.05, **P < 0.01 (Student’s *t*‐test).F, GCarfilzomib has no effect on splicing of *XBP1* in M2 macrophages. BMDMs (F) or Raw264.7 cells (G) were pretreated by IL‐4 (20 ng/ml) for 24 h, then stimulated with DMSO, Carfilzomib (1 μM), or TUN (500 nM). The mRNA expression of *sXBP1* and *unXBP1* was detected through RT‐qPCR 6 h after stimulation. The data are means ± SD of three independent experiments. *P < 0.05, **P < 0.01, ***P < 0.001 (Student’s *t*‐test).HCarfilzomib increases the expression of total IRE1α as well as promotes the phosphorylation of IRE1α. BMDMs were pretreated by IL‐4 (20 ng/ml) for 24 h, then stimulated by DMSO or Carfilzomib (1 μM) for indicated time. Whole cell lysates (WCL) were analyzed by immunoblots (IB) with the indicated antibodies.ICarfilzomib promotes the oligomerization of IRE1α in macrophages. Raw264.7 cells were pretreated by IL‐4 (20 ng/ml) for 24 h, then stimulated by DMSO or Carfilzomib (1 μM) for 2 h. The cells were fixed and stained with anti‐IRE1α antibody and fluorochrome‐conjugated secondary antibody, and then subjected to confocal microscopy. Representative images show the aggregation of IRE1α (green). Scale bar: 10 μm.JCarfilzomib promotes the association between IRE1α and TRAF2 in M2 macrophages. Raw264.7 cells were pretreated by IL‐4 (20 ng/ml) for 24 h, then stimulated by Carfilzomib (1 μM) for 2 or 4 h. Co‐immunoprecipitation (Co‐IP) and IB were performed with indicated antibodies.KCarfilzomib activates NF‐κB promoter in Raw264.7 cells. Raw264.7 cells were pretreated with or without IL‐4 (20 ng/ml) for 24 h, NF‐κB reporter and TK were co‐transfected for overnight, then stimulated by DMSO or Carfilzomib (1 μM) for 12 h before luciferase assay performed. The data are means ± SD of three independent experiments. **P < 0.01 (Student’s *t*‐test).LCarfilzomib activates NF‐κB signaling pathway through IRE1α in M2 macrophages. Wild‐type and *Ern1* ^−/−^ Raw264.7 cells were pretreated by IL‐4 (20 ng/ml) for 24 h, then stimulated by Carfilzomib (1 μM) for indicated time points. WCL were analyzed by IB with indicated antibodies.

In mammals, three sensors (IRE1α, PERK, and ATF6) detect unfolded protein accumulation in endoplasmic reticulum (ER) and activate UPR to reduce unfolded protein load to maintain cell viability and function (Hetz, 2012). To identify which sensor is responsible for reprogramming M2 macrophages by PIs, we knockdown expression of ATF6 and PERK with shRNA (Fig EV3A) and knockout IRE1α through sgRNA‐mediated targeting (Fig EV3B and C). Q‐PCR assays indicated that expression of *Il‐1β* and *Il‐6* was markedly impaired in Ci‐M1 cells derived from IRE1α‐deficient Raw264.7 cells (Fig 4A) while no significant impacts were detected in shPERK‐ or shATF6‐treated RAW264.7 cells (Fig EV3F and G). To further confirm this point, we crossed LyzM‐cre mice with ERN1 (encoding IRE1α)^fl/fl^ for generating IRE1α‐deficient (*Ern1* ^−/−^) macrophages. Indeed, *Ern1* ^−/−^ BMDMs were largely unresponsive to Carfilzomib treatment in terms of upregulating M1 biomarkers (Fig 4B), as well as to Bortezomib and MLN9708 (Fig EV3D and E).

IRE1α can be activated to exert RNase activity and kinase activity, which could be inhibited by chemical inhibitors. Interestingly, Kira6 (inhibiting IRE1 kinase activities), but not 4μ8C (inhibiting IRE1 RNase activities), dose dependently inhibited luciferase activities in Ci‐M1 derived from *Il‐1β*‐luciferase transgenic mice (Figs 4C and D, and EV3H). Q‐PCR assay further confirmed critical role of kinase activity of IRE1α in mediating PI’s induction of M1 biomarkers. Consistently, besides *Bip* and *Chop*, Kira6 inhibited the transcription of *Il‐1β* and *Il‐6* by Ci‐M1 derived from both BMDM and Raw264.7 (Figs 4E and EV3I). Of note, we found that Carfilzomib did not activate XBP1 splicing in M2 macrophages, which was in drastic contrast to classic ER stress inducers like tunicamycin (Fig 4F and G). Taken together, these results indicated that kinase activity of IRE1α played an important role in reprogramming M2 into M1 macrophages by Carfilzomib.

Unfolded proteins in ER bind to luminal domain of IRE1α, resulting in oligomerization, autophosphorylation of IRE1a, and activation of its RNase activity (Adams *et al*, 2019). IRE1α recruits TRAF2 and activates the downstream IKK‐NF‐κB pathway to induce inflammation cytokines production (Kaneko *et al*, 2003; Hu *et al*, 2006). We found that expression and phosphorylation of IRE1α were significantly increased in Ci‐M1 (Fig 4H). Confocal analysis revealed that Carfilzomib induced robust aggregation of IRE1α in IL‐4‐activated M2 macrophages (Fig 4I). We further found that Carfilzomib enhanced recruitment of TRAF2 by IRE1α (Fig 4J).

Furthermore, we found that Carfilzomib treatment induced robust NF‐κB transcriptional activity in a reporter assay (Fig 4K). Moreover, Carfilzomib‐induced phosphorylation of IκBα and p65, hallmarks of activation of NF‐κB pathways, was drastically impaired in *Ern1* ^−/−^ M2 macrophages (Fig 4L).

Taken together, these results suggest that IRE1α‐TRAF2‐NF‐κB axis is essential for Carfilzomib to reprogram M2 into M1‐like macrophages.

### Carfilzomib shrank tumor *in vivo* through promoting M2 macrophages polarization into M1‐like macrophages

**Figure 5:**
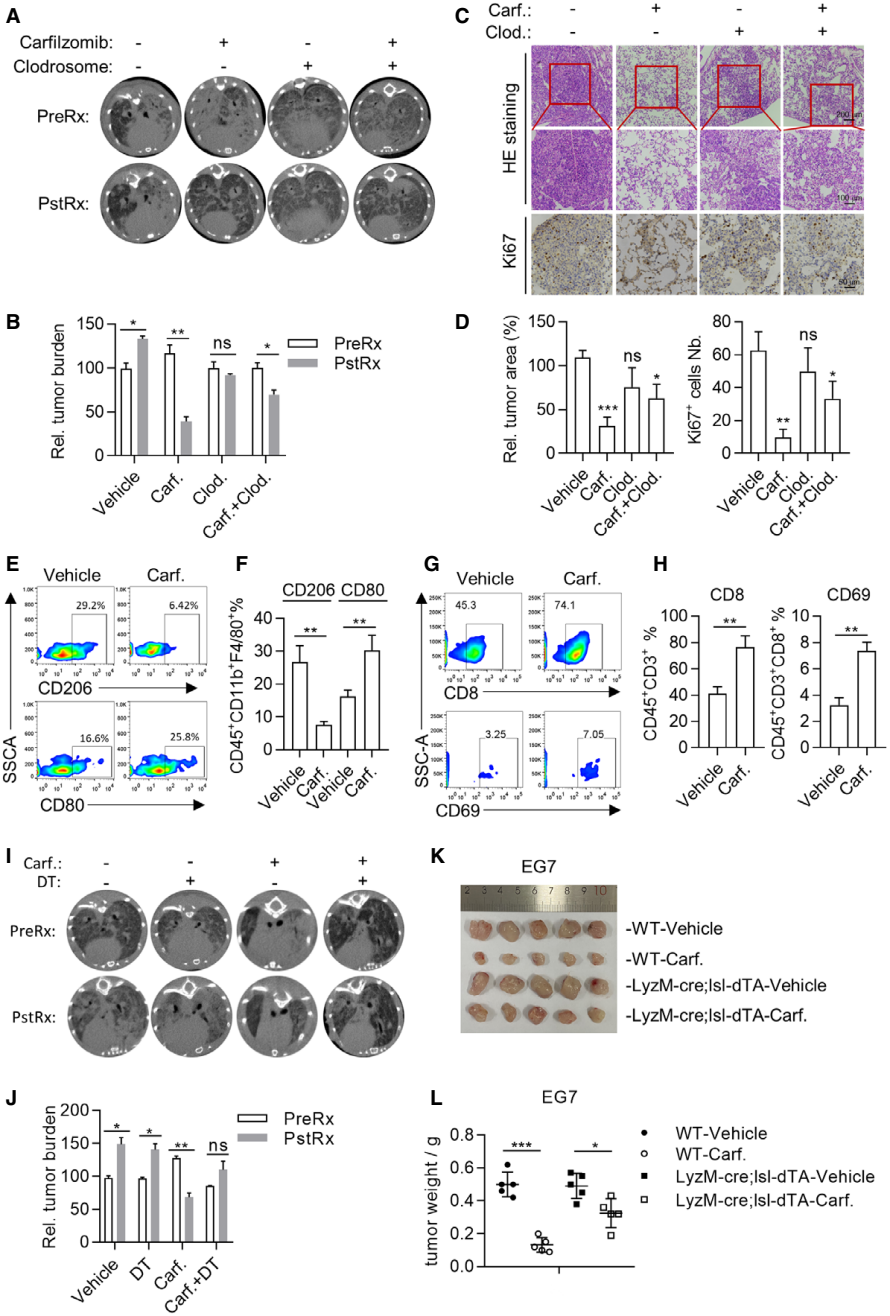
Carfilzomib shrinks tumor *in vivo* through promoting M2 macrophage polarization into M1‐like macrophage AMacrophages play a role in mediating shrinkage of lung cancer by Carfilzomib. TetO‐EGFR (*T*790M/*D*el19)/CC10‐rtTA (TD) mice were fed with doxycycline for 3 months to induce lung cancer. Tumor burdens were then documented through computed tomography (CT) imaging before treatment (PreRx). After 2‐week treatment with saline (iv.), Clodrosome (iv.), Carfilzomib (iv.), or Carfilzomib plus Clodrosome, mice were CT scanned again (PstRx). Treatment effect was determined by comparing tumor areas of PreRx and PstRx through ImagJ analysis.BStatistics of the tumor burden in lung tissue. Data are means ± SD of three independent experiments. *P < 0.05, **P < 0.01 (Student’s *t*‐test).CRepresentative images of Hematoxylin and eosin (H&E) staining of the lung tissue obtained from different treatment groups. Expression of Ki‐67 in lung tissues was detected by immunohistochemistry method.DStatistics of the tumor area and the number of Ki67‐positive cells in sections of lung tissue. Data are means ± SD of three independent experiments. *P < 0.05, **P < 0.01, ***P < 0.001 (Student’s *t*‐test).ECarfilzomib treatment promotes the infiltration of M1 macrophages. Tumor‐bearing TD mice were treated as indicated in (A) and euthanized. Lung tissues were dissected for evaluating M1/M2 populations through analysis of the expression of CD80 and CD206 by flow cytometry.FStatistics represent the proportion of CD80‐ or CD206‐positive cells in lung tissue. Data are means ± SD of three independent experiments. **P < 0.01 (Student’s *t*‐test).GCarfilzomib treatment promotes the infiltration and activation of CD8^+^ T cells. Lung tissues of (E) were used for evaluating the infiltration of CD8^+^ T cells and their expression of CD69 by flow cytometry.HStatistics about the proportion of CD8^+^‐ or CD69^+^‐positive cells in lung tissue. Data are means ± SD of three independent experiments. **P < 0.01 (Student’s *t*‐test).IGenetic confirmation of role played by macrophages in mediating Carfilzomib’s tumor shrinking effect through immune reconstitution with bone marrow from CD11B‐DTR mice. TD mice were irradiated with X‐ray (8 Gy for 20 min) and transplanted with 5 × 10^6^ of bone marrows from CD11B‐DTR mice. Immune‐reconstituted mice were induced to develop lung cancer by doxycycline diet. Mice were treated for 2 weeks with saline (iv.), DT (ip.), Carfilzomib (iv.), or Carfilzomib plus DT. Tumor burdens were documented through CT imaging before and after treatment.JStatistics of the tumor burden in lung tissues. Data are means ± SD of three independent experiments. *P < 0.05, **P < 0.01 (Student’s *t*‐test).K, LCarfilzomib shrinks tumor through modulating tumor microenvironment. EG7 cells were inoculated in wild‐type or LyzM‐cre;lsl‐dTA to allow tumor xenograft to reach a volume of around 80 mm^3^. Mice were randomized for treatment (*n* = 5) by saline or Carfilzomib for 2 weeks. The xenografts were dissected to photograph (K) or weigh (L) after treatment. Data are shown as mean ± SD and n indicates the number of biological replicates. *P < 0.05, ***P < 0.001 (Student’s *t*‐test). Source data are available online for this figure.

**Figure EV4:**
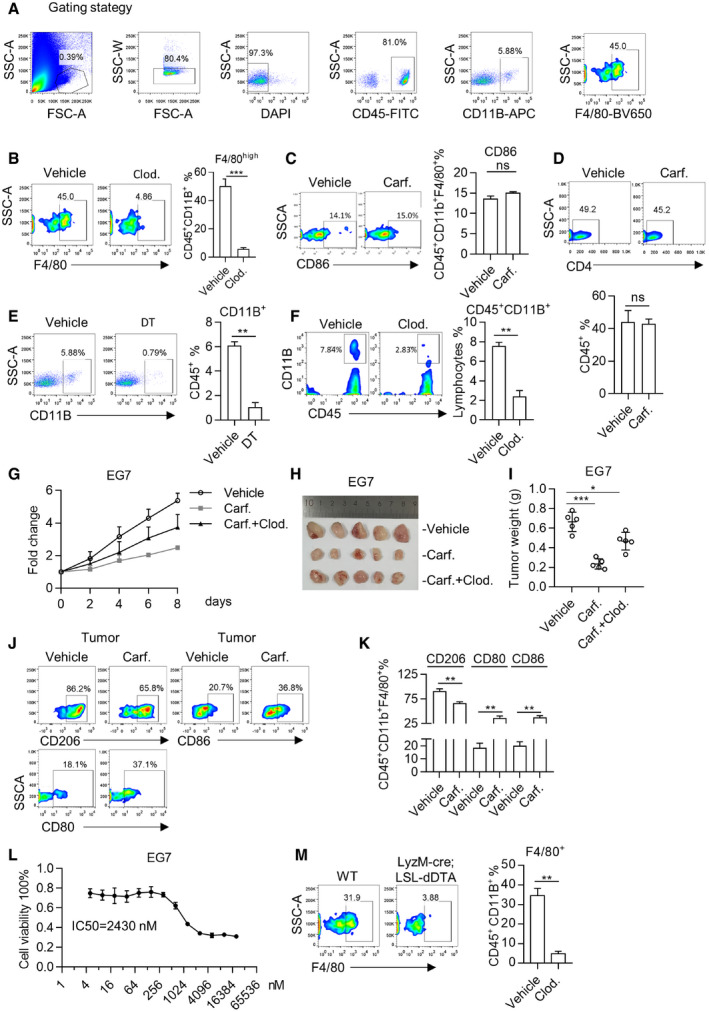
Carfilzomib shrinks tumor *in vivo* through promoting polarization of M2 macrophage into M1‐like macrophages AGating strategy for analyzing the macrophages in blood or lung tissues. After gating the singlets (by FSC‐A and SSC‐W) and living cells (DAPI negative), macrophages are gated on CD45^+^CD11b^+^ F4/80^high^ cells.BElimination of macrophages by Clodrosome. One day after injection of Clodrosome, orbital blood was collected, followed by red blood cells lysis. CD45^+^CD11b^+^ F4/80^high^ cells were analyzed by flow cytometry (left panel). Statistics represent the proportion of macrophages (right panel). Data are means ± SD of three independent experiments. ***P < 0.001 (Student’s *t*‐test).CCarfilzomib treatment has no remarkable effect on CD86 expression of infiltrating macrophages in tumor. Following protocol shown in Fig 5E, the expression of CD86 was detected by flow cytometry and the statistics represent the proportion of CD86. Data are means ± SD of three independent experiments.DImpact of Carfilzomib on infiltration of CD4^+^ T cells in lung cancer. Lung cancer‐bearing EGFR‐TD mice were treated for 2 weeks and then euthanized. Lung tissue was dissected for analysis of the infiltration of CD4^+^ T cells in tumors by flow cytometry (left panel). Statistics for proportion of CD4‐positive cells (right panel). Data are means ± SD of three independent experiments.EElimination of macrophages by DT in immune‐reconstituted TD mice. TD mice were irradiated with X‐ray (8 Gy for 20 min) and transplanted with 5 × 10^6^ of bone marrow cells from CD11b‐DTR mice. Immune‐reconstituted mice were fed with doxycycline diet for 12 weeks. Lung cancer was documented through CT. Mice were treated for 2 weeks with DT before FACS analysis. CD11B^+^ cells were analyzed by flow cytometry (left panel) and statistics on percentage of macrophages were calculated (right panel). Data are means ± SD of three independent experiments. **P < 0.01 (Student’s *t*‐test).FElimination of macrophages by Clodrosome. Clodronate liposomes of 200 μl were injected intravenously (iv.) every 3 days. Depletion of macrophages (CD45^+^CD11b^+^ cells) was analyzed in lung cancers (left panel). Statistics on percentage of macrophages are shown (right panel). Data are means ± SD of three independent experiments. **P < 0.01 (Student’s *t*‐test).G–IThe therapeutic effect of Carfilzomib is largely depending on the presence of macrophages in xenograft model. EG7 cells were inoculated in wild‐type C57BL/6 mice. Tumor volume was allowed to reach a volume of around 90 mm^3^. Mice were treated by saline, Carfilzomib, or Carfilzomib plus Clodrosome for 2 weeks (*n* = 5). Tumor growth was monitored during treatment (G) and the xenografts were dissected to photograph (H) or weigh (I) in the end of experiment. Data are shown as mean ± SD and *n* indicates the number of biological replicates. *P < 0.05, ***P < 0.001 (Student’s *t*‐test).JCarfilzomib treatment promotes the infiltration of M1 macrophages. After treatment, xenografts of (H) were harvested to analyze the expression of CD80, CD86, and CD206 by flow cytometry.KStatistics represent the proportion of CD80‐, CD86‐, or CD206‐positive cells in xenograft. Data are means ± SD of three independent experiments. **P < 0.01 (Student’s *t*‐test).LCytotoxicity of Carfilzomib on EG7 cells. EG7 cells were treated with Carfilzomib for 24 h at indicated concentration. Cell viability was analyzed with CCK8. IC50 (concentration for 50% of maximal effect) of Carfilzomib to repress EG7 cells growth was shown. Data are means ± SD of three independent experiments.MMacrophages were eliminated in LyzM‐cre; LSL‐dDTA mice. Orbital blood was collected and red blood cells were disrupted. Then, the CD45^+^CD11b^+^ F4/80^high^ cells were analyzed by flow cytometry (left panel). Statistics on percentage of macrophages were shown (right panel). Data are means ± SD of three independent experiments. **P < 0.01 (Student’s *t*‐test).

Carfilzomib has been approved for treatment of relapsed and/or refractory multiple myeloma (MM) in the Europe and United States. Given our observation of its ability to reprogram M2 into M1‐like macrophages, we hypothesized that Carfilzomib could rewire tumor microenvironment and contribute to shrink solid tumors. We, therefore, went further to test the ability of Carfilzomib to shrink tumors in our transgenic mouse models of autochthonous lung cancer driven by drug‐resistant EGFR mutant. We generated TetO‐EGFR (T790M/Del19)/CC10‐rtTA bitransgenic mice (designated TD mice) and induced lung adenocarcinoma after feeding them with doxycycline (Dox)‐containing diet following our earlier work (Zhou *et al*, 2009). Strikingly, computed tomography (CT) imaging revealed that Carfilzomib treatment led to significant regression of lung tumors (Fig 5A and B). Depleting macrophage with Clodrosome significantly impaired the tumor‐shrinking effect of Carfilzomib (Figs 5A and B, and EV4A and B), indicating that macrophages played an important role in mediating the treatment effect of Carfilzomib. Pathological analysis revealed high heterogeneity of nuclear morphology, ratio of nucleus to cytoplasm, and disordered arrangement of tumor cells along alveoli in these vehicle‐treated mice, indicative of malignant nature of these lung cancers. In striking contrast, we detected high degree of intra‐tumoral spaces and thicken alveolar walls in the lungs of Carfilzomib group, indicative of drastic remodeling of originally tumor‐occupied area. Moreover, heterogeneity of nuclear morphology and ratio of nuclear to cytoplasm in Carfilzomib‐treated mice are significantly milder (Fig 5C), pathologically confirming the therapeutic effects of Carfilzomib. Strikingly, these effects were severely compromised in Carfilzomib and Clodrosome combinationally treated mice (Fig 5C and D). Consistently, Ki67 staining showed that tumor growth inhibitory effect of Carfilzomib was compromised by Clodrosome co‐treatment (Fig 5C and D).

We further studied the impact of Carfilzomib on TAMs *in vivo* and found that Carfilzomib treatment drove TAMs to express higher level of CD80 but not CD86 (Figs 5E and F, and EV4C). Importantly, Carfilzomib treatment decreased the expression of CD206 on TAMs in TME (Fig 5E and F). Taken together, these results suggested that Carfilzomib reprogrammed TAMs into M1‐like macrophages *in vivo*.

Meanwhile, Carfilzomib treatments increased percentage of tumor‐infiltrating CD8^+^ T cells. These CD8^+^ T cells are activated since they are positive for CD69 expression (Fig 5G and H). Of note, we observed no significant difference in tumor‐infiltrating CD4^+^ T cells (Fig EV4D).

In order to further confirm that macrophages played a role mediating treatment effect of Carfilzomib on mutant EGFR‐driven lung cancers, we reconstituted the immune system of lethally irradiated TD mice with bone marrows of CD11B‐DTR transgenic mice, and then induced autochthonous lung cancers in these immune‐reconstituted mice by feeding them with Dox‐containing diet. Diphtheria toxin (DT) treatment effectively eliminated macrophages in these mice (Fig EV4E). Similar to Clodrosome, treatment with DT largely eliminated Carfilzomib treatment effect on lung cancers of TD mice (Fig 5I and J).

Based on our observation in lung cancer treatment, we hypothesized that besides cytotoxicity of Carfilzomib against myeloma cells, reprogramming macrophages also contributed to treatment effect of Carfilzomib on myeloma in clinic. We therefore chose to allograft E.G7‐OVA cells (designated EG7), an immunogenic mouse lymphoma cell line (Moore *et al*, 1988), which is relatively resistant to cytotoxicity of Carfilzomib (Fig EV4L) into mice subcutaneously, such that after treatment with Carfilzomib at clinically relevant concentrations, residual tumors could be detected and that contribution of macrophages to therapeutic effect can be analyzed. We inoculated EG7 cells in C57BL/6J mice and randomized the mice into three groups for treatment as indicated in Fig EV4G when tumors reached a volume of around 90 mm^3^ (*n* = 5 for each group). Interestingly, in this tumor model, depleting macrophages with Clodrosome largely eliminated the anti‐tumor effect by Carfilzomib (Fig EV4F–I). Likewise, FACS analysis suggested reprogramming of TAMs into M1‐like macrophages in this setting (Fig 4, EV4J and K). In order to further elucidate the role played by macrophages in mediating tumor‐shrinking effect, we crossed a transgenic mouse for conditional expression of DTA (rosa26‐lsl‐DTA) with LyzM‐cre (for expressing Cre in macrophages), such that bitransgenic offspring are deleted of macrophages (Fig EV4M). We confirmed that the ability of Carfilzomib to shrink EG7 allograft tumors was drastically compromised in this setting (Fig 5K and L).

Taken together, all these results suggested that reprogramming TAMs into M1‐like macrophages is an important mechanism mediating anti‐tumor effect of Carfilzomib *in vivo*.

### Carfilzomib synergized with PD‐1 inhibitors to treat lung cancer

**Figure 6:**
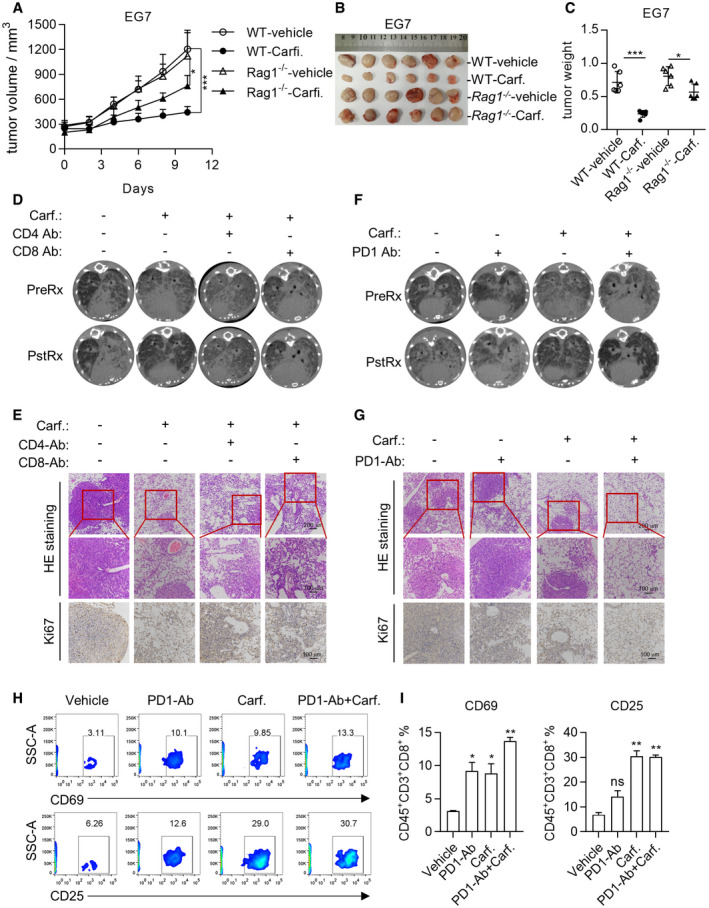
Carfilzomib synergizes with PD‐1 inhibitors to treat lung cancer A–CCarfilzomib shrinks tumor through modulating tumor microenvironment. EG7 cells were inoculated in wild‐type or *Rag1* ^−/−^ mice to allow tumor xenograft to reach a volume of around 80 mm^3^. Mice were randomized for treatment (*n* = 6) by saline or Carfilzomib for 2 weeks. Tumor growth was monitored (A) and the xenografts were dissected to photograph (B) or weigh (C) after treatment. Data are shown as mean ± SD and n indicates the number of biological replicates. *P < 0.05, ***P < 0.001 (Student’s *t*‐test).DCD4^+^ and CD8^+^ T cells play a role in mediating cancer‐shrinking effect of Carfilzomib. Lung cancer‐bearing TD mice were treated by saline, Carfilzomib, anti‐mouse CD4 antibody (ip.), anti‐mouse CD8 antibody (ip.), or Carfilzomib plus anti‐mouse CD4/8 antibody. CT scanning was performed after 2‐week treatment.ERepresentative images of H&E staining of the treated lung tissues. Expression of Ki‐67 in lung tissues was detected by immunohistochemistry method.FCarfilzomib synergized with PD1 antibody in shrinking lung cancers. Lung cancer‐bearing TD mice were treated by saline, anti‐PD1 antibody (ip), Carfilzomib, or Carfilzomib plus anti‐PD1 (ip). CT scanning was performed after 2‐week treatment.GRepresentative images of H&E staining of the lung tissues. Expression of Ki‐67 in lung tissue was detected by immunohistochemistry method.HCarfilzomib synergized with PD1 antibody in shrinking lung cancer through activating T cells. Treated TD mice were euthanized and the lung tissues were dissected to analyze the expression of CD69 and CD25 in CD8^+^ T cells by flow cytometry.IStatistics about the proportion of CD25‐ or CD69‐positive cells in CD8^+^ T cells. Data are means ± SD of three independent experiments. *P < 0.05, **P < 0.01 (Student’s *t*‐test). Source data are available online for this figure.

**Figure EV5:**
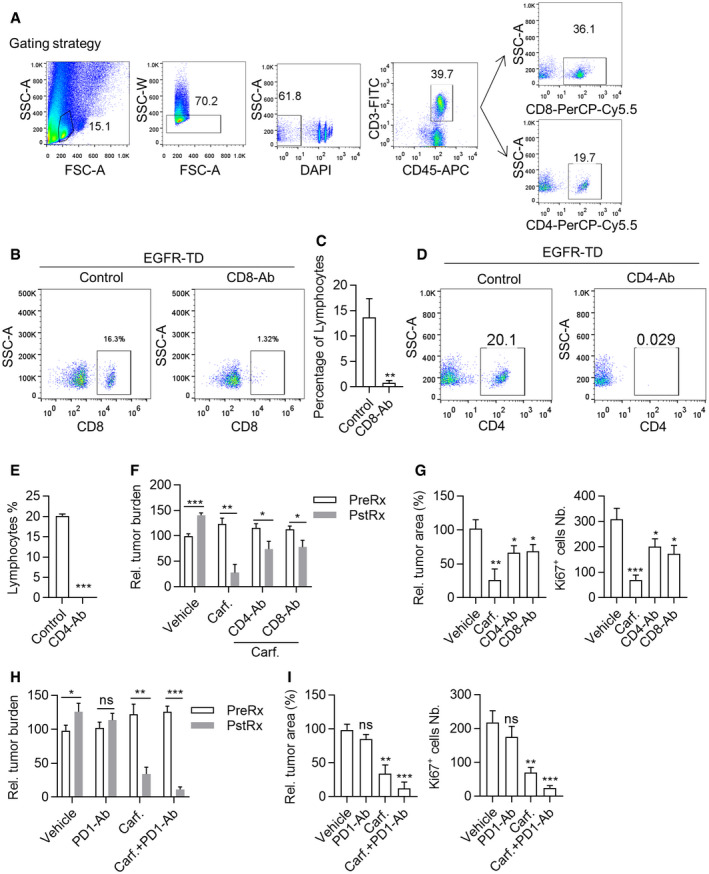
Carfilzomib synergizes with PD‐1 inhibitors to treat lung cancer AGating strategy was used for analyzing the infiltration of CD8^+^ or CD4^+^ T cells in lung tissues. After gating the single (by FSC‐A and SSC‐W) and living cells (DAPI negative), CD8‐ or CD4‐positive cells were gated on CD45^+^ cells.B, CElimination of CD8^+^ T cells by CD8α antibody. After 2‐week treatment with antibody, orbital blood was collected and red blood cells were lysed. CD8^+^ T cells were analyzed by flow cytometry (B). Statistics on percentages of CD8‐positive cells (C). Data are means ± SD of three independent experiments. **P < 0.01 (Student’s *t*‐test).D, EElimination of CD4^+^ T cells by CD4 antibody. After 2‐week treatment of antibody, orbital blood was collected and red blood cells were lysed. CD4^+^ T cells were analyzed by flow cytometry (D). Statistics on percentage of CD4‐positive cells (E). Data are means ± SD of three independent experiments. ***P < 0.001 (Student’s *t*‐test).F–IStatistics on tumor burden of mice before and after treatment. Data are means ± SD of three independent experiments (F, H). Statistics on relative tumor area and Ki67^+^ cells in sections of lung tissue of mice (G, I). Data are means ± SD of three independent experiments. *P < 0.05, **P < 0.01, ***P < 0.001 (Student’s *t*‐test).

As professional antigen‐presenting cells, macrophages are actively involved in educating T cells for anti‐tumor immunity. We found that the ability of Carfilzomib to shrink the EG7‐derived xenograft tumors was significantly compromised in RAG1^−/−^ mice, which produce no functional T cells (Fig 6A–C), suggesting critical involvement of T cells in this setting. To further confirm this result, we used antibodies to delete CD8^+^ (Fig EV5A–C) or CD4^+^ (Fig EV5D and E) T cells in our TD lung cancer transgenic mice and found that the therapeutic effect of Carfilzomib was significantly compromised after deletion of either population (Figs 6D and E, and EV5F and G).

PD‐1 inhibitors have been widely used to boost function of T cells for treating cancer patients. However, PD‐1 antibodies failed to benefit a significant portion of cancer patients. Driver mutation‐positive lung cancer patients, for example, did not respond to PD‐1 antibody treatment (Kato *et al*, 2017; Calles *et al*, 2020). We then tested combinational treatment with PD‐1 antibody and Carfilzomib on TD mice. Consistent with earlier reports, PD‐1 antibody singlet treatment showed no noticeable treatment effects on these EGFR mutant lung cancers (Fig 6F and G). Strikingly, we found that Carfilzomib synergized with PD‐1 antibody to almost completely regress (CR) lung cancers in TD mice (Figs 6F and G, and EV5H and I). We also found that combinational treatment with Carfilzomib and PD‐1 antibody resulted in more efficient activation of CD8 T^+^ cells (Fig 6H and I).

Taken together, our data showed that Carfilzomib synergized with PD‐1 antibody to shrink solid tumor.

### Proteasome inhibitors were capable of reprogramming human M2 macrophages into M1‐like macrophages

**Figure 7:**
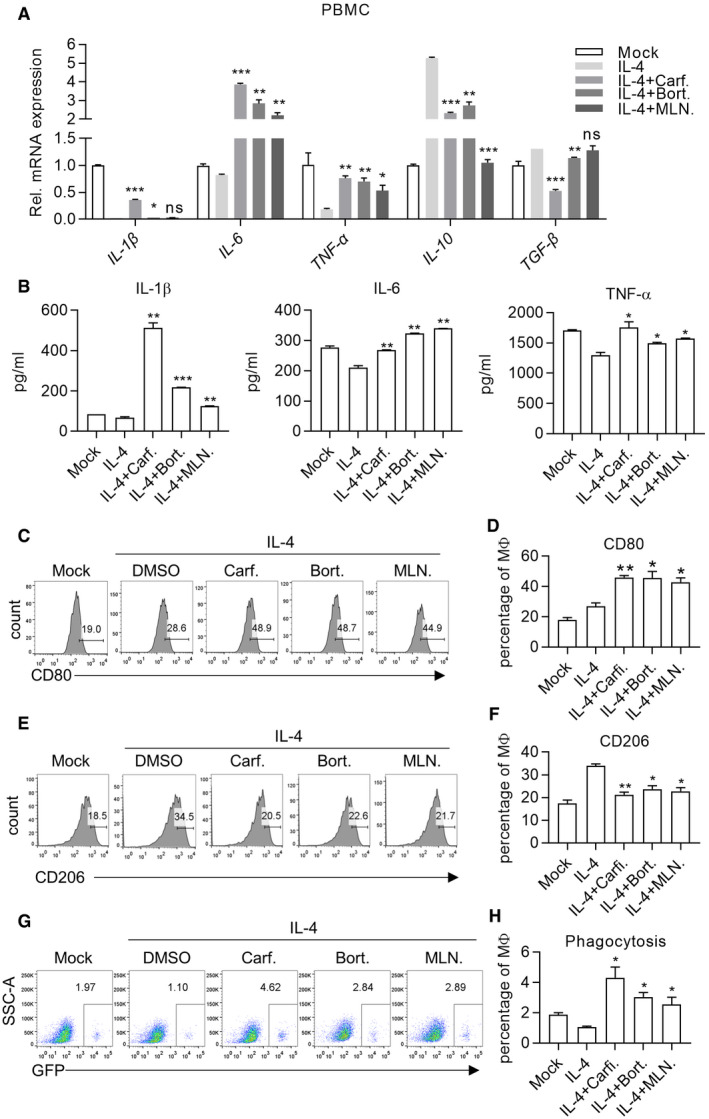
Proteasome inhibitors are capable of reprogramming human M2 macrophage into M1‐like macrophage ACarfilzomib, Bortezomib, and MLN9708 promote the expression of M1 macrophage markers (*Il‐1β, Il‐6, Tnfα*) and reduce the expression of M2 macrophage markers (*Il‐10, Tgfβ*) in macrophages derived from PBMCs. After induced by IL‐4 (20 ng/ml) for 24 h to differentiate into mature M2 macrophages, PBMCs‐derived macrophages were stimulated by Carfilzomib (1 μM), Bortezomib (1 μM), or MLN9708 (2 μM). RNA was extracted from cells 6 h after stimulation and the expression of mRNA was quantified through RT‐qPCR. Data from three experiments are presented as the mean ± SD. *T*‐test was used for statistical analysis of differences between groups. *P < 0.05, **P < 0.01, ***P < 0.001 (Student’s *t*‐test).BCarfilzomib, Bortezomib, and MLN9708 promote the secretion of inflammatory‐related cytokines in M2 macrophages derived from PBMCs. After induced by IL‐4 (20 ng/ml) for 24 h to differentiate into M2 macrophages, PBMCs‐derived macrophages were stimulated by Carfilzomib (500 nM), Bortezomib (500 nM), or MLN9708 (500 nM). The secretion of IL‐1β, IL‐6, and TNFα was detected through ELISA experiments 24 h after stimulation. Data from three experiments are presented as the mean ± SD. *T*‐test was used for statistical analysis of differences between groups. *P < 0.05, **P < 0.01, ***P < 0.001 (Student’s *t*‐test).C–FCarfilzomib, Bortezomib, and MLN9708 increase the expression of CD80 (C) and reduce the expression of CD206 (E) in M2 macrophages derived from PBMCs. After activated by IL‐4 (20 ng/ml) for 24 h to differentiate into mature M2 macrophages, PBMCs‐derived macrophages were stimulated by Carfilzomib (500 nM), Bortezomib (500 nM), or MLN9708 (500 nM). The representative histogram of CD80 and CD206 expression was analyzed by flow cytometry 12 h after stimulation. (D, F) Statistics represent the proportion of CD80‐ (D) or CD206‐ (F) positive cells in macrophages derived from PBMCs. Data from three experiments are presented as the mean ± SD. *T*‐test was used for statistical analysis of differences between groups. *P < 0.05, **P < 0.01 (Student’s *t*‐test).GCarfilzomib, Bortezomib, and MLN9708 enhance the phagocytic ability of macrophages derived from PBMCs. After induced by IL‐4 (20 ng/ml) for 24 h to differentiate into mature M2 macrophages, PBMCs‐derived macrophages were stimulated with Carfilzomib (500 nM), Bortezomib (500 nM), or MLN9708 (500 nM) for 12 h. The indicated drugs‐induced macrophages were starved for 2 h and incubated with L1210‐GFP cells as targets in serum‐free medium for another 2 h. Macrophages were thoroughly washed and stained with fluorochrome‐conjugated anti‐CD11B antibody and analyzed through flow cytometry. Phagocytosis efficiency was determined as the percentage of GFP‐positive cells in CD11B^+^ population.HStatistics represent the percentage of phagocytes and data are means ± SD of three independent experiments. *P < 0.05 (Student’s *t*‐test).

We next checked the ability of PIs to reprogram human macrophages. We found that administration with Carfilzomib, Bortezomib, or MLN9708 upregulated expression of *IL‐1β*, *IL‐6,* and *TNFα* and inhibited expression of *IL‐10* and *TGFβ* in IL4‐activated M2 macrophages (Fig 7A). Upregulation of protein level of IL‐1β, IL‐6, and TNFα was confirmed through ELISA assay (Fig 7B). We also found that these PIs upregulated CD80 expression and downregulated CD206 expression (Fig 7C–F and Appendix Fig S3) in M2 macrophages. Consistently, treatment with these PIs enhanced ability of M2 cells to phagocytose L1210‐GFP tumor cells (Fig 7G and H).

Taken together, our data showed that FDA‐approved PIs (Carfilzomib, Bortezomib, or MLN9708) were able to reprogram alternatively activated macrophages into M1‐like macrophages.

## Discussion

Immunotherapeutics as exemplified by PD‐1 inhibitors yielded unprecedented efficacies and durable responses across various types of advanced‐stage cancers. Unfortunately, a significant portion of patients showed no response to this type of treatment. Urgent needs remain in clinic to develop synergizers for expanding the usage of PD‐1 inhibitor. In our current work, we show that Carfilzomib, and other proteasomal inhibitors to a lesser degree, reprogrammed M2 into M1‐like macrophages, such that more CD8^+^ T cells infiltrated and controlled tumor *in vivo*. We have shown that proteasomal inhibition activated IRE1α to recruit TRAF2 and activate NF‐κB to transcribe inflammatory cytokine and chemokine, thus creating a favorable tumor microenvironment for immunotherapy. More importantly, Carfilzomib synergized with PD‐1 antibody to shrink lung cancers driven by mutant EGFR, which is otherwise not responsive to PD‐1 antibodies.

Macrophages are flexible in terms of gene expression and differentiation. They behave as plastic cells modifying transcription profile along a continuous spectrum, with M1 and M2 phenotypes as two extremes (Ruytinx *et al*, 2018). We have shown that Carfilzomib upregulated M1 biomarkers and downregulated M2 biomarkers in IL‐4‐activated macrophages in most cases. However, we did not exclude the possibility that Carfilzomib reprogrammed macrophages will end up at intermediate stages between M1 and M2 under some circumstances. For instance, we noticed that Carfilzomib treatment fail to upregulate TNFα in IL‐4‐activated RAW264.7 cells. Likewise, proteasomal inhibition through shRNA‐mediated knockdown of Psmb5 failed to downregulate CD206 and Arg‐1 in these cells. Carfilzomib upregulate M1 biomarkers in M2 macrophages in a direct manner because IRE1α‐TRAF2 activates NF‐κB activity. We guess that the effect of Carfilzomib on CD206 and Arg1 is indirect, most probably because Carfilzomib can induce M1 polarization. Indeed, impact of M1 polarization on the expression of M2‐related genes has been reported. The negative regulation of M2 genes by M1 polarizing signals is relatively easier to understand. For example, Btk activated by LPS inhibits M2 genes by macrophages (Ni Gabhann *et al*, 2014). NO produced during M1 polarization inhibits macrophages to transcribe IL‐10 gene (Kaneko *et al*, 2003). On the contrary, M1 polarization can feedback to upregulate M2‐related gene in macrophages. For example: M1 macrophages feature activated glycolysis (Jha *et al*, 2015); lactic acid, the intermediate metabolite of glycolysis, has been found to promote expression of M2‐related genes (Feng *et al*, 2018). Therefore, the impact of Carfilzomib on expression of M2‐related genes (like CD206 and Arg1) is indirect and more environment dependent. The actual environment where the reprogrammed macrophage cell sits may thus have important impact on whether to downregulate M2 biomarkers by Carfilzomib. Consistent with this hypothesis, inhibiting IRE1α with Kira6 (inhibiting IRE1 kinase activities) but not 4μ8C (inhibiting IRE1 RNase activities) inhibited M1 marker genes in M2 macrophages.

In our study, we found that IRE1a knockout severely inhibited expression of IL‐1B and IL‐6 in BMDM and that this effect was much milder in RAW264.7 cells. The mechanisms underlying different sensitivity of BMDM and RAW264.7 could be complex. Several different mechanisms have been reported to control IL‐1B transcription, including transcription factors like Spi‐1/PU.1 (Kominato *et al*, 1995), NF‐κB plus C/EBPβ, and HIF‐1α plus C/EBPβ (Pulugulla *et al*, 2016), and epigenetic modifications of its promoter region (Hashimoto *et al*, 2009). The RAW264.7 cell line was established from a tumor induced by the Abelson murine leukemia virus (Ralph & Nakoinz, 1977), which was different from BMDM. Therefore, knockout of IRE1a resulted in different expression of IL‐1B and IL‐6 in RAW264.7 and BMDM, which could be reasonably possible.

Carfilzomib has been approved for treating MM patients. Previous work has shown that this drug is highly cytotoxic to multiple myeloma tumor cells. Our current work suggests that this drug could reprogram TAM into M1‐like macrophages, thus rewiring tumor microenvironment. We speculate that this mechanism may contribute to the therapeutic effect of Carfilzomib on MM patients. Of note, MG132 has been noticed to prolong half‐life of IL‐1β precursor (Moors & Mizel, 2000).

Genome‐wide screening to systemically identify gene targets for reprogramming M2 into M1 will undoubtedly deepen our understanding of M1 differentiation. More importantly, this type of work will yield realistic gene target for developing drugs for manipulation of TAMs. Our current work showed that M2 macrophages derived from bone marrow cells of IL1β‐luciferase transgenic mice represent an ideal model for genome‐wide screening to identify gene targets for this purpose. During preparation of this manuscript, a phenotypic screening based on expression of cell surface M1 markers (CD80 and CD86) and M2 markers (CD206 and CD163) through staining with fluorescence‐labeled antibodies was reported to identify chemicals capable of reprograming M2 into M1‐like macrophages (Hu *et al*, 2021). This study included 760 FDA compounds in their chemical library. However, Carfilzomib and MLN9708 were not highlighted in their screening. Moreover, Bortezomib was highlighted as M2 inducer (Ubels *et al*, 2018). In striking contrast, all three proteasomal inhibitors were highlighted in our screening and were functionally confirmed in our current study. Our current work, therefore, highlights the robustness of BMDMs from IL1β‐luciferase transgenic mice for high‐throughput screening M1‐like macrophage inducers.

Numerous PD‐1 inhibitory antibodies have been approved for clinical use around the world, with many more in various stages of clinical trial. However, majority of lung cancer patients fail to benefit from these inhibitors. The critical issue with PD‐1 inhibitor therapy is limited T cells infiltration into tumor foci. Fu lab showed that facilitating T cells infiltration in tumor microenvironment overcame resistance to PD‐1 blockade (Tang *et al*, 2016). TAMs contribute partly to deter T cells from infiltrating into tumor foci by secreting anti‐inflammatory cytokines/chemokines. Transforming TAMs toward tumoricidal inflammatory immune cells is, therefore, an attractive strategy. Indeed, early preclinical data showed that reprogramming TAM into M1 dramatically regressed established tumors (Pyonteck *et al*, 2013). To date, several functionally important proteins have been successfully proved as drug target capable of reprogramming TAM into M1 subtype, including PI3Kγ, HDAC9, and CFEV1R (Pyonteck *et al*, 2013; Kaneda *et al*, 2016; Guerriero *et al*, 2017). Our current work showed that targeting proteasomal function represented another efficient way to transform M2/TAM into M1‐like macrophages. This could result in transforming tumor microenvironment from immune suppressive into anti‐tumor inflammatory. We have shown that Carfilzomib treatment facilitates recruitment of CD8^+^ T cells into tumor loci, thus synergized with PD‐1 inhibitors to shrink solid tumors in transgenic mouse model of autochthonous lung cancer.

Our *in vitro* results indicated that peptide‐loaded Ci‐M1 cells enhance proliferation of both CD4^+^ and CD8^+^ T cells. However, the amount of CD4^+^ T cells in Carfilzomib‐treated lung tumors was similar to those of vehicle‐treated tumors. We guess that the discrepancy between *in vitro* and *in vivo* data could be explained by the complex process involved in maintaining the homeostasis *in vivo*. Several factors could affect apparent number of CD4^+^ T cells in tumor: recruitment (CXCL9/10), proliferation (IL‐15), and death (IL‐2) (Waldmann, 2002; Croudace *et al*, 2012). If the microenvironment of Carfilzomib‐treated lung cancers releases less chemoattractant, fewer CD4^+^ T cells are recruited into the tumor locus. However, the faster proliferation of CD4^+^ T cells could compensate for the lower amount of recruited seed CD4^+^ T cells. Or it could be that CD4^+^ T cells underwent faster apoptosis after proliferation.

Given the safety profile shown in clinic for Carfilzomib, our work highlights potential clinical application of combinational treatment of Carfilzomib and PD‐1 inhibitors for treating patients with solid tumors.

## Materials and Methods

### Mice

*Il‐1β* **‐**luciferase mice (#NM‐CKO‐00080, Shanghai Model Organisms Co ltd), transgenic mice ERN1^fl/fl^ (#NM‐CKO‐00077, Shanghai Model Organisms Co ltd), wild‐type C57BL/6 (CD45.2), LyzM‐Cre (#004781, Jax), rosa26‐LSL‐dTA (#009669, Jax), CD11B‐DTR(#006000, Jax), CD45.1(#002014, Jax), Rag1^−/−^ (# 002216, Jax), OT‐I (#003831, Jax), and OT‐II (#004194, Jax) TCR transgenic mice and TetO‐EGFR (*T*790M/*D*el19)/CC10‐rtTA transgenic mice were maintained on C57BL/6J background and kept in Laboratory Animal Science Institute, JINAN University. ERN1^fl/fl^ mice were crossed with LyzM‐Cre mice to produce bitransgenic mice with macrophages deficient of *ERN1*. Rosa26‐LSL‐dTA mice were crossed with LyzM‐Cre mice for deleting macrophages in bitransgenic offspring. Age‐ and sex‐matched mice were used in our experiments. All mice were housed in specific pathogen‐free conditions. All animal procedures were conducted in strict accordance with guidelines for the care and use of laboratory approved by the Institute of Laboratory Animal Science Institute, Jinan University.

### Reagents

Carfilzomib (T1795), Bortezomib (T2399), and MLN9708 (T2016) were purchased from TargetMol; Kira6 (S8658), 4μ8c (S7272), GSK2606414 (S7307), and MG132 (S2619) were purchased from Selleck; Tunicamycin (HY‐A0098) and Thapsigargin (HY‐13433) were purchased from MCE; 4‐Phenylbutyric acid (P21005), Diphtheria Toxin (D0564), Ovalbumin_257‐264_ (S7951), and Ovalbumin_323‐339_ (O1641) peptide were purchased from Sigma‐Aldrich; Ficoll‐Paque™ PLUS (17‐1440‐02) was purchased from GE Healthcare; fetal bovine serum (FBS, 42G9274K), penicillin/streptomycin (P/S, 15140122), and Tripsin were purchased from Gibco.

### Cell lines and culture

Murine fibroblasts cell line L929, murine colon cancer cell line MC38, murine macrophage cell line Raw264.7, and T lymphoma cell lines EG7 and 293T were from ATCC. Murine leukemic cell line L1210 was kindly gifted by Dr. Jun Chen (Sun Yat‐Sen University, Guangzhou, China). All cells were cultured at 37°C with 5% CO_2_.

### Luciferase assay

BMDMs from *Il‐1β* ***‐*** *luciferase* transgenic mice (BMDM‐ *Il‐1β*) were seeded into Costar Assay 96‐well plate (3903, CORNING) overnight. After inducing by IL‐4 for 24 h, different drugs were added to stimulate BMDMs for another 12 h. Bright‐GloTM Luciferase Assay System (E2650, Promega) was then applied to detect the expression of *Il‐1β* according to luciferin value.

### RNA extraction and quantitative RT‐qPCR

Total RNA was extracted from macrophages by trizol (15596018, ambion) and around 1 μg RNA was reverse transcribed into cDNA through AccuRT Genomic DNA Removal Kit (G492, Applied Biological Materials). Then, the cDNA was amplificated by MonAmp^TM^ Fast SYBR Green qPCR Mix (110344, Monad) through Bio‐Rad Real‐Time PCR System. The expression levels of mRNA were normalized by *Gapdh* or *β‐ACTIN*. The following sequences are qPCR primers used in our experiments.

*Gapdh* qPCR forward primer: ACGGCCGCATCTTCTTGTGCA
*Gapdh* qPCR reverse primer: ACGGCCAAATCCGTTCACACC
*Il‐1β* qPCR forward primer: GCAACTGTTCCTGAACTCAACT
*Il‐1β* qPCR reverse primer: ATCTTTTGGGGTCCGTCCAACT
*Il‐6* qPCR forward primer: TCTGCAAGAGACTTCCATCCAGTTGC
*Il‐6* qPCR reverse primer: AGCCTCCGACTTGTGAAGTGGT
*Inos* qPCR forward primer: GAGACAGGGAAGTCTGAAGCAC
*Inos* qPCR reverse primer: CCAGCAGTAGTTGCTCCTCTTC
*Arg‐1* qPCR forward primer: CATTGGCTTGCGAGACGTAGAC
*Arg‐1* qPCR reverse primer: GCTGAAGGTCTCTTCCATCACC
*Cd206* qPCR forward primer: CTCTGTTCAGCTATTGGACGC
*Cd206* qPCR reverse primer: CGGAATTTCTGGGATTCAGCTTC
*Bip* qPCR forward primer: TTCAGCCAATTATCAGCAAACTCT
*Bip* qPCR reverse primer: TTTTCTGATGTATCCTCTTCACCAGT
*Chop* qPCR forward primer: CCACCACACCTGAAAGCAGAA
*Chop* qPCR reverse primer: AGGTGAAAGGCAGGGACTCA
*sXbp1*qPCR forward primer: CTGAGTCCGAATCAGGTGCAG
*sXbp1* qPCR reverse primer: GTCCATGGGAAGATGTTCTGG
*unXbp1* qPCR forward primer: CAGCACTCAGACTATGTGCA
*unXbp1* qPCR reverse primer: GTCCATGGGAAGATGTTCTGG
*Psmb5* qPCR forward primer: CTGTGGCTGGGATAAGAGAGGC
*Psmb5* qPCR reverse primer: CAGGTCATAGGAGTAGCCTCGA
*Perk* qPCR forward primer: CCGATGTCAGTGACAACAGCTG
*Perk* qPCR reverse primer: AAGACAACGCCAAAGCCACCAC
*Atf6* qPCR forward primer: TCGCCTTTTAGTCCGGTTCTT
*Atf6* qPCR reverse primer: GGCTCCATAGGTCTGACTCC
*ACTIN* qPCR forward primer: ACGTGGACATCCGCAAAG
*ACTIN* qPCR reverse primer: GACTCGTCATACTCCTGCTTG
*IL‐1β* qPCR forward primer: ATGATGGCTTATTACAGTGGCAA
*IL‐1β* qPCR reverse primer: GTCGGAGATTCGTAGCTGGA
*IL‐6* qPCR forward primer: ACTCACCTCTTCAGAACGAATTG
*IL‐6* qPCR reverse primer: CCATCTTTGGAAGGTTCAGGTTG
*TNFα* qPCR forward primer: CCCCAAAGGGATGAGAAGTT
*TNFα* qPCR reverse primer: CACTTGGTGGTTTGCTACGA
*IL‐10* qPCR forward primer: GACTTTAAGGGTTACCTGGGTTG
*IL‐10* qPCR reverse primer: TCACATGCGCCTTGATGTCTG
*TGFβ* qPCR forward primer: CTAATGGTGGAAACCCACAACG
*TGFβ* qPCR reverse primer: TATCGCCAGGAATTGTTGCTG

### Enzyme‐linked immunosorbent assay (ELISA)

ELISA was carried out according to the manufacturer’s instructions. Mouse IL‐1β Valukine ELISA (VAL601), Mouse IL‐6 Valukine ELISA (VAL604), Mouse TNF‐*α* Valukine ELISA (VAL609), Human IL‐1β Valukine ELISA (VAL101), Human IL‐6 Valukine ELISA (VAL102), and Human TNF‐α Valukine ELISA (VAL105) were purchased from Novus Biologicals.

### Flow cytometry and antibodies

For the detection of cell surface protein, the xenograft and lung tissues were isolated and grinded on the mesh 200. We collected the cells suspension and used the lysis solution (#NH4CL2009, TBD) to disrupt red blood cells. Cells were then filtered with strainers to prepare single‐cell suspension. Besides, macrophages derived from BMs or PBMCs were also prepared as single‐cell suspension. Then, cells stained with the following fluorochrome‐conjugated antibodies in PBS containing 1% BSA on ices for 15–20 min according to dilution ratio recommended by manufacturers: anti‐mouse CD45, anti‐mouse CD45.2, anti‐mouse CD45.1, anti‐mouse CD11B, anti‐mouse F4/80, anti‐mouse CD80, anti‐mouse CD86, anti‐mouse CD206, anti‐mouse CD3e, anti‐mouse CD8a, anti‐mouse CD4, anti‐mouse CD69, anti‐mouse CD25, anti‐mouse H‐2K^b^‐SIINFEKL, anti‐human CD45, anti‐human CD11B, anti‐human CD68, anti‐human CD80, and anti‐human CD206 were used in flow cytometry; all the FACS antibodies’ details were shown in Appendix Table S1. At the same time, DAPI were used to label dead cells as well as gating the living cells in analysis. Analysis was performed on BD celesta and gating strategies were shown in the Figure EV data.

### Lentivirus production and transduction

Recombinant lentiviruses were generated by co‐transfecting 293T cells with plasmids encoding genes of interest and packing plasmids. To generate L1210 cell line stably overexpressing GFP (L1210‐GFP), lentivirus was produced by transfection of 293T cells with pTRIP‐GFP, infections were performed using 8 μg/ml polybrene (Sigma #107689, Sigma). L1210‐GFP monoclones were selected through limiting dilution method. For the generation of *Ern1* knockout cell lines by CRISPR/Cas9 technology, mouse *Ern1* guide RNA sequence was used: 5′‐ ACCACCGTATCTCAGGATGT −3′. For the generation of mouse *Psmb5/Atf6/Perk*‐knockdown cell Lines, shRNA target sequence for mouse mRNA is as follows:

5′‐ CGAATCTATGAGCTTCGCAAT −3′ (sh*Psmb5*);
5′‐ GCTGTCTGTGTGATGATAGTA −3′ (sh*Atf6*);
5′‐ CCATGAGTTCATCTGGAACAA −3′ (sh*Perk*).

Oligos of sgRNA or shRNA were annealed and ligated into lenti‐CRISPR‐v2 or pLKO vector, and then the products of ligation were used to transform *E*. *coli*. Recombinant lenti‐sg*Ern1* or pLKO‐sh*Psmb5/*sh*Atf6/*sh*Perk* plasmids were transfected into 293T cells by using VigoFect (T001, Vigorous) for packaging recombinant lentiviruses. Raw264.7 cells were infected with the lentivirus and selected by puromycin to acquire stable KO/KD monoclonal cell line. Lenti‐sgNontarget or pLKO‐shGFP plasmids were conducted as control in the study.

### Western blot (WB) and immunoprecipitation (IP)

The cells were harvested (10^6^ cells for WB and 10^7^ cells for endogenous IP) and then lysed with NP‐40 lysis buffer on ice for around 30 min. For each IP, WCL incubated with indicated antibodies and protein A/G PLUS‐Agarose (sc‐2003, Santa Cruz) for around 3 h at 4°C. Finally, the protein was subjected for SDS‐PAGE and incubated with antibodies overnight at 4°C. The following antibodies were used in immunoprecipitations and western blots, including Anti‐β‐Actin, anti‐IRE1α, anti‐p‐IRE1α, anti‐IκBα, anti‐p‐IκBα, anti‐P65, anti‐p‐P65, anti‐TRAF2; all antibodies’ details are shown in Appendix Table S1. Next day, after washing with TBST (TBS containing Tween‐20) and staining with HRP‐conjugated secondary antibodies, membranes were washed again and imagined by using Chemiluminescent HRP Substrate (WBKLS0500, Millipore) to present protein.

### Hematoxylin and eosin (H&E) staining immunohistochemistry analysis

After euthanasia, the xenograft or lung tissues were dissected from mice and fixed in 10% neutral buffered formalin solution (HT501320; Sigma‐Aldrich) overnight. The tissues were dehydrated, paraffin embedded, and sectioned for staining with hematoxylin and eosin or anti‐Ki67 (Rb mAb, ab15580, Abcam) for immunohistochemistry.

### CD4^+^ and CD8^+^ T‐cell proliferation

Splenocytes were gained from OT‐I or OT‐II TCR transgenic mice and prepared as single‐cell suspension. Then, stained with 5 μM CFSE in dark at room temperature for 5–10 min. BMDMs in different treatment groups were transfected with 10 μ g/ml of OVA_257‐264_ peptide or OVA_323‐339_ peptide for 1 h. BMDMs were then washed twice and co‐incubated with CFSE‐labeled OT‐I or OT‐II cells, respectively, with 10 ng/ml IL‐2 (PeproTech) added in completed RPMI‐1640 medium. After 72 h, OT‐I or OT‐II cells were stained with anti‐CD8 or anti‐CD4 fluorochrome‐conjugated antibodies. Cell division was then determined by dilution of CFSE through flow cytometry. Splenocytes were obtained from wild‐type B6 mice, induced by plate‐bound anti‐Mo CD3 (2 μg/ml, 16‐0031‐85, eBioscience^TM^) in 96 wells culture plate with the presence of anti‐Mo CD28 (1 μg/ml 16‐0281‐82, eBioscience™) in complete PRMI 1640 medium (Gibco).

### *In vitro* phagocytosis assays

1 × 10^5^ macrophages were seeded in a 12‐well tissue culture plate and induced with IL‐4 for 24 h. Proteasomal inhibitors were then added to activate macrophages for 12 h. Next, changing with serum‐free medium to starve macrophages for 2 h. During this period, the macrophages were stained with fluorescent dyes CellMask^TM^ Deep Red plasma (1:1,000, REF C10046, Invitrogen) for 20 min at 37°C, followed by washing with PBS for three times. The target cells L1210‐GFP were collected and washed with PBS, then 5 × 10^5^ L1210‐GFP were added in 12‐well culture plate to incubate with macrophages. After incubation for 2 h, the suspended target cells were washed out with PBS. Phagocytosis of macrophages was observed under fluorescence microscope. On the other hand, flow cytometry was used to detect the proportion of CD11B‐ and GFP‐positive cells which were phagocytic macrophages.

### Immunofluorescence

Raw264.7 cells were seeded in confocal dish and induced by IL‐4 for 24 h, then treated with DMSO or drugs for another 12 h. The cells were fixed with 4% paraformaldehyde solution for 15 min and permeabilizated with 0.5% Triton X‐100 solution for 10 min. After blocking with 1% BSA in PBS for 30 min, cells were incubated with anti‐IRE1α in 1% BSA overnight at 4°C. Fixed cells were washed and stained with fluorochrome‐conjugated secondary antibody for 1 h at room temperature, then nucleus were stained with 2 μg/ml DAPI for 1 min and the redundant DAPI was washed out. After sealing, fluorescence photography could be carried out by laser confocal.

### Preparation of tumor culture supernatant (TSN) for derivation of TAMs

5 × 10^6^ L1210 or EG7 cells were seeded in 10 cm dish containing 10 ml of complete DMEM or RPMI‐1640 medium (10%FBS + 1% P/S), respectively, for 2–3 days. TSN was collected and centrifuged at 450 RCF/min for 8 min to remove cells, and then centrifuged at 3000 RCF/min for 8 min to remove debris. Supernatant was stored immediately at −80°C before use. For domestication experiment, BMDMs were cultured in complete RPMI‐1640 medium with 30% TSN of L1210 or EG7 cells for 36 h.

### Depletion of macrophages *in vivo*

For Clodronate liposomes‐induced macrophages depletion, 200 μl Clodronate liposomes (CLD‐8909, FormuMax Scientific USA) were given by intravenous (iv.) injection every 3 days during Carfilzomib treatment. One day after injection of liposomes, macrophages were analyzed by FACS.

For immune system reconstitution, mice were lethally irradiated with X‐ray (8 Gy for 20 min) and transplanted with bone marrow of CD11B‐DTR transgenic mice. Reconstituted mice were administered with 20 μg/kg diphtheria toxin (DT) intraperitoneally to remove myeloid‐derived macrophages. DT was injected three times a week to ensure elimination of macrophages during treatment. The clearance efficiency of macrophages was evaluated by flow cytometry. Gating strategies are shown in Figure EV data.

### Depletion of CD4^+^ and CD8^+^ T cells *in vivo*

Anti‐mouse CD8α (BE0081, Bio X Cell) or anti‐mouse CD4 (BP0003‐3, GK1.5, Bio X Cell) of 10 mg/kg was used to deplete CD8^+^ or CD4^+^ T cell, respectively, *in vivo* through intraperitoneal injection. Antibodies were injected twice a week to ensure low level of T cells during the treatment. The clearance efficiency of T cells was evaluated by flow cytometry. Gating strategies are shown in Figure EV data.

### Xenograft model *in vivo*

Tumor cells were resuspended in mixture of equal volume of Matrigel (356237, CORNING) and PBS. Eight‐week wild‐type C57BL/6 or Rag1^−/−^ mice were inoculated with 0.5 million EG7 cells subcutaneously on right flank. Tumors were allowed to grow to a volume of around 90 mm^3^. Mice were then randomly grouped for treatment. Carfilzomib of 3 mg/kg was administered via intravenous injection, with saline as vehicle. Tumors were monitored every 2 days. In the end of treatment, the mice were euthanized and the xenografts were dissected, weighed and photographed. In addition, the xenograft could be used for flow cytometry after grinding.

### Macrophage preparation and polarization

For bone marrow‐derived macrophages (BMDMs), bone marrows from 8‐week wild‐type mice in C57BL/6 background were cultured with complete RPMI‐1640 medium containing 20–30% (v/v) supernatant of L929 cells for 4–5 days to differentiate into mature macrophages.

For murine macrophage polarization, Raw264.7 cells or BMDMs were plated in six‐well plates prior to treatments. Then, the cells were treated with LPS alone (100 ng/ml) or IL‐4 (20 ng/ml, #214‐14, PeproTech) for 24 h.

For human macrophage polarization, PBMCs were isolated with Ficoll through gradient centrifugation from healthy donors according to manufacturer’s instruction. Purified PBMCs were seeded into culture plates and washed with PBS to remove the non‐adherent cells after 2 h. The adherent cells were cultured with human M‐CSF (50 ng/ml, #300‐25, PeproTech) in complete DMEM medium for 6 days to fully differentiate macrophages, followed by treatment with 20 ng/ml rhIL‐4 (204‐IL/CF, R&D) to induce M0 macrophages into mature M2 macrophages for 1 day. These cells were used for subsequent treatments. Protocol was approved by Ethnic Committee of Jinan University. Informed consent was obtained from all subjects, and the experiments were conformed to the principles set out in the WMA Declaration of Helsinki and the Department of Health and Human Services Belmont Report.

For inhibitors studies, M2 macrophages were induced by IL‐4 for 24 h, followed by pretreatment with inhibitors (Kira6, 4μ8C, GSK2606414, and 4‐PBA) for 1 h. These cells were incubated with Carfilzomib, Bortezomib, and MLN9708 for further experiments.

### Tumor model *in vivo*

One‐month‐old TetO‐EGFR T790M/Del19; CC10rtTA bitransgenic mice (designated TD mice) were fed with doxycycline‐containing diet for around 12 weeks to induce lung cancer. Tumor burdens in lungs were monitored by computed tomography (CT, SNC‐100, PINSENO HEALTHCARE). After confirming, lung cancer‐bearing mice were randomly divided into groups for treatment with Carfilzomib (3 mg/kg (iv.) three times a week) and anti‐mouse PD1 antibody (10 mg/kg (ip.) every 2 days, BioXcell, BP0033‐2), respectively. After 2 weeks of treatment, CT was performed again to evaluate the therapeutic effect compared with the scalogram before treatment. Mice were then euthanized. The lung tissues were fixed to embed for pathological analysis. Parallelly, tumor nodules were grinded for flow cytometry analysis.

### Statistical analysis

Date statistics were analyzed by GraphPad Prism 7.0 software and the differences among the groups were compared by Student’s unpaired *t*‐test. Results are presented as means ± SD and the *P* < 0.05 indicated statistical significance.

## Author contribution

LC conceived the work, designed experiments, analyzed data, and wrote manuscript. QZ designed experiments, analyzed data, and wrote manuscript. QZ, JLia, TY, JLiu, BL, YL, ZF, ZL, QX, YH, WC, and MX performed experiments. SY and PZ provided transgenic mice (OT‐1, OT‐2, and ERN1^fl/fl^), and ZX and WW provided Carfilzomib solution.

## Conflict of interest

The authors declare that they have no conflict of interest.

## Supporting information



Appendix

Expanded View Figures PDF

Source Data for Expanded View and Appendix

Source Data for Figure 5

Source Data for Figure 6

## Data Availability

This study includes no data deposited in external repositories.
