# Supplementary material for: Carfilzomib modulates tumor microenvironment to potentiate immune checkpoint therapy for cancer
Source: EMBO Mol Med. 2021 Dec 13;14(1):e14502. doi: 10.15252/emmm.202114502 (PMC8749493; doi:10.15252/emmm.202114502)
Supplement: Supplementary file 1 — Appendix [file EMMM-14-e14502-s005.pdf]

Appendix Figure S1	Page 1
Appendix Figure S1-Figure legends	Page 2-3
Appendix Figure S2	Page 4
Appendix Figure S2-Figure legends	Page 5
Appendix Figure S3	Page 6
Appendix Figure S3-Figure legends	Page 7
Appendix Table S1: Antibody dilution list	Page 8
Appendix Table S2: p-Value	Page 9-16

Appendix Figure S1

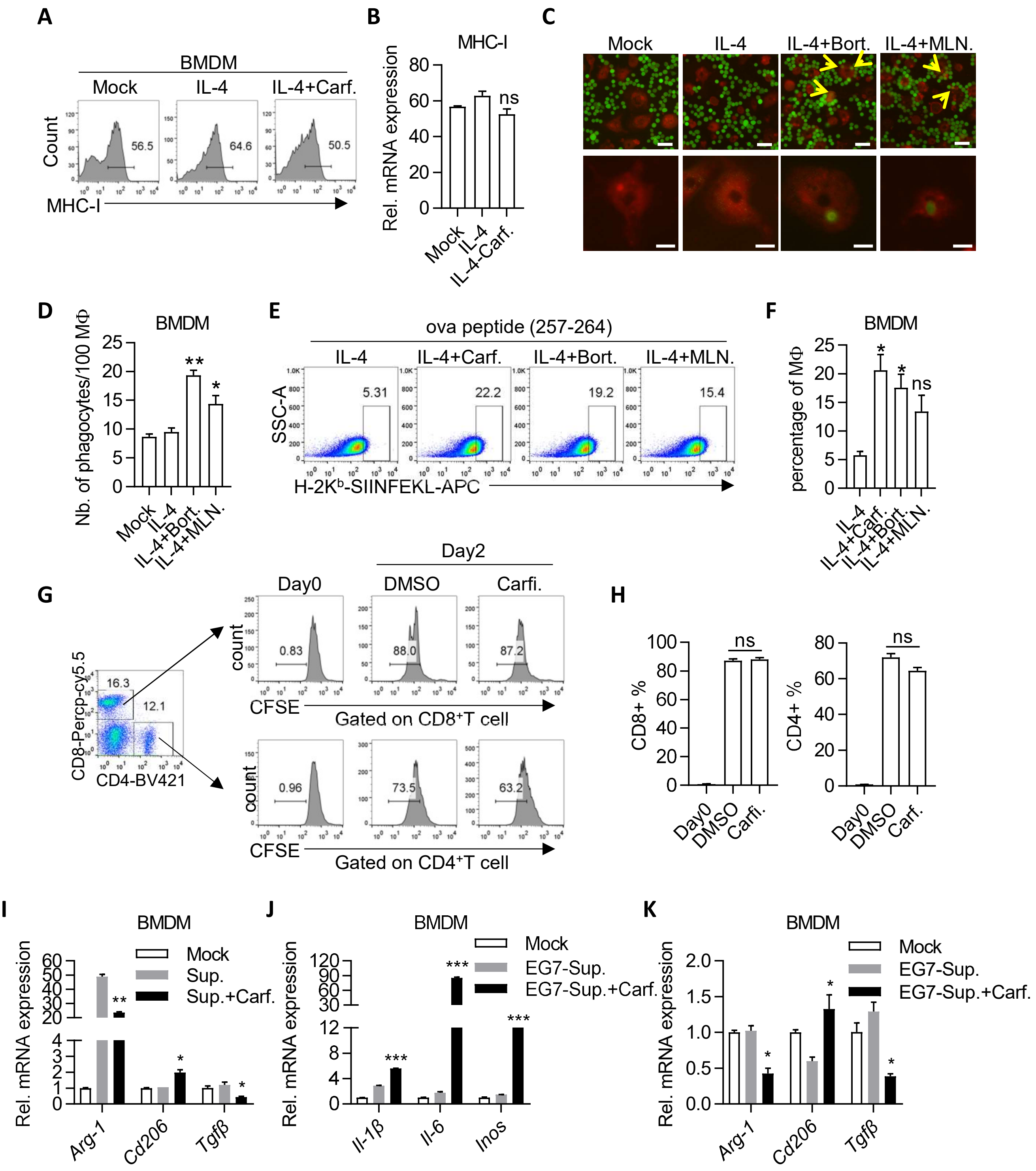

Appendix Figure S1. Carfilzomib, Bortezomib and MLN9708 reprogram M2 macrophages into M1-like population

A, B. Carfilzomib can not promote the expression of MHC-I in BMDMs. BMDMs were treated as described in Figure 2C, and the representative histogram of MHC-I expression was shown by flow cytometry 12 hours after stimulation (A). Statistics represent the proportion of MHC-I positive cells in BMDMs (B). The MHC-I^+ population according to isotype-matched antibody staining control.

C, D. Bortezomib and MLN9708 promote the phagocytic ability of macrophages. BMDMs were pretreated by IL-4 (20 ng/mL) for 24 hours, then stimulated by DMSO, Bortezomib (500 nM) or MLN9708 (500 nM) for 12 hours. After starving for 2 hours and stained with Deep Red membrane dye, BMDMs (red) were incubated with L1210-GFP cells (green) as targets in serum-free medium for another 2 hours. Phagocytosis effect was observed and photographed under fluorescence microscope (C). Statistics for the amount of phagocytosis of L1210 in 100 macrophages (D). Scale bars, 50 μm (up), 20 μm (down). The yellow arrows indicated L1210 that is phagocytosed by macrophages.

E, F. Carfilzomib, Bortezomib and MLN9708 promote the capacity of antigen presentation in macrophages. BMDMs were pretreated by IL-4 (20 ng/mL) for 24 hours, then stimulated by DMSO, Carfilzomib (500 nM), Bortezomib (500 nM) or MLN9708 (500 nM) for 12 hours. $OVA_{257\text{-}264}$ peptides were then transfected into BMDMs and the complex of H-2K^b-SIINFEKL was detected by flow cytometry with antibody 25-D1.16.(E). Statistics represent the percentage of H-2K^b-SIINFEKL positive cells in BMDMs (F).

G, H. Carfizomib have no direct effect on the proliferation of CD4 and CD8 T cells. Spleencytes from B6 mice were induced by anti-CD3 and anti-CD28, and treated by DMSO or Carfilzomib (500 nM) for 2 days at the same time. CFSE was detected through flow cytometry after 2 days (G). Statistics represent and the intensity of CFSE and reflect the proliferation of T cells (H).

I. Carfilzomib suppresses the M2-polarization of macrophages acclimated by TSN of L1210. After taming to TAMs with TSN of L1210 cells, BMDMs were stimulated by DMSO or Carfilzomib (1 μM). RNA was extracted from cells and the expression of *Arg-1, Tgf-β, Cd206* were quantified through RT-qPCR 6 hours after stimulation.

J, K. Carfilzomib promotes the M1-polarization and suppresse the M2-polarization of macrophages acclimated by TSN of EG7. After taming to TAMs with tumor culture supernatant (TSN) produced by EG7 cells, BMDMs were stimulated by DMSO or Carfilzomib (1 μM). RNA was extracted from cells and the expression of *Il-1β*, *Il-6, Inos, Arg-1, Tgf-β, Cd206* were quantified through RT-qPCR 6 hours after stimulation.

Data information: Data from three experiments are presented as the mean ± SD. T test was used for statistical analysis of differences between groups. *p < 0.05, **p < 0.01, ***p < 0.001 (student's t-test).

Appendix Figure S2

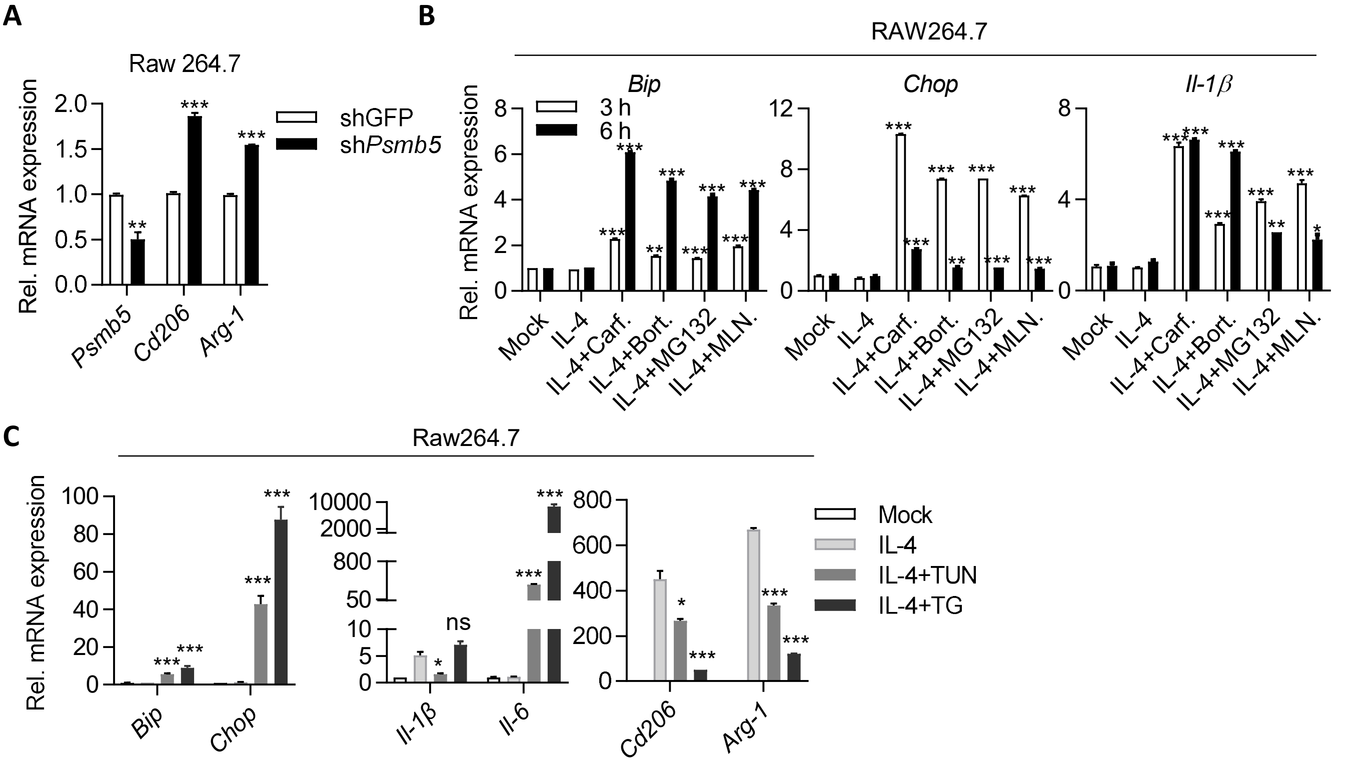

Appendix Figure S2. Proteasomal inhibitors reprogram M2 macrophages toward M1-like macrophages by inducing ER stress signaling

A. Expression of M2 macrophage markers in IL-4 activated Raw264.7 with β5 subunit of proteasome knockdown. Raw264.7(shGFP or sh*Psmb5*) was activated with IL-4 (20 ng/mL) for 24 hours. RNA was extracted and expression of *Cd206, Arg-1* were quantified through RT-qPCR.

B. Carfilzomib, Bortezomib, MLN9708 and MG132 induce ER stress response and *Il-1β* in Raw264.7. Raw264.7 cells were pretreated with IL-4 (20 ng/mL) for 24 hours and then stimulated by DMSO, Carfilzomib (1 μM), Bortezomib (1 μM), MLN9708 (2 μM) or MG132 (5 μM) for 3 or 6 hours. RNA was extracted for quantifying expression of ER stress related genes (*Bip*, *Chop*) and *Il-1β* through RT-qPCR.

C. TUN and TG promote the expression of M1 macrophage markers and ER stress related genes and reduce the expression of M2 macrophage markers in Raw264.7. Raw264.7 cells were pretreated with IL-4 (20 ng/mL) for 24 hours and then stimulated by DMSO, TUN (500 nM) or TG (500 nM) for 6 hours. RNA was extracted for quantifying expression of *Bip, Chop, Il-1β*, *Il-6*, *Cd206* and *Arg1* through RT-qPCR.

Data information: Data from three experiments are presented as the mean ± SD. T test was used for statistical analysis of differences between groups. *$p < 0.05$, **$p < 0.01$, ***$p < 0.001$ (student’s t-test).

Appendix Figure S3

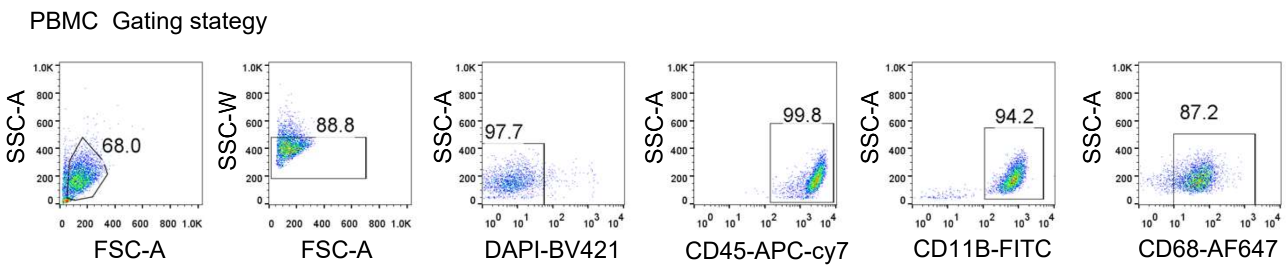

Appendix Figure S3. Proteasome inhibitors are capable of reprogramming human M2 macrophages into M1-like macrophages

Gating strategy for analyzing the expression of CD80 or CD206 in macrophages derived from PBMCs. After gating the single (by FSC-A and SSC-W) and living cells (DAPI negative), CD80 or CD206 positive cells were analyzed by gating on $CD45^{+}CD11B^{+}CD68^{+}$ population.

Appendix Table S1: Antibody dilution list

Antibody	Company	Catalog Number	Dilution
CD45-FITC anti-mouse	BioLegend	103108	1/100
CD45-APC anti-mouse	BD Biosciences	559664	1/100
CD45.1-FITC anti-mouse	Biolegend	110705	1/100
CD45.2-APC anti-mouse	Biolegend	109813	1/100
CD11B-APC anti-mouse	eBioscience	17-0112-81	1/100
F4/80-BV650 anti-mouse	BD Biosciences	743280	1/100
CD80-PE anti-mouse	Biolegend	104707	1/100
CD86-PE anti-mouse	Biolegend	105007	1/100
CD206-PE anti-mouse	eBioscience	12-2061-80	1/100
MHCI-FITC anti-mouse	eBioscience	11-5958-80	1/100
MHCII-BV421- anti-mouse	BD Biosciences	562928	1/100
CD3e- APC-cy™7 anti-mouse	BD Biosciences	557596	1/100
CD8a-PerCP-Cyanin5.5 anti-mouse	eBioscience	45-0081-82	1/100
CD4- PerCP-Cyanin5.5 anti-mouse	BioLegend	100433	1/100
CD69-PE anti-mouse	BioLegend	104507	1/50
CD25-APC anti-mouse	eBioscience	17-0251-82	1/50
H-2K^{b}-SIINFEKL-APC anti-mouse	BioLegend	141606	1/100
CD45- APC-cy™7 anti-human	BD Biosciences	557833	1/100
CD11B-FITC anti-human	BD Biosciences	557396	1/100
CD68-AF647-anti-human	BD Biosciences	562111	1/100
CD80-BV605-anti-human	BD Biosciences	563315	1/100
CD206-PE anti-human	BD Biosciences	555954	1/100
Anti-mouse CD28	eBiocience	16-0281-82	1 μg/mL
Anti-mouse CD3e	eBiocience	16-0031-85	2 μg/mL
Anti-mouse PD-1	BioXcell	BP0033-2	10 mg/kg
Anti-mouse CD8a	BioXcell	BE0061	10 mg/kg
Anti-mouse CD4	BioXcell	BP0003-3	10 mg/kg
Ki-67	Abcam	ab15580	1/500
β-Actin	Sigma-Aldrich	A5316	1/5000
IRE1 alpha	CST	3294S	1/1000
p-IRE1 alpha	Abcam	ab226974	1/1000
IκB alpha	Abcam	ab32518	1/1000
p-IκB alpha	Abcam	ab133462	1/1000
P65	Abcam	ab16502	1/1000
p-P65	Abcam	ab86299	1/1000
TRAF2	CST	4724S	1/1000
Anti-mouse IgG	Sigma-Aldrich	A0168	1/2000
Anti-Rabbit IgG	Sigma-Aldrich	A6154	1/2000
Anti-Rabbit IgG (Light-Chain specific)	CST	93702S	1/1000
Anti-Rabbit IgG Isotype Control	CST	3900S	1/1000

Appendix Table S2: p-Value

Figure 1F	p-Value	Test
IL-4+Carf./IL-4	0.0007	student’s t-test
IL-4+Bort./IL-4	0.0003	student’s t-test
IL-4+MLN./IL-4	<0.0001	student’s t-test
Figure 1G	p-Value	Test
IL-4+Carf./IL-4	<0.0001	student’s t-test
IL-4+Bort./IL-4	<0.0001	student’s t-test
IL-4+MLN./IL-4	<0.0001	student’s t-test
Figure 1H	p-Value	Test
IL-4+Carf./IL-4	0.0015	student’s t-test
IL-4+Bort./IL-4	0.0032	student’s t-test
IL-4+MLN./IL-4	0.0094	student’s t-test
Figure 1I	p-Value	Test
IL-4+Carf./IL-4	0.0001	student’s t-test
IL-4+Bort./IL-4	0.0002	student’s t-test
IL-4+MLN./IL-4	0.0004	student’s t-test

Figure 2A	p-Value	Test
Il-6 (IL-4+Carf./IL-4)	0.0002	student’s t-test
Inos (IL-4+Carf./IL-4)	<0.0001	student’s t-test
Cd206 (IL-4+Carf./IL-4)	0.0003	student’s t-test
Arg1 (IL-4+Carf./IL-4)	0.0007	student’s t-test
Figure 2B	p-Value	Test
IL-6 (IL-4+Carf./IL-4)	<0.0001	
TNFa (IL-4+Carf./IL-4)	0.0037	
Figure 2D	p-Value	Test
CD86 (IL-4+Carf./IL-4)	0.0167	student’s t-test
CD80 (IL-4+Carf./IL-4)	0.0043	student’s t-test
MHC-II (IL-4+Carf./IL-4)	0.0082	student’s t-test
Cd206 (IL-4+Carf./IL-4)	0.0089	student’s t-test
Figure 2F	p-Value	Test
IL-4+Carf./IL-4	0.01	student’s t-test
Figure 2H	p-Value	Test
IL-4+Carf./IL-4	0.0075	student’s t-test
Figure 2J	p-Value	Test
IL-4+Carf./IL-4	0.0167	student’s t-test
Figure 2L	p-Value	Test
IL-4+Carf./IL-4	0.0495	student’s t-test
Figure 2M	p-Value	Test
Il-1β（Sup.+Carf./Sup.）	0.0029	student’s t-test
Il-6（Sup.+Carf./Sup.）	P<0.0001	student’s t-test
Inos（Sup.+Carf./Sup.）	P<0.0001	student’s t-test

Figure 3A	p-Value	Test
IL-4+Carf./IL-4	P<0.0001	student’s t-test
IL-4+MG132/IL-4	0.0024	student’s t-test
Figure 3B	p-Value	Test
Carf.+IL-4/IL-4	0.0149	student’s t-test
MG132+IL-4/IL-4	0.0093	student’s t-test
Figure 3C	p-Value	Test
Il-1β（IL-4+MG132/IL-4)	P<0.0001	student’s t-test
Il-6（IL-4+MG132/IL-4)	0.0007	student’s t-test
Inos（IL-4+MG132/IL-4)	P<0.0001	student’s t-test
Cd206（IL-4+MG132/IL-4)	0.0123	student’s t-test
Arg1（IL-4+MG132/IL-4)	0.0346	student’s t-test

Figure 3D	p-Value	Test
Psmb5（shPsmb5/shGFP)	0.0005	student’s t-test
Il-1β（shPsmb5/shGFP)	0.001	student’s t-test
Il-6（shPsmb5/shGFP)	0.0296	student’s t-test
Figure 3E（Bip）	p-Value	Test
3hours (IL-4+Carf./IL-4)	0.0371	student’s t-test
3hours (IL-4+Bort./IL-4)	0.0032	student’s t-test
3hours (IL-4+MG132/IL-4)	0.0048	student’s t-test
3hours (IL-4+MLN./IL-4)	0.0291	student’s t-test
6hours (IL-4+Carf./IL-4)	0.0042	student’s t-test
6hours (IL-4+Bort./IL-4)	0.0042	student’s t-test
6hours (IL-4+MG132/IL-4)	0.0022	student’s t-test
6hours (IL-4+MLN./IL-4)	0.0045	student’s t-test
Figure 3E（Chop）	p-Value	Test
3hours (IL-4+Carf./IL-4)	0.0006	student’s t-test
3hours (IL-4+Bort./IL-4)	0.0002	student’s t-test
3hours (IL-4+MG132/IL-4)	0.0004	student’s t-test
3hours (IL-4+MG132/IL-4)	0.0097	student’s t-test
6hours (IL-4+Carf./IL-4)	0.0009	student’s t-test
6hours (IL-4+Bort./IL-4)	0.0053	student’s t-test
6hours (IL-4+MG132/IL-4)	0.0049	student’s t-test
6hours (IL-4+MG132/IL-4)	0.0031	student’s t-test
Figure 3E（Il-1b）	p-Value	Test
3hours (IL-4+Carf./IL-4)	0.0035	student’s t-test
3hours (IL-4+Bort./IL-4)	0.0054	student’s t-test
3hours (IL-4+MG132/IL-4)	0.0012	student’s t-test
3hours (IL-4+MLN./IL-4)	0.0063	student’s t-test
6hours (IL-4+Carf./IL-4)	0.0031	student’s t-test
6hours (IL-4+Bort./IL-4)	0.0028	student’s t-test
6hours (IL-4+MG132/IL-4)	0.0063	student’s t-test
6hours (IL-4+MLN./IL-4)	0.0041	student’s t-test
Figure 3F	p-Value	Test
Il-1β（IL-4+TUN/IL-4)	0.0611	student’s t-test
Il-1β（IL-4+TG/IL-4)	0.0017	student’s t-test
Il-6（IL-4+TUN/IL-4)	0.0001	student’s t-test
Il-6（IL-4+TG/IL-4)	0.001	student’s t-test
Cd206（IL-4+TUN/IL-4)	0.0016	student’s t-test
Cd206（IL-4+TG/IL-4)	0.0009	student’s t-test
Arg1（IL-4+TUN/IL-4)	0.0335	student’s t-test
Arg1（IL-4+TG/IL-4)	0.0007	student’s t-test
Bip（IL-4+TUN/IL-4)	0.0001	student’s t-test
Bip（IL-4+TG/IL-4)	0.0056	student’s t-test
Chop（IL-4+TUN/IL-4)	0.0004	student’s t-test
Chop（IL-4+TG/IL-4)	0.0031	student’s t-test
Figure 3G	p-Value	Test
Il-1β（IL-4+4-PBA+Carf./IL-4+Carf.)	0.0028	student’s t-test
Il-1β（IL-4+4-PBA+Bort./IL-4+Bort.)	p<0.0001	student’s t-test
Il-1β（IL-4+4-PBA+MLN./IL-4+MLN..)	0.0001	student’s t-test
Il-1β（IL-4+4-PBA+MG132/IL-4+MG132)	p<0.0001	student’s t-test
Il-6（IL-4+4-PBA+Carf./IL-4+Carf.)	0.0002	student’s t-test
Il-6（IL-4+4-PBA+Bort./IL-4+Bort.)	0.0002	student’s t-test
Il-6（IL-4+4-PBA+MLN./IL-4+MLN..)	0.0115	student’s t-test
Il-6（IL-4+4-PBA+MG132/IL-4+MG132)	0.0023	student’s t-test
Inos（IL-4+4-PBA+Carf./IL-4+Carf.)	0.0013	student’s t-test
Inos（IL-4+4-PBA+Bort./IL-4+Bort.)	0.0007	student’s t-test
Inos（IL-4+4-PBA+MLN./IL-4+MLN.)	0.0046	student’s t-test
Inos（IL-4+4-PBA+MG132/IL-4+MG132)	0.0001	student’s t-test

Figure 4A (Il-1β)	p-Value	Test
IL-4+Carf. ($Ern1^{-/-}$/WT)	0.0002	student's t-test
Figure 4A (Il-6)	p-Value	Test
IL-4+Carf. ($Ern1^{-/-}$/WT)	0.005	student's t-test
Figure 4B (Il-1β)	p-Value	Test
IL-4+Carf. ($Ern1^{-/-}$/WT)	0.001	student's t-test
Figure 4B (Il-6)	p-Value	Test
IL-4+Carf. ($Ern1^{-/-}$/WT)	0.0001	student's t-test
Figure 4C	p-Value	Test
Carf. (Kira6/control)	0.0039	student's t-test
Carf. (4μ8c/control)	0.2158	student's t-test
Figure 4E	p-Value	Test
Bip (IL-4+Kira6+Carf./IL-4+Carf.)	0.0178	student's t-test
Chop (IL-4+Kira6+Carf./IL-4+Carf.)	0.0351	student's t-test
Il-1β (IL-4+Kira6+Carf./IL-4+Carf.)	0.005	student's t-test
Il-6 (IL-4+Kira6+Carf./IL-4+Carf.)	0.0088	student's t-test
Figure 4F	p-Value	Test
sXBP1 (IL-4+Carf./IL-4)	0.1185	student's t-test
sXBP1 (IL-4+TUN/IL-4)	0.0019	student's t-test
usXBP1 (IL-4+Carf./IL-4)	0.1862	student's t-test
usXBP1 (IL-4+TUN/IL-4)	0.0059	student's t-test
Figure 4G	p-Value	Test
sXBP1 (IL-4+Carf./IL-4)	0.1564	student's t-test
sXBP1 (IL-4+TUN/IL-4)	0.0003	student's t-test
usXBP1 (IL-4+Carf./IL-4)	0.0615	student's t-test
usXBP1 (IL-4+TUN/IL-4)	<0.0001	student's t-test
Figure 4K	p-Value	Test
IL-4+Carf./IL-4	0.0024	student's t-test

Figure 5B	p-Value	Test
Vehicle (PreRx/PstRx)	0.0231	student's t-test
Carf. (PreRx/PstRx)	0.0093	student's t-test
Clod. (PreRx/PstRx)	0.2537	student's t-test
Carf.+Clod. (PreRx/PstRx)	0.031	student's t-test
Figure 5D	p-Value	Test
Area (Vehicle/Carf.)	0.0005	student's t-test
Area (Vehicle/Clod.)	0.0692	student's t-test
Area (Vehicle/Carf.+Clod.)	0.011	student's t-test
Nb. (Vehicle/Carf.)	0.0018	student's t-test
Nb. (Vehicle/Clod.)	0.2939	student's t-test
Nb. (Vehicle/Carf.+Clod.)	0.0308	student's t-test
Figure 5F	p-Value	Test
CD206 (Vehicle/Carf.)	0.0028	student's t-test
CD80 (Vehicle/Carf.)	0.0084	student's t-test
Figure 5H	p-Value	Test
CD8 (Vehicle/Carf.)	0.0039	student's t-test
CD69 (Vehicle/Carf.)	0.0012	student's t-test
Figure 5J	p-Value	Test
Vehicle (PreRx/PstRx)	0.0202	student's t-test
DT (PreRx/PstRx)	0.0222	student's t-test
Carf. (PreRx/PstRx)	0.0013	student's t-test
Carf.+DT (PreRx/PstRx)	0.1129	student's t-test
Figure 5L	p-Value	Test
WT-Carf./WT-Vehicle	<0.0001	student's t-test
LyzM-cre-Carf./LyzM-cre-Vehicle	0.0129	student's t-test

Figure 6A	p-Value	Test
WT-Veh./WT-Carf.	<0.0001	student’s t-test
RAG1$^{-/-}$-Veh./RAG1$^{-/-}$-Carf.	0.0141	student’s t-test
Figure 6C	p-Value	Test
WT-Veh./WT-Carf.	<0.0001	student’s t-test
RAG1$^{-/-}$-Veh./RAG1$^{-/-}$-Carf.	0.0104	student’s t-test
Figure 6I	p-Value	Test
CD69 (Vehile./PD1-Ab)	0.029	student’s t-test
CD69 (Vehile./Carf.)	0.0296	student’s t-test
CD69 (Vehile./PD1-Ab +Carf.)	0.0015	student’s t-test
CD25 (Vehile./PD1-Ab)	0.0537	student’s t-test
CD25 (Vehile./Carf.)	0.0048	student’s t-test
CD25 (Vehile./PD1-Ab +Carf.)	0.0013	student’s t-test

Figure 7A	p-Value	Test
Il-1β（IL-4+Carf./IL-4)	0.0007	student’s t-test
Il-1β（IL-4+Bort./IL-4)	0.0143	student’s t-test
Il-1β（IL-4+MLN./IL-4)	0.6815	student’s t-test
IL-6（IL-4+Carf./IL-4)	0.0001	student’s t-test
IL-6（IL-4+Bort./IL-4)	0.0042	student’s t-test
IL-6（IL-4+MLN./IL-4)	0.0045	student’s t-test
TNF-a（IL-4+Carf./IL-4)	0.0033	student’s t-test
TNF-a（IL-4+Bort./IL-4)	0.0087	student’s t-test
TNF-a（IL-4+MLN./IL-4)	0.0388	student’s t-test
IL-10（IL-4+Carf./IL-4)	0.0003	student’s t-test
IL-10（IL-4+Bort./IL-4)	0.0027	student’s t-test
IL-10（IL-4+MLN./IL-4)	0.0002	student’s t-test
TGF-b（IL-4+Carf./IL-4)	0.0004	student’s t-test
TGF-b（IL-4+Bort./IL-4)	0.0028	student’s t-test
TGF-b（IL-4+MLN./IL-4)	0.6684	student’s t-test
Figure 7B	p-Value	Test
Il-1β（IL-4+Carf./IL-4)	0.0015	student’s t-test
Il-1β（IL-4+Bort./IL-4)	0.0006	student’s t-test
Il-1β（IL-4+MLN./IL-4)	0.0039	student’s t-test
IL-6（IL-4+Carf./IL-4)	0.0072	student’s t-test
IL-6（IL-4+Bort./IL-4)	0.0019	student’s t-test
IL-6（IL-4+MLN./IL-4)	0.0015	student’s t-test
TNF-a（IL-4+Carf./IL-4)	0.0249	student’s t-test
TNF-a（IL-4+Bort./IL-4)	0.035	student’s t-test
TNF-a（IL-4+MLN./IL-4)	0.0171	student’s t-test
Figure 7D	p-Value	Test
IL-4+Carf./IL-4	0.0096	student’s t-test
IL-4+Bort./IL-4	0.0327	student’s t-test
IL-4+MLN./IL-4	0.0254	student’s t-test
Figure 7F	p-Value	Test
IL-4+Carf./IL-4	0.0062	student’s t-test
IL-4+Bort./IL-4	0.0148	student’s t-test
IL-4+MLN./IL-4	0.0125	student’s t-test
Figure 7H	p-Value	Test
IL-4+Carf./IL-4	0.0229	student’s t-test
IL-4+Bort./IL-4	0.0113	student’s t-test
IL-4+MLN./IL-4	0.0491	student’s t-test

Figure EV1A	p-Value	Test
M0+LPS 6h/M0	0.0003	student’s t-test
M0+LPS 12h/M0	0.0065	student’s t-test
M0+IL-4 6h/M0	0.3429	student’s t-test
M0+IL-4 12h/M0	0.0634	student’s t-test
Figure EV1B	p-Value	Test
Il-1β (LPS 6h/M0)	0.0002	student’s t-test
Il-1β (LPS 12h/M0)	0.0008	student’s t-test
Il-6 (LPS 6h/M0)	0.0009	student’s t-test
Il-6 (LPS 12h/M0)	<0.0001	student’s t-test
Il-6 (IL-4 6h/M0)	0.077	student’s t-test
Il-6 (IL-4 12h/M0)	0.0575	student’s t-test
Cd206 (IL-4 6h/M0)	0.0001	student’s t-test
Cd206 (IL-4 12h/M0)	<0.0001	student’s t-test
Figure EV1C	p-Value	Test
3h (IL-4+MLN./IL-4)	0.0001	student’s t-test
3h (IL-4+Bort./IL-4)	0.0003	student’s t-test
3h (IL-4+Carf./IL-4)	0.0003	student’s t-test
6h (IL-4+MLN./IL-4)	0.0003	student’s t-test
6h (IL-4+Bort./IL-4)	0.0007	student’s t-test
6h (IL-4+Carf./IL-4)	0.0007	student’s t-test

Figure EV2A	p-Value	Test
Il-6 (IL-4+Bort./IL-4)	0.0001	student’s t-test
Il-6 (IL-4+MLN./IL-4)	0.0004	student’s t-test
Inos (IL-4+Bort./IL-4)	0.0017	student’s t-test
Inos (IL-4+MLN./IL-4)	0.0033	student’s t-test
Cd206 (IL-4+Bort./IL-4)	0.0066	student’s t-test
Cd206 (IL-4+MLN./IL-4)	0.0002	student’s t-test
Arg1 (IL-4+Bort./IL-4)	0.0036	student’s t-test
Arg1 (IL-4+MLN./IL-4)	0.0324	student’s t-test
Figure EV2B	p-Value	Test
Il-6 (IL-4+Carf./IL-4)	0.0004	student’s t-test
Il-6 (IL-4+Bort./IL-4)	p<0.0001	student’s t-test
Il-6 (IL-4+MLN./IL-4)	0.0001	student’s t-test
Inos (IL-4+Carf./IL-4)	0.0033	student’s t-test
Inos (IL-4+Bort./IL-4)	0.0064	student’s t-test
Inos (IL-4+MLN./IL-4)	0.0014	student’s t-test
Cd206 (IL-4+Carf./IL-4)	0.0001	student’s t-test
Cd206 (IL-4+Bort./IL-4)	0.0019	student’s t-test
Cd206 (IL-4+MLN./IL-4)	0.0004	student’s t-test
Arg1 (IL-4+Carf./IL-4)	0.0001	student’s t-test
Arg1 (IL-4+Bort./IL-4)	0.0029	student’s t-test
Arg1 (IL-4+MLN./IL-4)	0.0044	student’s t-test
Figure EV2C	p-Value	Test
IL-6 (IL-4+Bort./IL-4)	0.0002	student’s t-test
IL-6 (IL-4+MLN./IL-4)	0.0006	student’s t-test
TNFa (IL-4+Bort./IL-4)	0.0211	student’s t-test
TNFa (IL-4+MLN./IL-4)	0.0482	student’s t-test
Figure EV2D	p-Value	Test
IL-6 (IL-4+Carf./IL-4)	p<0.0001	student’s t-test
IL-6 (IL-4+Bort./IL-4)	0.0003	student’s t-test
IL-6 (IL-4+MLN./IL-4)	0.0005	student’s t-test

Figure EV2E	p-Value	Test
BMDM-Il-1β（Carf./Mock)	0.0004	student’s t-test
BMDM-Il-6 （Carf./Mock)	<0.0001	student’s t-test
BMDM-Inos（Carf./Mock)	0.0009	student’s t-test
Raw264.7-Il-1β (Carf./Mock)	0.0009	student’s t-test
Raw264.7-Il-6 (Carf./Mock)	<0.0001	student’s t-test
Raw264.7-Inos (Carf./Mock)	0.0005	student’s t-test
Figure EV2H	p-Value	Test
CD86 (IL-4+Bort./IL-4)	0.0448	student’s t-test
CD86 (IL-4+MLN./IL-4)	0.0206	student’s t-test
CD206 (IL-4+Bort./IL-4)	0.0027	student’s t-test
CD206 (IL-4+MLN./IL-4)	0.0023	student’s t-test
Figure EV2J	p-Value	Test
CD86 (IL-4+Carf./IL-4)	0.0003	student’s t-test
CD86 (IL-4+Bort./IL-4)	0.0005	student’s t-test
CD86 (IL-4+MLN./IL-4)	0.0011	student’s t-test
CD206 (IL-4+Carf./IL-4)	0.0013	student’s t-test
CD206 (IL-4+Bort./IL-4)	0.002	student’s t-test
CD206 (IL-4+MLN./IL-4)	0.0213	student’s t-test
Figure EV2L	p-Value	Test
IL-4+Bort./IL-4	0.0065	student’s t-test
IL-4+MLN./IL-4	0.0113	student’s t-test
EV2N	p-Value	Test
IL-4+Carf./IL-4	0.0641	student’s t-test
Figure EV2P	p-Value	Test
IL-4+Carf./IL-4	0.0176	student’s t-test
IL-4+Bort./IL-4	0.0205	student’s t-test
IL-4+MLN./IL-4	0.0655	student’s t-test
Figure EV2R	p-Value	Test
CD8（Carf./DMSO)	0.5231	student’s t-test
CD4（Carf./DMSO)	0.0606	student’s t-test
Figure EV2S	p-Value	Test
Arg-1 (Sup.+Carf./Sup)	0.0023	student’s t-test
Cd206 (Sup.+Carf./Sup)	0.0181	student’s t-test
Tgfb (Sup.+Carf./Sup)	0.0276	student’s t-test
Figure EV2T	p-Value	Test
Il-1β (Sup.+Carf./Sup)	p<0.0001	student’s t-test
Il-6 (Sup.+Carf./Sup)	0.0002	student’s t-test
Inos (Sup.+Carf./Sup)	0.0008	student’s t-test
Figure EV2U	p-Value	Test
Arg-1 (Sup.+Carf./Sup)	0.0147	student’s t-test
Cd206 (Sup.+Carf./Sup)	0.038	student’s t-test
Tgfb (Sup.+Carf./Sup)	0.0111	student’s t-test

Figure EV3A	p-Value	Test
Atf6（shAtf6/shGFP)	0.0032	student’s t-test
Perk（shPerk/shGFP)	0.0022	student’s t-test
Figure EV3D（Il-1β)	p-Value	Test
IL-4+Bort.（$Ern1^{-/-}$/WT)	0.021	student’s t-test
IL-4+MLN.（$Ern1^{-/-}$/WT)	0.0038	student’s t-test
Figure EV3D（Il-6)	p-Value	Test
IL-4+Bort.（$Ern1^{-/-}$/WT)	0.0008	student’s t-test
IL-4+MLN.（$Ern1^{-/-/}$WT)	0.0075	student’s t-test
Figure EV3E（Il-1β)	p-Value	Test
IL-4+Bort.（$Ern1^{-/-}$/WT)	0.0018	student’s t-test
IL-4+MLN.（$Ern1^{-/-}$/WT)	0.002	student’s t-test
Figure EV3E（Il-6)	p-Value	Test

IL-4+Bort.（$Ern1^{-/-}$/WT）	0.0002	student’s t-test
IL-4+MLN.（$Ern1^{-/-}$/WT）	0.0105	student’s t-test
EV3F（Il-1β）	p-Value	Test
IL-4+Carf.（shAtf6/shGFP）	0.6053	student’s t-test
Figure EV3F（Il-6）	p-Value	Test
IL-4+Carf.（shAtf6/shGFP）	0.5354	student’s t-test
Figure EV3G（Il-1β）	p-Value	Test
IL-4+Carf.（shPerk/shGFP）	0.9298	student’s t-test
Figure EV3G（Il-6）	p-Value	Test
IL-4+Carf.（shPerk/shGFP）	0.9307	student’s t-test
Figure EV3H	p-Value	Test
Il-1β（IL-4+Kira6+Carf./IL-4+Carf.）	0.0042	student’s t-test
Il-1β（IL-4+4μ8c+Carf./IL-4+Carf.）	0.3663	student’s t-test
Il-6（IL-4+Kira6+Carf./IL-4+Carf.）	0.0007	student’s t-test
Il-6（IL-4+4μ8c+Carf./IL-4+Carf.）	0.4165	student’s t-test
Figure EV3I	p-Value	Test
Bip（IL-4+Kira6+Carf./IL-4+Carf.）	0.0034	student’s t-test
Chop（IL-4+Kira6+Carf./IL-4+Carf.）	0.0029	student’s t-test
Il-1β（IL-4+Kira6+Carf./IL-4+Carf.）	0.0028	student’s t-test
Il-6（IL-4+Kira6+Carf./IL-4+Carf.）	0.0232	student’s t-test

Figure EV4B	p-Value	Test
Vehicle/Clod.	0.0001	student’s t-test
Figure EV4C	p-Value	Test
Vehicle/Carf.	0.0871	student’s t-test
Figure EV4D	p-Value	Test
Carf./Vehicle	0.8698	student’s t-test
Figure EV4E	p-Value	Test
DT/Vehicle	0.0046	student’s t-test
Figure EV4F	p-Value	Test
Clod./Vehicle	0.0093	student’s t-test
Figure EV4I	p-Value	Test
Vehicle/Carf.	<0.0001	student’s t-test
Vehicle/Carf.+Clod	0.0105	student’s t-test
Figure EV4K	p-Value	Test
Cd206（Vehicle/Carf.）	0.0013	student’s t-test
Cd80（Vehicle/Carf.）	0.0043	student’s t-test
Cd86（Vehicle/Carf.）	0.0029	student’s t-test
Figure EV4M	p-Value	Test
Vehicle/Clod.	0.0088	student’s t-test

Figure EV5E	p-Value	Test
Control/CD4-Ab	<0.0001	student’s t-test
Figure EV5F	p-Value	Test
Vehicle (PreRx/PstRx)	0.0005	student’s t-test
Carf. (PreRx/PstRx)	0.0012	student’s t-test
CD4-Ab (PreRx/PstRx)	0.0136	student’s t-test
CD8-Ab (PreRx/PstRx)	0.0149	student’s t-test
Figure EV5G	p-Value	Test
Area (Vehile./Carf.)	0.0034	student’s t-test
Area (Vehile./CD4-Ab)	0.0207	student’s t-test
Area (Vehile./CD8-Ab)	0.0249	student’s t-test
Nb. (Vehile./Carf.)	0.0009	student’s t-test
Nb. (Vehile./CD4-Ab)	0.0245	student’s t-test
Nb. (Vehile./CD8-Ab)	0.0124	student’s t-test

Figure EV5H	p-Value	Test
Vehicle (PreRx/PstRx)	0.0314	student’s t-test
PD1-Ab (PreRx/PstRx)	0.1996	student’s t-test
Carf. (PreRx/PstRx)	0.001	student’s t-test
Carf.+PD1-Ab (PreRx/PstRx)	<0.0001	student’s t-test
Figure EV5I	p-Value	Test
Area (Vehile./PD1-Ab)	0.1055	student’s t-test
Area (Vehile./Carf.)	0.002	student’s t-test
Area (Vehile./Carf.+PD1-Ab)	0.0003	student’s t-test
Nb. (Vehile./PD1-Ab)	0.1795	student’s t-test
Nb. (Vehile./Carf.)	0.0023	student’s t-test
Nb. (Vehile./Carf.+PD1-Ab)	0.0006	student’s t-test
Appendix Figure S1A	p-Value	Test
Psmb5（shPsmb5/shGFP）	0.0014	student’s t-test
Cd206（shPsmb5/shGFP）	0.001	student’s t-test
Arg-1（shPsmb5/shGFP）	0.0003	student’s t-test
Appendix Figure S1B（Bip）	p-Value	Test
3hours (IL-4+Carf./IL-4)	<0.0001	student’s t-test
3hours (IL-4+Bort./IL-4)	0.0011	student’s t-test
3hours (IL-4+MG132/IL-4)	0.0008	student’s t-test
3hours (IL-4+MLN./IL-4)	0.0005	student’s t-test
6hours (IL-4+Carf./IL-4)	<0.0001	student’s t-test
6hours (IL-4+Bort./IL-4)	0.0001	student’s t-test
6hours (IL-4+MG132/IL-4)	0.0004	student’s t-test
6hours (IL-4+MLN./IL-4)	<0.0001	student’s t-test
Appendix Figure S1B（Chop）	p-Value	Test
3hours (IL-4+Carf./IL-4)	<0.0001	student’s t-test
3hours (IL-4+Bort./IL-4)	<0.0001	student’s t-test
3hours (IL-4+MG132/IL-4)	<0.0001	student’s t-test
3hours (IL-4+MLN./IL-4)	<0.0001	student’s t-test
6hours (IL-4+Carf./IL-4)	0.0001	student’s t-test
6hours (IL-4+Bort./IL-4)	0.0035	student’s t-test
6hours (IL-4+MG132/IL-4)	0.0007	student’s t-test
6hours (IL-4+MLN./IL-4)	0.001	student’s t-test
Appendix Figure S1B（Il-1β）	p-Value	Test
3hours (IL-4+Carf./IL-4)	0.0004	student’s t-test
3hours (IL-4+Bort./IL-4)	0.0003	student’s t-test
3hours (IL-4+MG132/IL-4)	0.0005	student’s t-test
3hours (IL-4+MLN./IL-4)	0.0004	student’s t-test
6hours (IL-4+Carf./IL-4)	<0.0001	student’s t-test
6hours (IL-4+Bort./IL-4)	0.0001	student’s t-test
6hours (IL-4+MG132/IL-4)	0.0014	student’s t-test
6hours (IL-4+MLN./IL-4)	0.0252	student’s t-test
Appendix Figure S1C	p-Value	Test
Bip（IL-4+TUN/IL-4）	0.0003	student’s t-test
Bip（IL-4+TG/IL-4）	0.0006	student’s t-test
Chop（IL-4+TUN/IL-4）	0.0004	student’s t-test
Chop（IL-4+TG/IL-4）	0.0002	student’s t-test
Il-1β（IL-4+TUN/IL-4）	0.0174	student’s t-test
Il-1β（IL-4+TG/IL-4）	0.0918	student’s t-test
Il-6（IL-4+TUN/IL-4）	0.0005	student’s t-test
Il-6（IL-4+TG/IL-4）	0.0005	student’s t-test
Cd206（IL-4+TUN/IL-4）	0.0204	student’s t-test
Cd206（IL-4+TG/IL-4）	0.0003	student’s t-test
Arg1（IL-4+TUN/IL-4）	0.0005	student’s t-test
Arg1（IL-4+TG/IL-4）	<0.0001	student’s t-test
