# Supplementary material for: Carfilzomib modulates tumor microenvironment to potentiate immune checkpoint therapy for cancer
Source: EMBO Mol Med. 2021 Dec 13;14(1):e14502. doi: 10.15252/emmm.202114502 (PMC8749493; doi:10.15252/emmm.202114502)
Supplement: Supplementary file 2 — Expanded View Figures PDF [file EMMM-14-e14502-s001.pdf]

Expanded View Figures

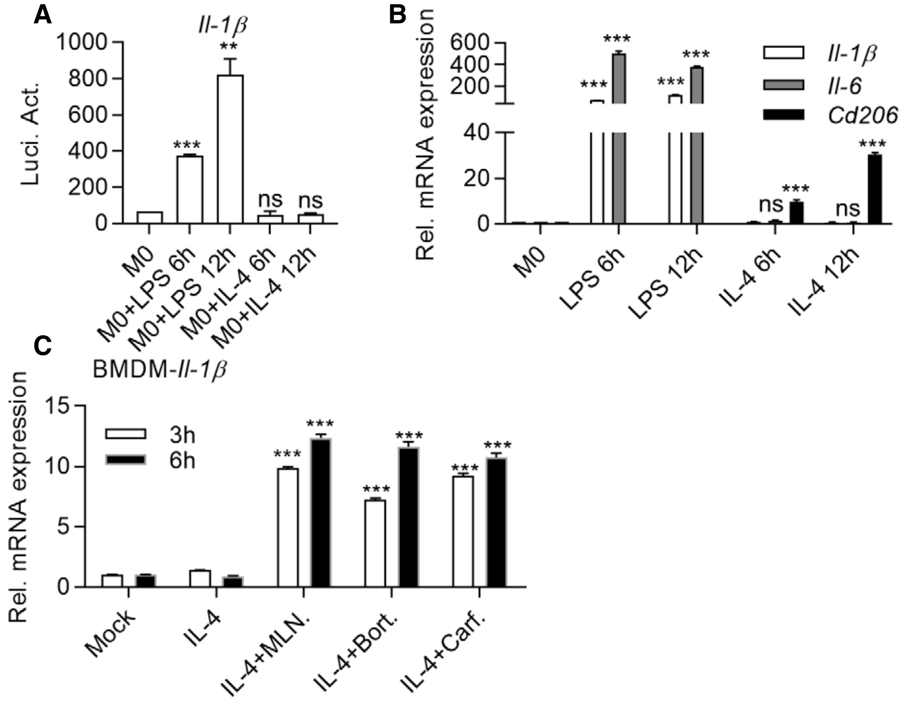

Figure EV1. High-throughput Screening of FDA drugs capable of inducing IL-1β expression in M2 macrophages.

A LPS but not IL-4 significantly activates luciferase activity in M0 *Il-1β*-luciferase transgenic macrophages. BMDMs from *Il-1β*-luciferase transgenic mice were induced by LPS (100 ng/ml) or IL-4 (20 ng/ml). Luciferase assays were performed 6 or 12 h after stimulation. Data are means ± SD of three independent experiments. $^{**}P < 0.01$, $^{***}P < 0.001$ (Student's *t*-test).

B M1 or M2 markers induced in macrophages by LPS or IL-4. BMDMs were induced by LPS (100 ng/ml) or IL-4 (20 ng/ml) for indicated time. RT-qPCR was then performed to quantify the expression of M1/M2 markers. Data are means ± SD of three independent experiments. $^{***}P < 0.001$ (Student's *t*-test).

C Carfilzomib, Bortezomib, and MLN9708 promote the expression of *Il-1β*. BMDMs were pretreated by IL-4 (20 ng/ml) for 24 h, followed by stimulation with DMSO, Carfilzomib (1 μM), Bortezomib (1 μM), or MLN9708 (2 μM) for indicated time. RNA was extracted from cells for quantification of *Il-1β* expression through RT-qPCR. Data are means ± SD of three independent experiments. $^{***}P < 0.001$ (Student's *t*-test).

Figure EV2. Carfilzomib, Bortezomib and MLN9708 reprogram M2 macrophages into M1-like population.

A, B Carfilzomib, Bortezomib, and MLN9708 promote the expression of M1 macrophage markers and reduce the expression of M2 macrophage markers in macrophages. BMDMs (A) and Raw264.7 cells (B) were treated as Fig 1F and G. RNA was extracted from cells and the expression of M1 (*Il-6/Inos*) or M2 (*Cd206/Arg1*) macrophage markers was quantified through RT-qPCR 6 h after stimulation. Data are means ± SD of three independent experiments. $^{*}P < 0.05$, $^{**}P < 0.01$, $^{***}P < 0.001$ (Student's *t*-test).

C, D Carfilzomib, Bortezomib, and MLN9708 promote the secretion of IL-6 and TNFα in M2 macrophages. BMDMs (C) and Raw264.7 cells (D) were pretreated by IL-4 (20 ng/ml) for 24 h, then stimulated by DMSO, Carfilzomib (500 nM), Bortezomib (500 nM), or MLN9708 (500 nM). Secretion of IL-6 and TNFα in culture media was detected through ELISA 24 h after stimulation. Data are means ± SD of three independent experiments. $^{*}P < 0.05$, $^{***}P < 0.001$ (Student's *t*-test).

E Carfilzomib alone promotes the expression of proinflammatory cytokines. BMDMs and Raw264.7 cells were treated by DMSO or Carfilzomib (1 μM) for 6 h, then RNA was extracted from cells, and the expression of *Il-1β/Il-6/Inos* were quantified through RT-qPCR. Data are means ± SD of three independent experiments. $^{***}P < 0.001$ (Student's *t*-test).

F Gating strategy for analyzing M1/M2 macrophage surface markers in macrophages. CD86- or CD206-positive cells were gated on $CD11B^{+}$ cells.

G–J Carfilzomib, Bortezomib, and MLN9708 promote the expression of membrane protein CD86 and reduce CD206. BMDMs (G) and Raw264.7 cells (I) were treated as described in Fig S2C and D. The representative histogram of CD86 and CD206 expression was shown for flow cytometry 12 h after stimulation. (H, J) Statistics represent the proportion of CD86- or CD206-positive cells in BMDMs (H) or Raw264.7 cells (J) under different treatment conditions. Data are means ± SD of three independent experiments. $^{*}P < 0.05$, $^{**}P < 0.01$, $^{***}P < 0.001$ (Student's *t*-test).

© 2021 The Authors

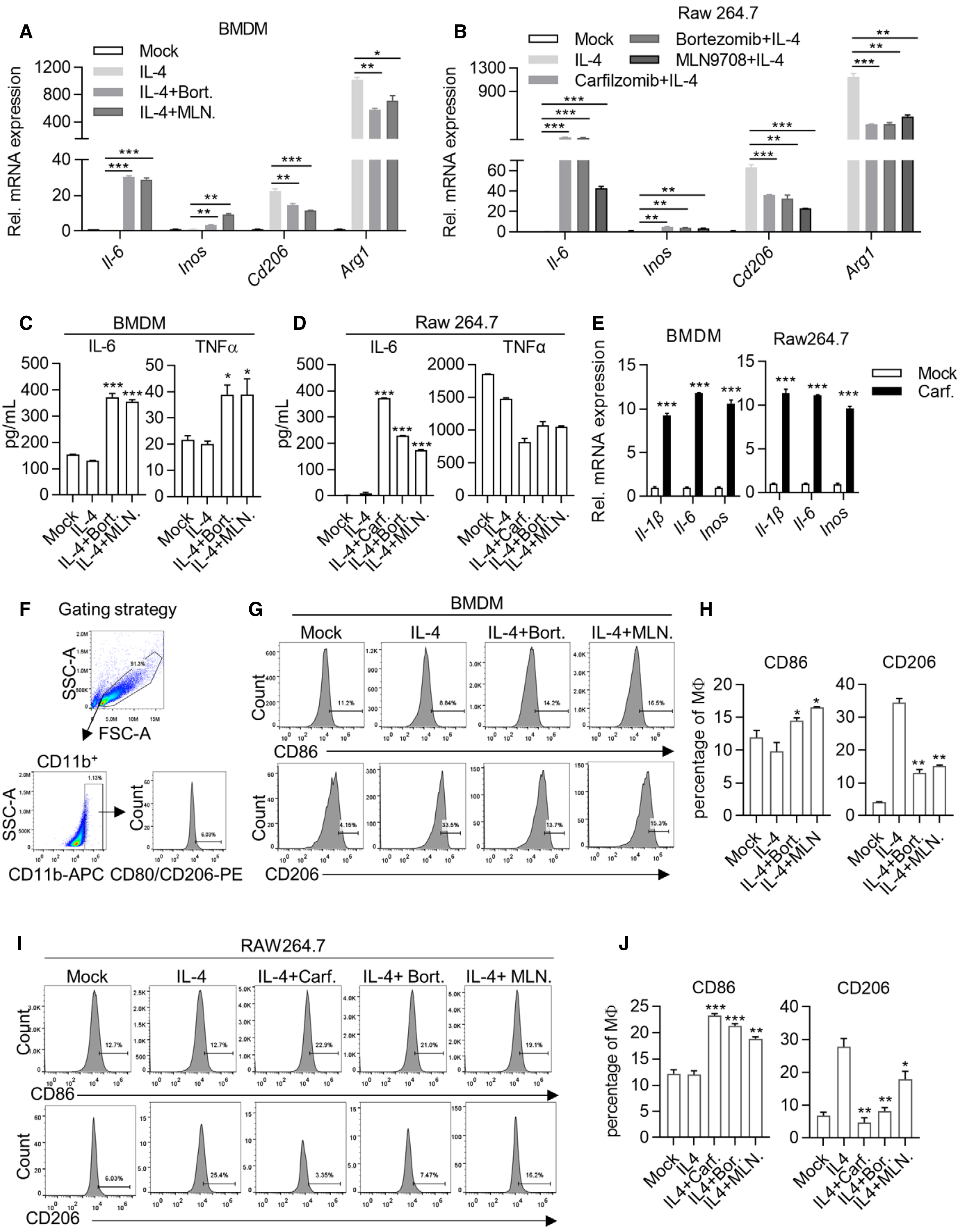

Figure EV2.

© 2021 The Authors

Figure EV3. IRE1α-TRAF2-NF-κB axis is essential for polarizing M2 to M1-like macrophages.

A Knockdown efficiency of sh*Atf6* or sh*Perk*. RNA was extracted from Raw264.7-shGFP, Raw264.7-sh*Atf6*, and Raw264.7-sh*Perk* cells, and the expression of *Atf6* and *Perk* was quantified through RT-qPCR. Data are means ± SD of three independent experiments. $**P < 0.01$ (Student's *t*-test).

B Details of sgRNA-mediated knockout of IRE1α in Raw264.7.

C IRE1α knockout efficiency of sgRNA. Raw264.7-sg IRE1α monoclonal cells were picked out and the WCLs were analyzed by immunoblots with the indicated antibodies.

D, E Deficiency of *Ern1* represses the expression of inflammatory-related genes activated by Bortezomib, MLN9708. Wild-type and $Ern1^{-/-}$ BMDMs (D) or Raw264.7 cells (E) were pretreated by IL-4 (20 ng/ml) for 24 h, and then stimulated with DMSO, Bortezomib (1 μM), or MLN9708 (2 μM) for 6 h. Expression of *Il-1β* and *Il-6* was quantified through RT-qPCR. Data are means ± SD of three independent experiments. $*P < 0.05$, $**P < 0.01$, $***P < 0.001$ (Student's *t*-test).

F, G Knockdown of *Atf6* (F) or *Perk* (G) does not significantly change the ability of Carfilzomib to promote M1-like macrophages polarization. Engineered cell lines, including Raw264.7-shGFP, Raw264.7-sh*Atf6*, or Raw264.7-sh*Perk*, were pretreated by IL-4 (20 ng/ml) for 24 h, then stimulated by DMSO or Carfilzomib (1 μM). RNA was extracted from cells and the expression of *Il-1β* and *Il-6* was quantified through RT-qPCR 6 h after stimulation. Data are means ± SD of three independent experiments.

H IRE1α kinase activity plays a role in mediating expression of M1 markers induced by Carfilzomib in M2 macrophages. BMDMs were activated by IL-4 (20 ng/ml) for 24 h, followed by treatment with IRE1α inhibitors including Kira6, 4μ8c for 1 h, and then stimulated by Carfilzomib (1 μM) for 6 h. Expression of *Il-1β* and *Il-6* was quantified through RT-qPCR. Data are means ± SD of three independent experiments. $**P < 0.01$, $***P < 0.001$ (Student's *t*-test).

I Impact of inhibition of IRE1α kinase activity on the expression of M1 marker genes and ER stress-related genes in Raw264.7 activated by Carfilzomib. Raw264.7 cells were activated with IL-4 (20 ng/ml) for 24 h, followed by treatment with Kira6 for 1 h, and then stimulated by Carfilzomib (1 μM) for 6 h. The mRNA expression of *Bip*, *Chop, Il-1β*, and *Il-6* was quantified through RT-qPCR. Data are means ± SD of three independent experiments. $*P < 0.05$, $**P < 0.01$ (Student's *t*-test).

Source data are available online for this figure.

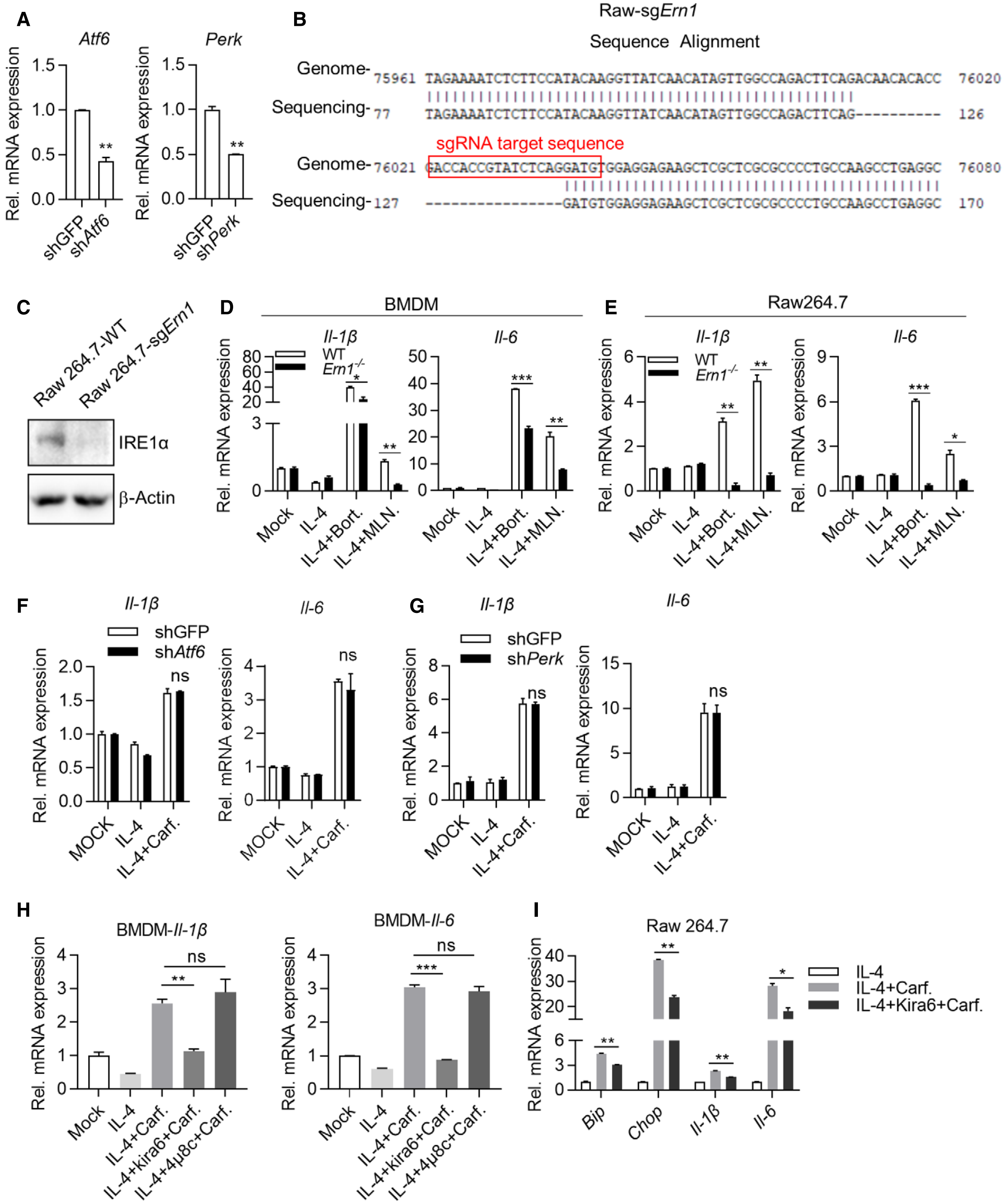

Figure EV3.

© 2021 The Authors

Figure EV4. Carfilzomib shrinks tumor *in vivo* through promoting polarization of M2 macrophage into M1-like macrophages.

A Gating strategy for analyzing the macrophages in blood or lung tissues. After gating the singlets (by FSC-A and SSC-W) and living cells (DAPI negative), macrophages are gated on $CD45^{+}CD11b^{+}$ $F4/80^{high}$ cells.

B Elimination of macrophages by Clodrosome. One day after injection of Clodrosome, orbital blood was collected, followed by red blood cells lysis. $CD45^{+}CD11b^{+}$ $F4/80^{high}$ cells were analyzed by flow cytometry (left panel). Statistics represent the proportion of macrophages (right panel). Data are means ± SD of three independent experiments. ***$P < 0.001$ (Student's t-test).

C Carfilzomib treatment has no remarkable effect on CD86 expression of infiltrating macrophages in tumor. Following protocol shown in Fig 5E, the expression of CD86 was detected by flow cytometry and the statistics represent the proportion of CD86. Data are means ± SD of three independent experiments.

D Impact of Carfilzomib on infiltration of $CD4^{+}$ T cells in lung cancer. Lung cancer-bearing EGFR-TD mice were treated for 2 weeks and then euthanized. Lung tissue was dissected for analysis of the infiltration of $CD4^{+}$ T cells in tumors by flow cytometry (left panel). Statistics for proportion of CD4-positive cells (right panel). Data are means ± SD of three independent experiments.

E Elimination of macrophages by DT in immune-reconstituted TD mice. TD mice were irradiated with X-ray (8 Gy for 20 min) and transplanted with 5×10^{6} of bone marrow cells from CD11b-DTR mice. Immune-reconstituted mice were fed with doxycycline diet for 12 weeks. Lung cancer was documented through CT. Mice were treated for 2 weeks with DT before FACS analysis. $CD11B^{+}$ cells were analyzed by flow cytometry (left panel) and statistics on percentage of macrophages were calculated (right panel). Data are means ± SD of three independent experiments. **$P < 0.01$ (Student's t-test).

F Elimination of macrophages by Clodrosome. Clodronate liposomes of 200 µl were injected intravenously (iv.) every 3 days. Depletion of macrophages ($CD45^{+}CD11b^{+}$ cells) was analyzed in lung cancers (left panel). Statistics on percentage of macrophages are shown (right panel). Data are means ± SD of three independent experiments. **$P < 0.01$ (Student's t-test).

G–I The therapeutic effect of Carfilzomib is largely depending on the presence of macrophages in xenograft model. EG7 cells were inoculated in wild-type C57BL/6 mice. Tumor volume was allowed to reach a volume of around 90 mm^{3}. Mice were treated by saline, Carfilzomib, or Carfilzomib plus Clodrosome for 2 weeks ($n = 5$). Tumor growth was monitored during treatment (G) and the xenografts were dissected to photograph (H) or weigh (I) in the end of experiment. Data are shown as mean ± SD and n indicates the number of biological replicates. *$P < 0.05$, ***$P < 0.001$ (Student's t-test).

J Carfilzomib treatment promotes the infiltration of M1 macrophages. After treatment, xenografts of (H) were harvested to analyze the expression of CD80, CD86, and CD206 by flow cytometry.

K Statistics represent the proportion of CD80-, CD86-, or CD206-positive cells in xenograft. Data are means ± SD of three independent experiments. **$P < 0.01$ (Student's t-test).

L Cytotoxicity of Carfilzomib on EG7 cells. EG7 cells were treated with Carfilzomib for 24 h at indicated concentration. Cell viability was analyzed with CCK8. IC50 (concentration for 50% of maximal effect) of Carfilzomib to repress EG7 cells growth was shown. Data are means ± SD of three independent experiments.

M Macrophages were eliminated in LyzM-cre; LSL-dDTA mice. Orbital blood was collected and red blood cells were disrupted. Then, the $CD45^{+}CD11b^{+}$ $F4/80^{high}$ cells were analyzed by flow cytometry (left panel). Statistics on percentage of macrophages were shown (right panel). Data are means ± SD of three independent experiments. **$P < 0.01$ (Student's t-test).

© 2021 The Authors

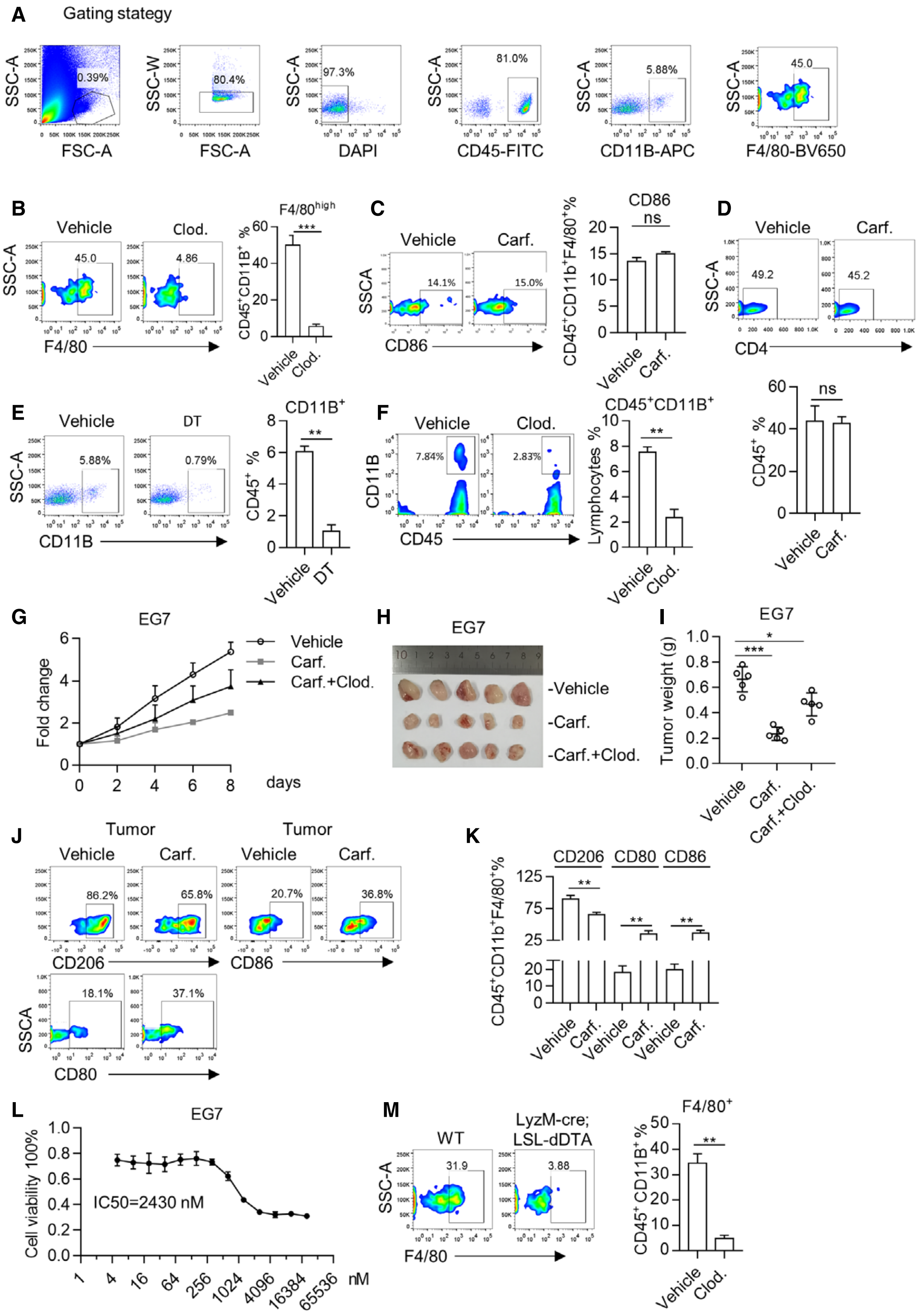

Figure EV4.

 © 2021 The Authors

Figure EV5. Carfilzomib synergizes with PD-1 inhibitors to treat lung cancer.

A Gating strategy was used for analyzing the infiltration of $CD8^+$ or $CD4^+$ T cells in lung tissues. After gating the single (by FSC-A and SSC-W) and living cells (DAPI negative), CD8- or CD4-positive cells were gated on $CD45^+$ cells.

B, C Elimination of $CD8^+$ T cells by CD8α antibody. After 2-week treatment with antibody, orbital blood was collected and red blood cells were lysed. $CD8^+$ T cells were analyzed by flow cytometry (B). Statistics on percentages of CD8-positive cells (C). Data are means ± SD of three independent experiments. $^{**}P < 0.01$ (Student's *t*-test).

D, E Elimination of $CD4^+$ T cells by CD4 antibody. After 2-week treatment of antibody, orbital blood was collected and red blood cells were lysed. $CD4^+$ T cells were analyzed by flow cytometry (D). Statistics on percentage of CD4-positive cells (E). Data are means ± SD of three independent experiments. $^{***}P < 0.001$ (Student's *t*-test).

F–I Statistics on tumor burden of mice before and after treatment. Data are means ± SD of three independent experiments (F, H). Statistics on relative tumor area and $Ki67^+$ cells in sections of lung tissue of mice (G, I). Data are means ± SD of three independent experiments. $^{*}P < 0.05$, $^{**}P < 0.01$, $^{***}P < 0.001$ (Student's *t*-test).

© 2021 The Authors

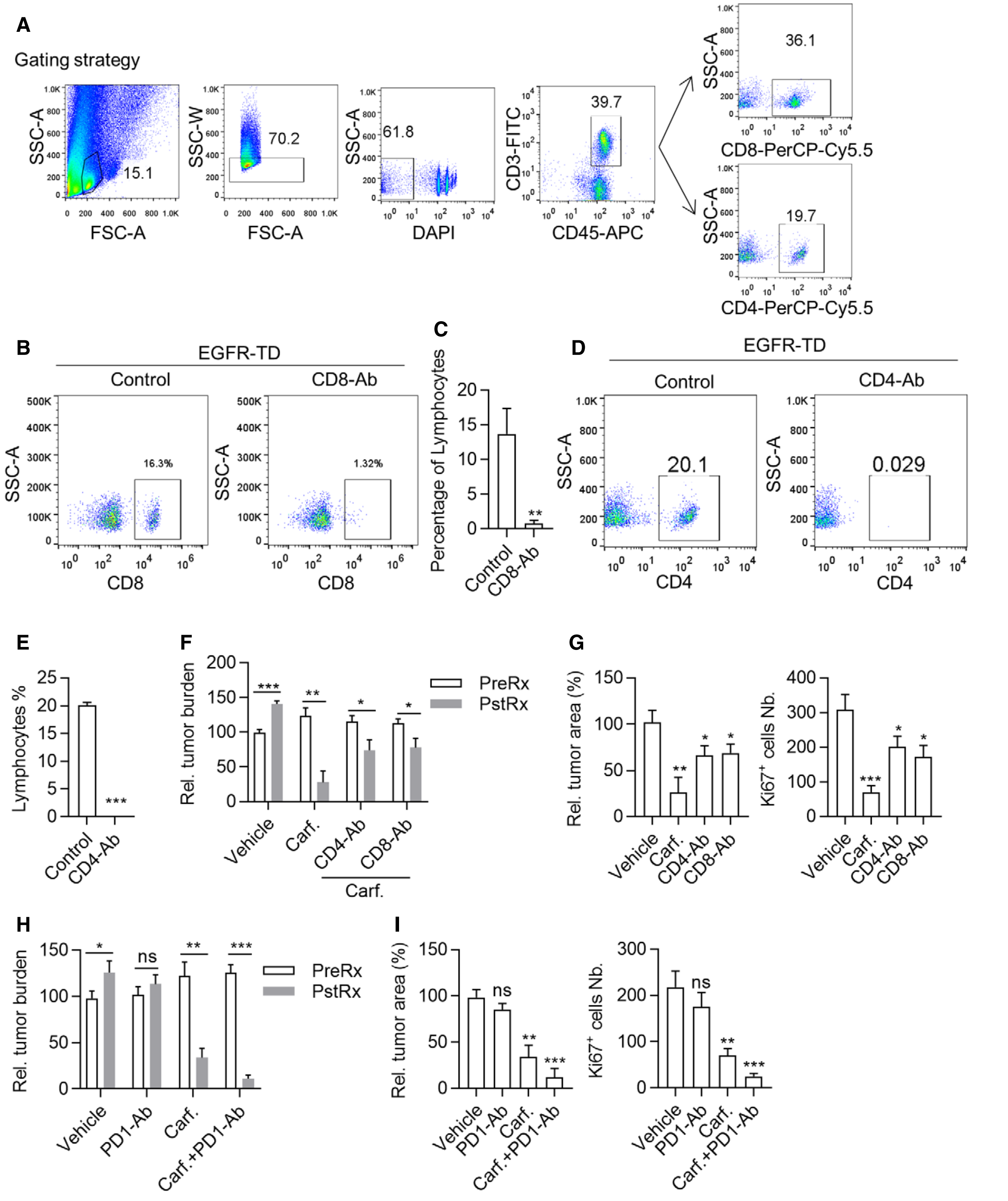

Figure EV5.

© 2021 The Authors
