# Supplementary figures and images for: Carfilzomib modulates tumor microenvironment to potentiate immune checkpoint therapy for cancer
Source: EMBO Mol Med. 2021 Dec 13;14(1):e14502. doi: 10.15252/emmm.202114502 (PMC8749493; doi:10.15252/emmm.202114502)

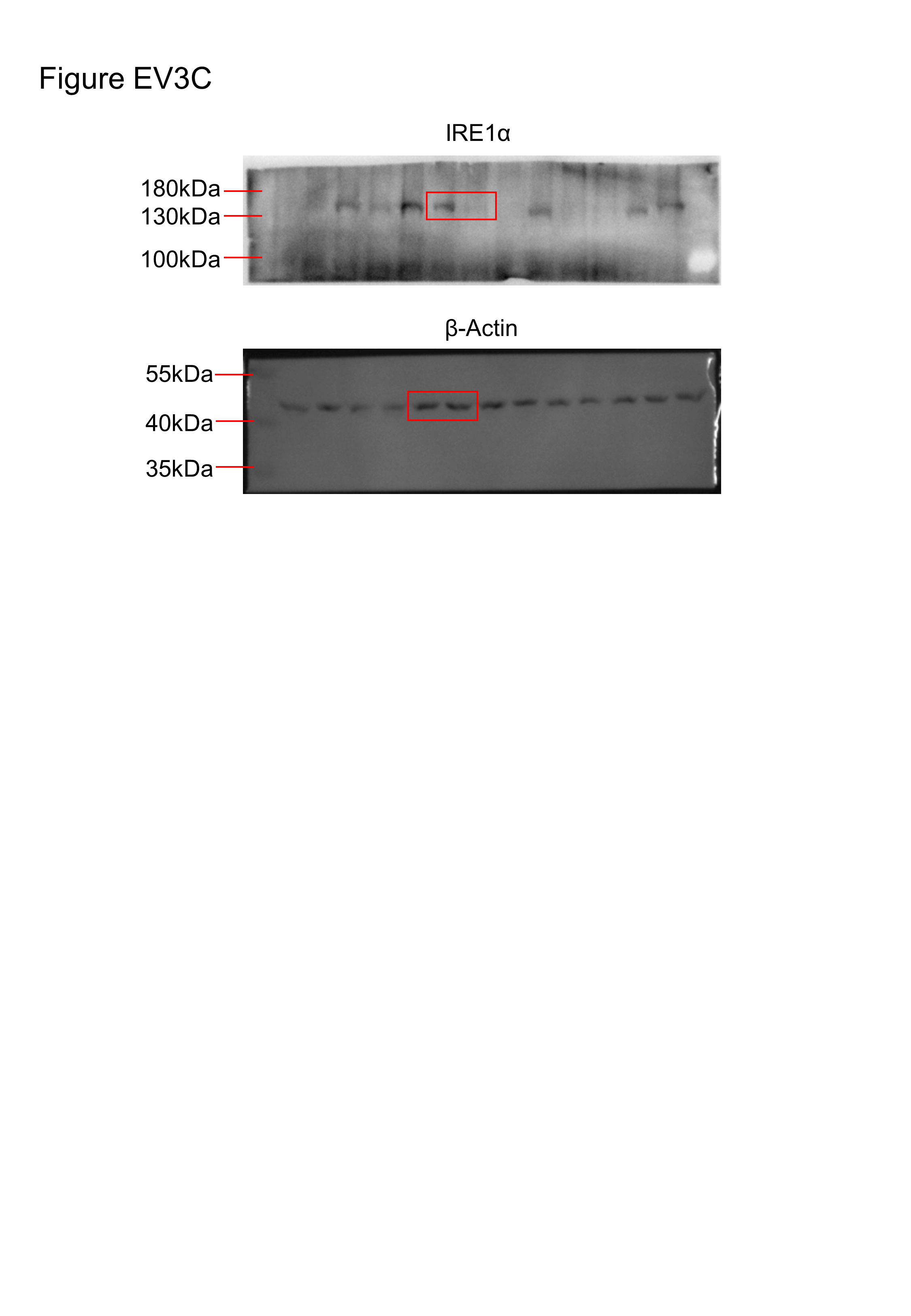

Supplement: Supplementary file 3 — Source Data for Expanded View and Appendix [file EMMM-14-e14502-s006.zip › emmm202114502-sup-0006-SDataEV/manuscript_EMM-2021-14502_SourceDataForFigureEV3/emmm202114502-sup-0005-SDataFigEV3C.TIF]

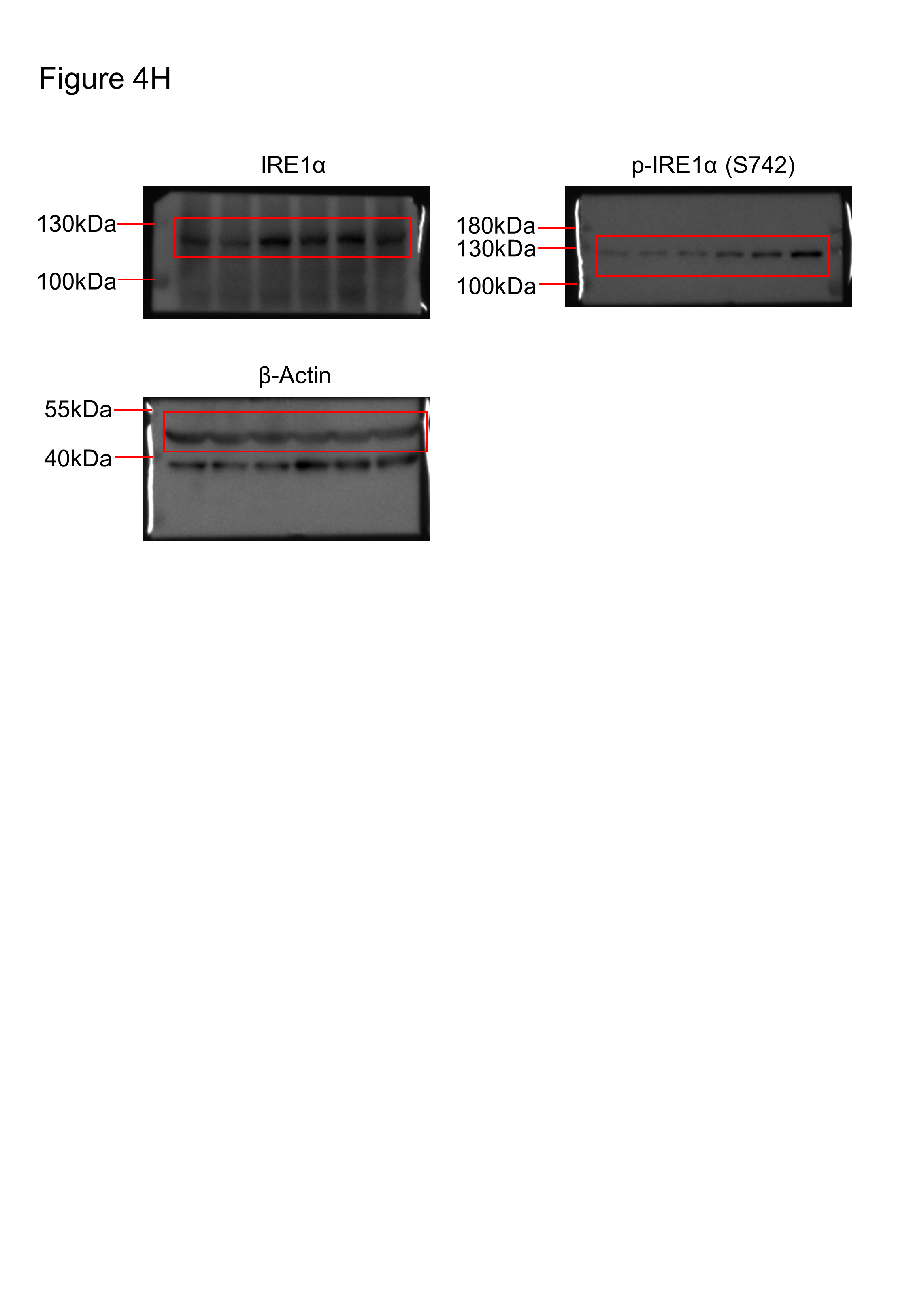

Supplement: Supplementary file 3 — Source Data for Expanded View and Appendix [file EMMM-14-e14502-s006.zip › emmm202114502-sup-0006-SDataEV/manuscript_EMM-2021-14502_SourceDataForFigure_4-1/SourceDataForFigure_4H.TIF]

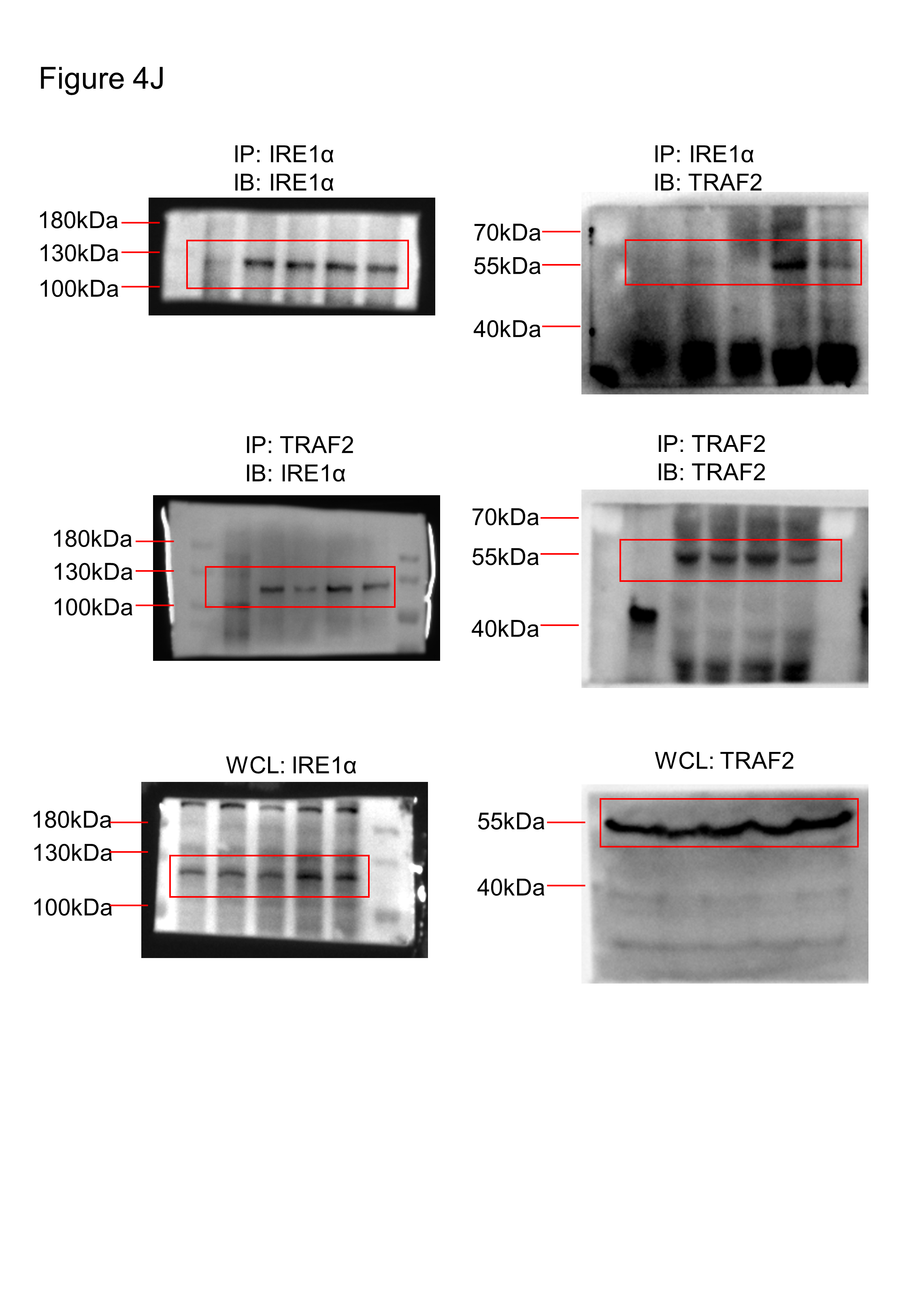

Supplement: Supplementary file 3 — Source Data for Expanded View and Appendix [file EMMM-14-e14502-s006.zip › emmm202114502-sup-0006-SDataEV/manuscript_EMM-2021-14502_SourceDataForFigure_4-1/SourceDataForFigure_4J.TIF]

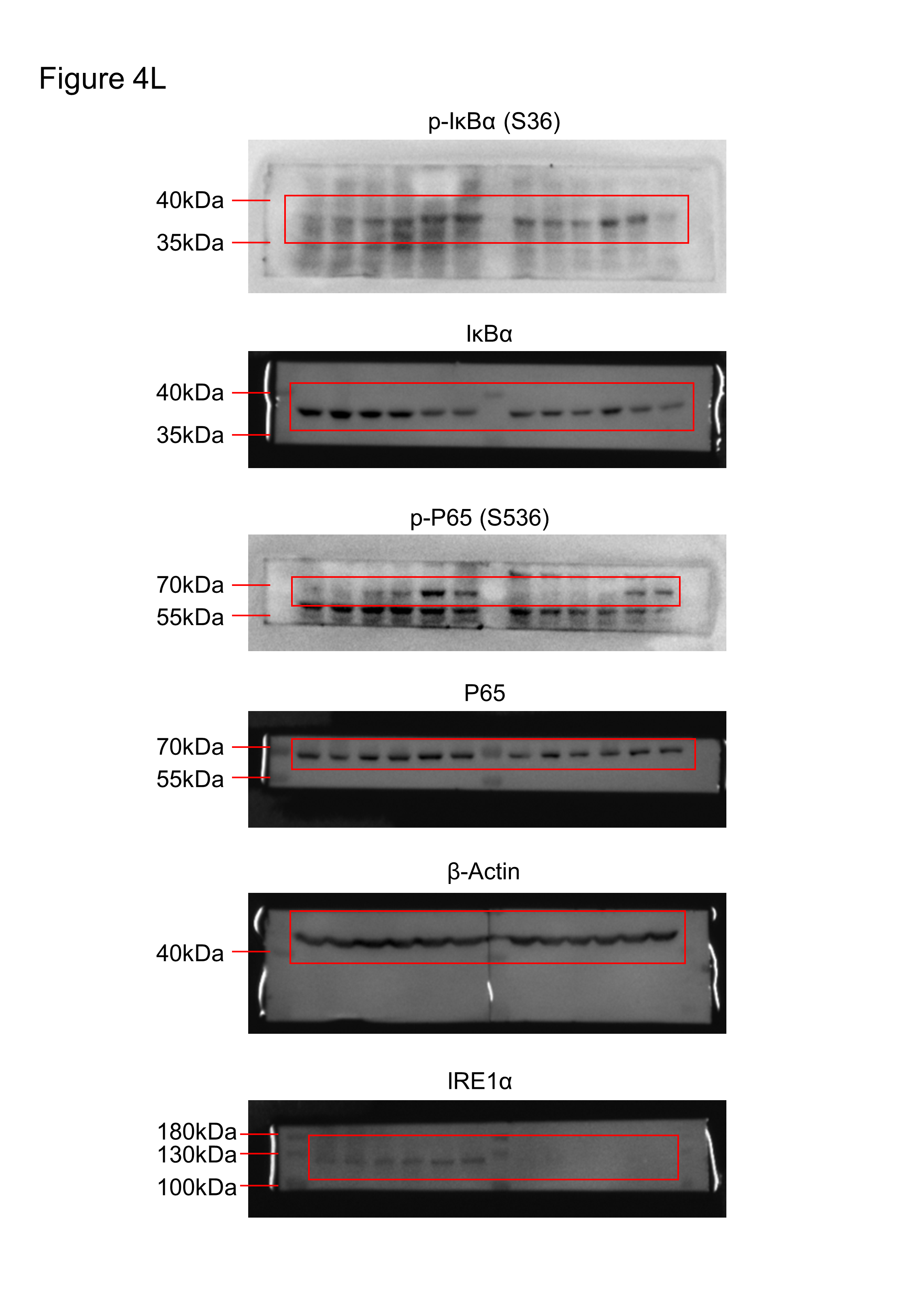

Supplement: Supplementary file 3 — Source Data for Expanded View and Appendix [file EMMM-14-e14502-s006.zip › emmm202114502-sup-0006-SDataEV/manuscript_EMM-2021-14502_SourceDataForFigure_4-1/SourceDataForFigure_4L.TIF]

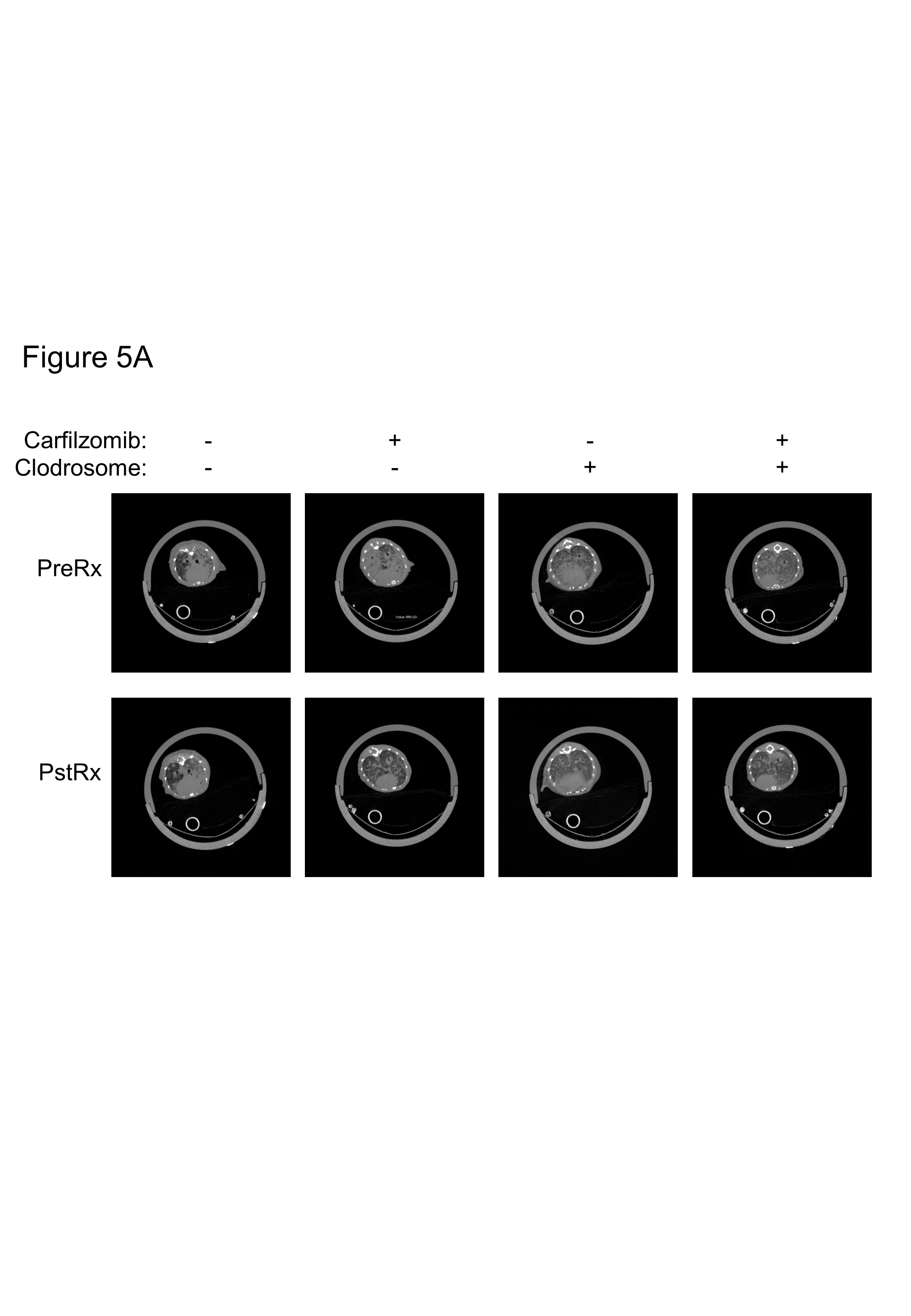

Supplement: Supplementary file 4 — Source Data for Figure 5 [file EMMM-14-e14502-s002.tif]

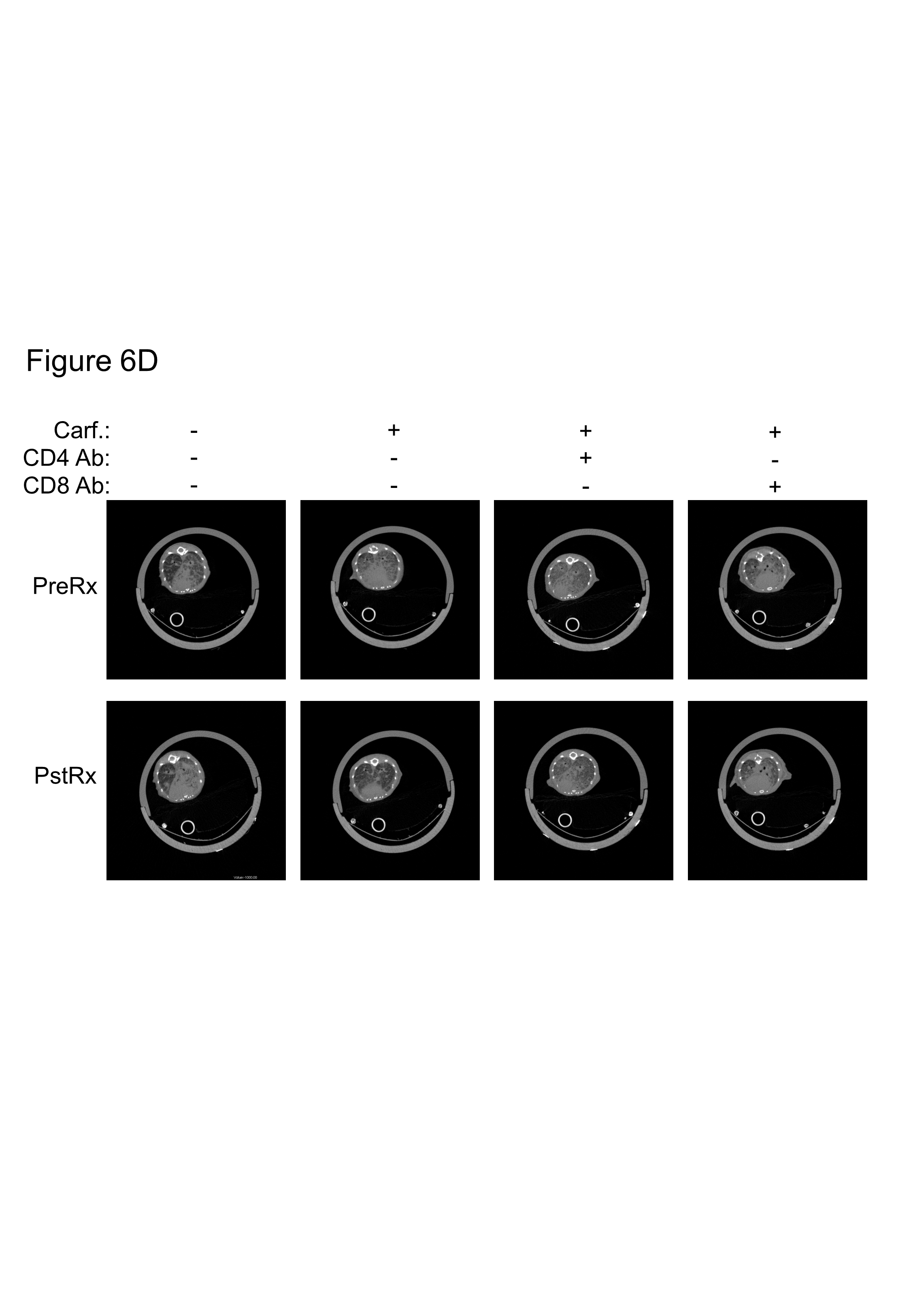

Supplement: Supplementary file 5 — Source Data for Figure 6 [file EMMM-14-e14502-s004.tif]
