# Supplementary material for: Carfilzomib modulates tumor microenvironment to potentiate immune checkpoint therapy for cancer
Source: EMBO Mol Med. 2021 Dec 13;14(1):e14502. doi: 10.15252/emmm.202114502 (PMC8749493; doi:10.15252/emmm.202114502)
Supplement: Supplementary file 6 [file EMMM-14-e14502-s003.pdf]

EMBO Molecular Medicine

Carfilzomib modulates tumor microenvironment to potentiate immune checkpoint therapy for cancer

Qian Zhou, Jinxia Liang, Tong Yang, Jin Liu, Bo Li, Yingchang Li, Zhenzhen Fan, Weida Wang, Wensheng Chen, Sujing Yuan, Meng Xu, Qigui Xu, Zhidong Luan, Zhong-Jun Xia, Penghui Zhou, Yadong Huang, and Liang Chen
DOI: 10.15252/emmm.202114502

Corresponding authors: Liang Chen (chenliang@jnu.edu.cn) , Qian Zhou (zhouqian_whu@163.com)

Review Timeline:

Submission Date:	3rd May 21
Editorial Decision:	27th May 21
Revision Received:	8th Oct 21
Editorial Decision:	18th Oct 21
Revision Received:	31st Oct 21
Accepted:	5th Nov 21

Editor: Jingyi Hou

Transaction Report:

(Note: With the exception of the correction of typographical or spelling errors that could be a source of ambiguity, letters and reports are not edited. Depending on transfer agreements, referee reports obtained elsewhere may or may not be included in this compilation. Referee reports are anonymous unless the Referee chooses to sign their reports.)

27th May 2021

Thank you again for submitting your work to EMBO Molecular Medicine. We have now heard back from the three referees who evaluated your manuscript. As you will see from the reports below, the referees acknowledge the interest and novelty of the study. However, they also raise a series of concerns about your work, which should be convincingly addressed in a major revision of the present manuscript.

The referees' recommendations are rather clear, and there is no need to reiterate their comments. Most of their concerns refer to the need to provide further details, clarifications, and controls, and to improve the presentation of the study in order to make the data and the main conclusions easily accessible to the general readers.

We would welcome the submission of a revised version within three months for further consideration. Please note that EMBO Molecular Medicine strongly supports a single round of revision. As acceptance or rejection of the manuscript will depend on another round of review, your responses should be as complete as possible.

EMBO Molecular Medicine has a "scooping protection" policy, whereby similar findings that are published by others during review or revision are not a criterion for rejection. Should you decide to submit a revised version, I do ask that you get in touch after three months if you have not completed it, to update us on the status.

We are aware that many laboratories cannot function at full efficiency during the current COVID-19/SARS-CoV-2 pandemic and have therefore extended our "scooping protection policy" to cover the period required for a full revision to address the experimental issues. Please let me know should you need additional time, and also if you see a paper with related content published elsewhere.

Please read below for important editorial formatting and consult our author's guidelines for proper formatting of your revised article for EMBO Molecular Medicine.

I look forward to receiving your revised manuscript.

*****

When submitting your revised manuscript, please carefully review the instructions that follow below. We perform an initial quality control of all revised manuscripts before re-review; failure to include requested items will delay the evaluation of your revision.

We require:

1) A .docx formatted version of the manuscript text (including legends for main figures, EV figures and tables). Please make sure that the changes are highlighted to be clearly visible.

2) Individual production quality figure files as .eps, .tif, .jpg (one file per figure). For guidance, download the 'Figure Guide PDF' (https://www.embopress.org/page/journal/17574684/authorguide#figureformat).

3) A .docx formatted letter INCLUDING the reviewers' reports and your detailed point-by-point responses to their comments. As part of the EMBO Press transparent editorial process, the point-by-point response is part of the Review Process File (RPF), which will be published alongside your paper.

4) A complete author checklist, which you can download from our author guidelines (https://www.embopress.org/page/journal/17574684/authorguide#submissionofrevisions). Please insert information in the

checklist that is also reflected in the manuscript. The completed author checklist will also be part of the RPF.

5) Please note that all corresponding authors are required to supply an ORCID ID for their name upon submission of a revised manuscript.

6) It is mandatory to include a 'Data Availability' section after the Materials and Methods. Before submitting your revision, primary datasets produced in this study need to be deposited in an appropriate public database, and the accession numbers and database listed under 'Data Availability'. Please remember to provide a reviewer password if the datasets are not yet public (see https://www.embopress.org/page/journal/17574684/authorguide#dataavailability).

In case you have no data that requires deposition in a public database, please state so in this section. Note that the Data Availability Section is restricted to new primary data that are part of this study.

7) For data quantification: please specify the name of the statistical test used to generate error bars and P values, the number (n) of independent experiments (specify technical or biological replicates) underlying each data point and the test used to calculate p-values in each figure legend. The figure legends should contain a basic description of n, P and the test applied. Graphs must include a description of the bars and the error bars (s.d., s.e.m.).

8) We would also encourage you to include the source data for figure panels that show essential data. Numerical data should be provided as individual .xls or .csv files (including a tab describing the data). For blots or microscopy, uncropped images should be submitted (using a zip archive if multiple images need to be supplied for one panel). Additional information on source data and instruction on how to label the files are available at

.

9) Our journal encourages inclusion of *data citations in the reference list* to directly cite datasets that were re-used and obtained from public databases. Data citations in the article text are distinct from normal bibliographical citations and should directly link to the database records from which the data can be accessed. In the main text, data citations are formatted as follows: "Data ref: Smith et al, 2001" or "Data ref: NCBI Sequence Read Archive PRJNA342805, 2017". In the Reference list, data citations must be labeled with "[DATASET]". A data reference must provide the database name, accession number/identifiers and a resolvable link to the landing page from which the data can be accessed at the end of the reference. Further instructions are available at .

10) We replaced Supplementary Information with Expanded View (EV) Figures and Tables that are collapsible/expandable online. A maximum of 5 EV Figures can be typeset. EV Figures should be cited as 'Figure EV1, Figure EV2" etc... in the text and their respective legends should be included in the main text after the legends of regular figures.

- For the figures that you do NOT wish to display as Expanded View figures, they should be bundled together with their legends in a single PDF file called *Appendix*, which should start with a short Table of Content. Appendix figures should be referred to in the main text as: "Appendix Figure S1, Appendix Figure S2" etc.

- Additional Tables/Datasets should be labeled and referred to as Table EV1, Dataset EV1, etc. Legends have to be provided in a separate tab in case of .xls files. Alternatively, the legend can be supplied as a separate text file (README) and zipped together with the Table/Dataset file.

See detailed instructions here:

.

11) The paper explained: EMBO Molecular Medicine articles are accompanied by a summary of the articles to emphasize the major findings in the paper and their medical implications for the non-specialist reader. Please provide a draft summary of your article highlighting

- the medical issue you are addressing,

- the results obtained and

- their clinical impact.

This may be edited to ensure that readers understand the significance and context of the research. Please refer to any of our published articles for an example.

12) For more information: There is space at the end of each article to list relevant web links for further consultation by our

readers. Could you identify some relevant ones and provide such information as well? Some examples are patient associations, relevant databases, OMIM/proteins/genes links, author's websites, etc...

13) Author contributions: the contribution of every author must be detailed in a separate section (before the acknowledgments).

14) A Conflict of Interest statement should be provided in the main text.

15) Every published paper now includes a 'Synopsis' to further enhance discoverability. Synopses are displayed on the journal webpage and are freely accessible to all readers. They include a short stand first (maximum of 300 characters, including space) as well as 2-5 one-sentences bullet points that summarizes the paper. Please write the bullet points to summarize the key NEW findings. They should be designed to be complementary to the abstract - i.e. not repeat the same text. We encourage inclusion of key acronyms and quantitative information (maximum of 30 words / bullet point). Please use the passive voice. Please attach these in a separate file or send them by email, we will incorporate them accordingly.

Please also suggest a striking image or visual abstract to illustrate your article as a PNG file 550 px wide x 300-600 px high.

EMBO Molecular Medicine has a "scooping protection" policy, whereby similar findings that are published by others during review or revision are not a criterion for rejection. Should you decide to submit a revised version, I do ask that you get in touch after three months if you have not completed it, to update us on the status.

***** Reviewer's comments *****

Referee #1 (Comments on Novelty/Model System for Author):

The comments and advice are listed in the remarks to the authors.

Referee #1 (Remarks for Author):

The authors have screened FDA-approved drugs and found that Carfilzomib drove IL-4 induced macrophages to express M1 cytokines, enhanced phagocytosis ability and promoted T cell proliferation. In vivo, Carfilzomib reprogrammed TAMs into M1-like macrophages, and promoted CD8+ T cell proliferation or activation to inhibit autochthonous lung cancers in a mouse model. And Carfilzomib synergized with PD-1 antibody to better control autochthonous lung cancers. They further identified that Carfilzomib activated IRE1α to recruit TRAF2, resulting in the activated NF-κB to induce expression of the proinflammatory cytokines. In general, this paper have interesting and important findings, which has proposed that the FDA-approved Carfilzomib can be combined together with anti-PD-1 antibody to improve immune therapy against EGFR mutant-lung cancer. This manuscript can be considered for publication and a revision is encouraged after the authors address the below concerns.
My questions are listed below.
1. Fig. 1: In the mock sample, can Carfilzomib alone affect macrophage survival or expression of these proinflammatory cytokines?
2. Fig. 2: Can Carfilzomib alone affect T cell proliferation driven by anti-CD3/CD28 stimulation?
Can Carfilzomib affect the expression levels of MHC-I and MHC-II and CD80 in macrophages, which are critical for antigen presentation? In Fig.2, CD86 levels were checked, while in the in vivo study shown in Fig. 6, CD80 expression was measured. The authors should include both CD80 and CD86 in Fig. 2 and Fig. 6.
The effector on phagocytosis is enhanced by Carfilzomib treatment from 1% to 3%. This might be due to the limited sensitivity of the assay. Nevertheless, this change is not substantial. The authors are suggested to delete the strong description such as significantly. Similarly, words like "drastic" in the manuscript should be modified.
3. Fig. 4J: Carfilzomib treatment can increase TRAF2 binding to IRE1α. But in this figure, the reduced amount of IRE1α is shown after Carfilzomib treatment. IS this correct?
Fig. 4A The Kira6 effect is shown differently in RAW264.7 (completely) vs BMDM (partially). This should be discussed.
4. Fig. S5C: The in vivo study did not observe difference of CD4+ T cells. However, the ex vivo data in Fig. 2 indeed show that Carfilzomib treatment enhances both CD8+ T cell and CD4+ T cell proliferation. This should be discussed.
5. The major question: The IRE1α-TRAF2 module can explain that Carfilzomib treatment enhances expression of the proinflammatory cytokines in IL-4 induced M2 macrophages. But what is the reason to explain the reduced CD206 or Arg1 expression in these cells?

Referee #2 (Comments on Novelty/Model System for Author):

The manuscript by Zhou et al presents a novel approach to increase the efficiency of PD-1 ICB in resistant cancer. They utilize a variety of experimental procedures and convincingly prove their points. The conclusions are both new and of high medical

impact.

Referee #2 (Remarks for Author):

The manuscript by Zhou et al presents a novel approach to increase the efficiency of PD-1 ICB in resistant cancer. They utilize a variety of experimental procedures and convincingly prove their points. The conclusions are both new and of high medical impact.

Qian Zhou and her colleagues use a smart screening system to identify approved drugs, that may transform M2 to M1 macrophages. They identified Carfilzomib, together with two other proteasome inhibitors and then meticulously work their way through its biological effects, its ability to render TAM inflammatory, the mode of action and the intracellular signaling involved. Then they address the effects of Carfilzomib in vivo in an autochthonous murine lung cancer model and show impressive results, especially in combination with a PD-1 inhibitor. Finally, they show similar effects on human ex vivo differentiated macrophages, which strongly argues for transferability to human application. Hence the combination of proteasome inhibitors my represent a new combination treatment to help all those cancer patients who are unresponsive to PD-1 checkpoint blockade. The only prerequisite would be a sufficient TAM infiltration of their tumor. The authors performed their experiments well examined their assumptions often with different methods. Hence I consider this a high quality paper from the scientific point of view. Unfortunately, the English language is, in many parts, not sufficient for publication.
Minor points:
1) I really advise the authors to have the manuscript corrected by a native speaker or a commercial service for language and grammar.
2) In some parts the manuscript is also quite demanding for readers, who are not specialist to the field, so a bit more explanation on experimental systems in the results section would be helpful for the general readership.
3) Especially the model system of tumor-SN-treated Raw264.7 needs a (short) introduction.

Referee #3 (Comments on Novelty/Model System for Author):

Overall, this manuscript by Zhou et al is interesting and timely. The authors identified a way to reprogram immunosuppressive M2 macrophages toward antitumor M1 cells, which may help to develop a novel immunotherapeutic strategy. The experiments are in general informative and reasonably well designed. However, several concerns on the analysis, presentation and interpretation of data are raised (see below).

Referee #3 (Remarks for Author):

In this manuscript, Zhou et al found that carfilzomib, a proteasome inhibitor (PI), could drive murine and human M2 macrophages to partially exhibit M1-like phenotype and function. By screening FDA-approved drugs, the authors identified three PIs namely carfilzomib, bortezomib and MLN9708 that effectively elicited IL-1β expression in murine bone marrow-derived macrophages (BMDMs). Further examination revealed that carfilzomib-induced M1-like cells (Ci-M1). Importantly, Ci-M1 exhibited enhanced phagocytotic and antigen-presenting activity, suggesting that carfilzomib treatment at least partially endowed M2/TAM with phenotype and functions similar to M1 macrophages. Investigation into the underlying mechanism of carfilzomib-induced M2 transformation into M1-like cells indicated that this process was dependent on an IRE1α-TRAF2-NF-κB pathway. Therapeutically, systemic carfilzomib treatment could inhibit tumor growth and synergize with PD-1 inhibitors in vivo, an effect that was attenuated in the absence of macrophages.
Overall, reprogramming immunosuppressive/protumor macrophages toward cells with antitumor function is emerging as an attractive notion. The present study is interesting and timely. The experiments are in general informative and reasonably well designed. I have some minor concerns about the analysis, presentation and interpretation of data.
1) Figure 1B: Three red dots are shown but they are not individually and specifically annotated. In addition, what does the x axis stand for? It is also unclear whether carfilzomib, bortezomib and MLN9708 are the only drugs in the library that were capable of activating IL-1β expression in IL4-induced M2 macrophages. Is there other PIs in the library that were not able to induce IL-1β expression? These issues should be clarified.
2) Figure 2A: Although carfilzomib could decrease CD206 and arginase 1 expression, this reduction was marginal. Are the levels of IL-6 and iNOS expression post carfilzomib treatment comparable to that in conventional M1 macrophages? A positive control is recommended for supporting the authors' claim that carfilzomib could reprogram M2 macrophage into M1-like population.
3) Figure 2C: The gating strategy is unclear. How was the CD86+ and CD206+ population defined? Was it based on isotype-matched antibody staining or fluorescence minus one (FMO) control? This point is also applicable for other flow cytometric plots shown in the manuscript (e.g., Figure S2E and S2L).
4) Figure 2F and 2H: Both assays showed that carfilzomib could enhance macrophage phagocytotic ability, but the phagocytosis efficiencies exhibit a ten-fold difference. This should be clarified.
5) Figure 2L, y axis: Is it CD8+ % or CD4+ %? Figure 3D: Extra asterisks are shown on IL-6.
6) Figure 4: It is very interesting that the kinase activity of IRE1α, instead of the endoribonuclease activity and the activation of XBP1, mediated the carfilzomib induced reprograming of M2 macrophages. The authors are encouraged to discuss the impact of PIs and ER stress inducers on XBP1-deficienct cells.

***** Reviewer's comments *****

Referee #1 (Comments on Novelty/Model System for Author):

The comments and advice are listed in the remarks to the authors.

Referee #1 (Remarks for Author):

The authors have screened FDA-approved drugs and found that Carfilzomib drove IL-4 induced macrophages to express M1 cytokines, enhanced phagocytosis ability and promoted T cell proliferation. In vivo, Carfilzomib reprogrammed TAMs into M1-like macrophages, and promoted CD8+ T cell proliferation or activation to inhibit autochthonous lung cancers in a mouse model. And Carfilzomib synergized with PD-1 antibody to better control autochthonous lung cancers. They further identified that Carfilzomib activated IRE1α to recruit TRAF2, resulting in the activated NF-κB to induce expression of the proinflammatory cytokines. In general, this paper have interesting and important findings, which has proposed that the FDA-approved Carfilzomib can be combined together with anti-PD-1 antibody to improve immune therapy against EGFR mutant-lung cancer. This manuscript can be considered for publication and a revision is encouraged after the authors address the below concerns.

Reply: We thank Reviewer for finding our work interesting and important.

My questions are listed below.

1. Fig. 1: In the mock sample, can Carfilzomib alone affect macrophage survival or expression of these proinflammatory cytokines?

Reply: Following Reviewer's suggestion, we have now checked macrophage survival and expression of proinflammatory cytokines (IL-6 and INOS) after Carfilzomib treatment. We found that Carfilzomib slightly inhibited the proliferation of macrophages after being treated for 12 hours, and did not affect macrophage cell viability in 6 hours (Figure 1 for Reviewer). Meanwhile, Carfilzomib alone was able to induce *Il-1β*, *Il-6* and *Inos* expression in macrophage (see new Figure EV2E).

Figure 1 for Reviewer

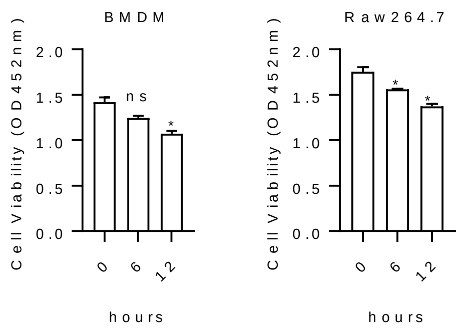

Figure 1 for Reviewer: BMDMs or Raw264.7 were treated by Carfilzomib (500 nM) for 6 hours or 12 hours (0 hour means beginning of DMSO administration) and CCK8 assay kit was applied to estimate cell viability.

2. Fig. 2: Can Carfilzomib alone affect T cell proliferation driven by anti-CD3/CD28 stimulation? Can Carfilzomib affect the expression levels of MHC-I and MHC-II and CD80 in macrophages, which are critical for antigen presentation? In Fig.2, CD86 levels were checked, while in the in vivo study shown in Fig. 6, CD80 expression was measured. The authors should include both CD80 and CD86 in Fig. 2 and Fig. 6.

The effector on phagocytosis is enhanced by Carfilzomib treatment from 1% to 3%. This might be due to the limited sensitivity of the assay. Nevertheless, this change is not substantial. The authors are suggested to delete the strong description such as significantly. Similarly, words like "drastic" in the manuscript should be modified.

Reply: Thanks so much for these great suggestions. We analyzed T cell proliferation driven by anti-CD3/CD28 stimulation after Carfilzomib treatment. The results indicated that Carfilzomib did not affect CD4 and CD8 T cell proliferation (see new Figure EV2Q & EV2R). We also checked the expression levels of MHC-I, MHC-II and CD80 in macrophages in Ci-M1. We found that Ci-M1 expressed higher levels of MHC-II and CD80 but not MHC-I in comparison to IL-4-activated M2 macrophages (new Figure 2C & 2D & EV2M & EV2N). Besides, both CD80 and CD86 are included in new Fig. 2 and Fig. 5. Following Reviewer's suggestions, we have now corrected the statements throughout the manuscript. We have now deleted "dramatically" or used "markedly" instead of "dramatically".

3. Fig. 4J: Carfilzomib treatment can increase TRAF2 binding to IRE1α. But in this figure, the reduced amount of IRE1α is shown after Carfilzomib treatment. Is this correct?

Reply: Thanks so much for bringing this important issue into our attention. We repeated this experiment. The results indicated that expression of IRE1α and the recruitment of TRAF2 by IRE1α were significantly increased and in Ci-M1 (New Figure 4J).

Fig. 4A The Kira6 effect is shown differently in RAW264.7 (completely) vs BMDM (partially). This should be discussed.

Reply: We are sorry for not presenting our result clearly enough. Figure 4A showed that IRE1a knockout severely inhibited expression of IL-1B and IL-6 in BMDM. Figure 4B showed that this inhibition is much milder in RAW264.7 cells.

The mechanisms underlying different sensitivity of BMDM and RAW264.7 could be complex. We are not sure of it. But several different mechanisms have been reported to control IL-1B transcription, including transcription factors like Spi-1/PU.1 (Mol Cell Biol 15(1) (1995) 58–68), NF-κB plus C/EBPβ and HIF-1α plus C/EBPβ (J Immunol May 1, 2016, 196 (1 Supplement) 189.14), and epigenetic modifications of its promoter region (Arthritis Rheum. 2009 Nov; 60(11): 3303–3313). The

RAW264.7 cell line was established from a tumor induced by the Abelson murine leukemia virus (Ralph P et al., 1977, J Immunol), which was different from BMDM. Therefore, knockout of IRE1a in RAW264.7 and BMDM, different expression of IL-1B and IL-6, which could be reasonably possible. We have discussed in the page17, line 432-442.

4. Fig. S5C: The in vivo study did not observe difference of CD4+ T cells. However, the ex vivo data in Fig. 2 indeed show that Carfilzomib treatment enhances both CD8+ T cell and CD4+ T cell proliferation. This should be discussed.

Reply: We thank Reviewer for bringing this important issue to our attention. Yes, we saw that peptide-loaded Ci-M1 enhance proliferation of both CD4 and CD8 T cells. However, in Carfilzomib treated lung cancer mouse models, we detected similar amount of CD4 T cells in tumors. We guess that the discrepancy between in vitro and in vivo data could be explained by the complex process of maintaining the homeostasis in vivo. Several factors could affect apparent number of CD4 T cells in tumor: recruitment (CXCL9/10), proliferation (IL-15) and death (IL-2) (J Clin Immunol 2002 Mar;22(2):51-6; Blood 2012 Nov 15;120(20):4246-55.). If the microenvironment of Carfilzomib treated lung cancers release less chemoattractant, fewer CD4 T cells are recruited into the tumor locus. However, the faster proliferation of CD4 T cells could compensate for the lower amount of recruited seed CD4 T cells. Or it could be that CD4 T cells underwent faster apoptosis after proliferation. We thank Reviewer again for pointing out his important issue. We have discussed in the page19, line 485-496.

5. The major question: The IRE1α-TRAF2 module can explain that Carfilzomib treatment enhances expression of the proinflammatory cytokines in IL-4 induced M2 macrophages. But what is the reason to explain the reduced CD206 or Arg1 expression in these cells?

Reply: Thanks so much for this insightful comment. In our opinion, IRE1α-TRAF2 activate NF-κB activity, which is responsible for transcribing proinflammatory

cytokines. This effect could explain that effect of Carfilzomib on expression of proinflammatory cytokines is direct. We guess the effect of Carfilzomib on CD206 and Arg1 is indirect, most probably because Carfilzomib can induce M1 polarization. Indeed, impact of M1 polarization on the expression of M2-related genes have been reported. The negative regulation of M2 genes by M1 polarizing signals are relatively easier to understand. For example, Btk activated by LPS inhibits M2 genes by macrophages (Plos One 9, e85834.); NO produced during M1 polarization inhibits macrophages to transcribe IL-10 gene (Blood 117, 5092-5101). On the contrary, M1 polarization can feedback to upregulate M2 related gene in macrophages. For example: M1 macrophages feature activated glycolysis (Immunity 2015. 42: 419–430); Latic acid, the intermediate metabolite of glycolysis, has been found to promote expression of M2-related genes (Cell Commun Signal. 2018;16(1):54). Therefore, the impact of Carfilzomib on expression of M2-related genes (like CD206 and Arg1) is indirect and more environment-dependent. We have discussed this issue in detail in Page 16-17, line 413-431.

Referee #2 (Comments on Novelty/Model System for Author):

The manuscript by Zhou et al presents a novel approach to increase the efficiency of PD-1 ICB in resistant cancer. They utilize a variety of experimental procedures and convincingly prove their points. The conclusions are both new and of high medical impact.

Referee #2 (Remarks for Author):

The manuscript by Zhou et al presents a novel approach to increase the efficiency of PD-1 ICB in resistant cancer. They utilize a variety of experimental procedures and convincingly prove their points. The conclusions are both new and of high medical impact.

Qian Zhou and her colleagues use a smart screening system to identify approved drugs, that may transform M2 to M1 macrophages. They identified Carfilzomib, together with two other proteasome inhibitors and then meticulously work their way

through its biological effects, its ability to render TAM inflammatory, the mode of action and the intracellular signaling involved. Then they address the effects of Carfilzomib in vivo in an autochthonous murine lung cancer model and show impressive results, especially in combination with a PD-1 inhibitor. Finally, they show similar effects on human ex vivo differentiated macrophages, which strongly argues for transferability to human application. Hence the combination of proteasome inhibitors my represent a new combination treatment to help all those cancer patients who are unresponsive to PD-1 checkpoint blockade. The only prerequisite would be a sufficient TAM infiltration of their tumor. The authors performed their experiments well examined their assumptions often with different methods. Hence, I consider this a high quality paper from the scientific point of view. Unfortunately, the English language is, in many parts, not sufficient for publication.

Reply: We thank Reviewer for finding our study novel and compelling.

Minor points:

1) I really advise the authors to have the manuscript corrected by a native speaker or a commercial service for language and grammar.

Reply: Thanks so much for your suggestions. We have a native English-speaking colleague edited the language of our manuscript. I hope that the current version is ready for publishing.

2) In some parts the manuscript is also quite demanding for readers, who are not specialist to the field, so a bit more explanation on experimental systems in the results section would be helpful for the general readership.

Reply: We thank Reviewer for pointing this out to us. We have explained our rationale for designing our experiments before going into results. We hope that our current version is more friendly to broader readership.

3) Especially the model system of tumor-SN-treated Raw264.7 needs a (short) introduction.

Reply: We thank Reviewer for bringing this point to our attention. We have now added a short introduction of the model system of tumor-SN-treated Raw264.7 (page9, line196-198).

Referee #3 (Comments on Novelty/Model System for Author):

Overall, this manuscript by Zhou et al is interesting and timely. The authors identified a way to reprogram immunosuppressive M2 macrophages toward antitumor M1 cells, which may help to develop a novel immunotherapeutic strategy. The experiments are in general informative and reasonably well designed. However, several concerns on the analysis, presentation and interpretation of data are raised (see below).

Reply: We thank Reviewer for the nice comments on our study.

Referee #3 (Remarks for Author):

In this manuscript, Zhou et al found that carfilzomib, a proteasome inhibitor (PI), could drive murine and human M2 macrophages to partially exhibit M1-like phenotype and function. By screening FDA-approved drugs, the authors identified three PIs namely carfilzomib, bortezomib and MLN9708 that effectively elicited IL-1β expression in murine bone marrow-derived macrophages (BMDMs). Further examination revealed that carfilzomib-induced M1-like cells (Ci-M1). Importantly, Ci-M1 exhibited enhanced phagocytotic and antigen-presenting activity, suggesting that carfilzomib treatment at least partially endowed M2/TAM with phenotype and functions similar to M1 macrophages. Investigation into the underlying mechanism of carfilzomib-induced M2 transformation into M1-like cells indicated that this process was dependent on an IRE1α-TRAF2-NF-κB pathway. Therapeutically, systemic carfilzomib treatment could inhibit tumor growth and synergize with PD-1 inhibitors in vivo, an effect that was attenuated in the absence of macrophages.

Overall, reprogramming immunosuppressive/protumor macrophages toward cells with antitumor function is emerging as an attractive notion. The present study is

interesting and timely. The experiments are in general informative and reasonably well designed. I have some minor concerns about the analysis, presentation and interpretation of data.

1) Figure 1B: Three red dots are shown but they are not individually and specifically annotated. In addition, what does the x axis stand for? It is also unclear whether carfilzomib, bortezomib and MLN9708 are the only drugs in the library that were capable of activating IL-1β expression in IL4-induced M2 macrophages. Is there other PIs in the library that were not able to induce IL-1β expression? These issues should be clarified.

Reply: Following Reviewer's suggestion, we have now annotated these three dots separately (New Figure 1B). We are sorry to make Reviewer confused. The X axis stands for the code of the drugs. We have now added the information in figure legend (Page 34, Line 950).

We checked our library carefully again. We confirmed that there are only three PIs in our library. During our screening, we found only Carfilzomib, Bortezomib and MLN9708 could activate IL-1β expression in IL4-induced M2 macrophages. We have now clarified this issue in the text (page 9, line 212).

2) Figure 2A: Although carfilzomib could decrease CD206 and arginase 1 expression, this reduction was marginal. Are the levels of IL-6 and iNOS expression post carfilzomib treatment comparable to that in conventional M1 macrophages? A positive control is recommended for supporting the authors' claim that carfilzomib could reprogram M2 macrophage into M1-like population.

Reply: Following the reviewers' suggestion, we compared the levels of *Il-6* and *Inos* in Ci-M1 and conventional M1 macrophages (Figure 2a for Reviewer). The results indicated that Ci-M1 expressed lower levels of *Il-6* and *Inos* compare with conventional M1 macrophages (LPS induced macrophage).

Chloroquine (CQ), a proven anti-malarial drug, could switch TAMs from M2 to M1 phenotype (Nature Communications (2018) 9: 873). Following the reviewers'

suggestion, we confirmed that CQ and Carfilzomib could reprogram M2 macrophage into M1-like population (Figure 2b for Reviewer). Both CQ and Carfilzomib can promote the expression of *Il-6* and *Inos*, Carfilzomib being more potent.

Figure 2a for Reviewer

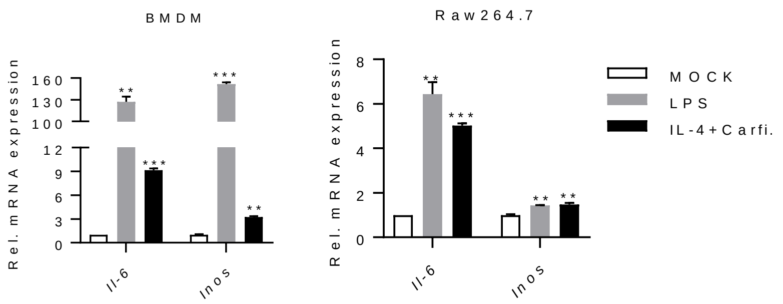

Figure 2b for Reviewer

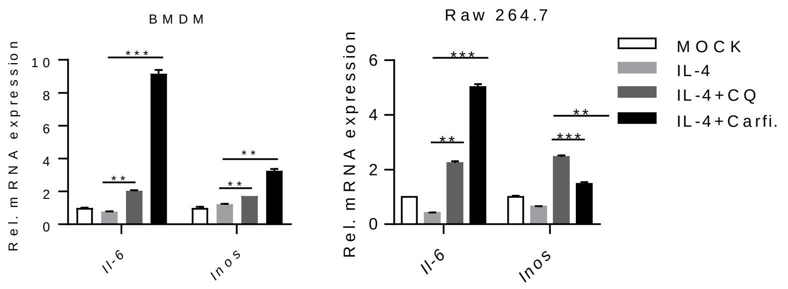

Figure 2 for Reviewer: (a) BMDMs/Raw264.7 cells were treated by LPS to transform into conventional M1 macrophages or IL-4 to transform into M2 macrophages, then Carfilzomib was added into IL-4 induced cells to further induce the formation of Ci-M1. Il-6 and Inos were checked by qRT-PCR. (b) BMDMs/Raw264.7 cells were induced with IL-4 to transform into M2 macrophages, then treated by CQ or Carfilzomib. Il-6 and Inos were checked by qRT-PCR.

3) Figure 2C: The gating strategy is unclear. How was the CD86+ and CD206+ population defined? Was it based on isotype-matched antibody staining or fluorescence minus one (FMO) control? This point is also applicable for other flow cytometric plots shown in the manuscript (e.g., Figure S2E and S2L).

Reply: Thanks very much for reminding us this important point. We defined the $CD86^+$, $CD206^+$, $MHC\text{-}I^+$ and $MHC\text{-}II^+$ population according to isotype-matched antibody staining control. We have added this important point in page 39, line

996-998 and page 51-52, line 1282-1283.

4) Figure 2F and 2H: Both assays showed that carfilzomib could enhance macrophage phagocytotic ability, but the phagocytosis efficiencies exhibit a ten-fold difference. This should be clarified.

Reply: We thank Reviewer for pointing this out to us. Figure 2F showed that the number of L1210-GFP cells were phagocytosed per 100 macrophages. In some cases, one macrophage may phagocytose multiple L1210 cells. Figure 2H indicated that the percentage of phagocytosing macrophage in total macrophage. We are using these different methods for describing phagocytosing efficiency induced by Carfilzomib.

5) Figure 2L, y axis: Is it CD8+ % or CD4+ %? Figure 3D: Extra asterisks are shown on IL-6.

Reply: Reviewer is correct. The Y axis is $CD4^{+}$ % in Figure 2L (new Figure 2L). Following Reviewer's suggestion, we have now removed the extra asterisks in new Figure 3D.

6) Figure 4: It is very interesting that the kinase activity of IRE1α, instead of the endoribonuclease activity and the activation of XBP1, mediated the carfilzomib induced reprograming of M2 macrophages. The authors are encouraged to discuss the impact of PIs and ER stress inducers on XBP1-deficienct cells.

Reply: Thanks for this great comment. Our results indicated that Kira6 (inhibiting IRE1 kinase activities), but not 4μ8C (inhibiting IRE1 RNase activities), inhibited *Il-1β* luciferase activities in *Il-1β* -luciferase transgenic Ci-M1 (Figure 4C & D). Stimulation of IRE1α activates both its kinase activity (to recruit TRAF2) and endoribonuclease activity (to splice XBP1). Spliced XBP1 transcribes genes involved in lowering unfolded protein load, including EIF2，AK3, Hspa5 and DNAJB9 (Genome Med. 2018 Oct 24;10(1):76). However, Kinase activity of IRE1α recruited TRAF2 to activate NF-κB to transcribe genes encoding M1 marker genes. We have

discuss it in Page 16-17，line 413-431.

Thank you for the submission of your revised manuscript to EMBO Molecular Medicine. We have now received the enclosed report from the two referees who were asked to re-assess it. As you will see the referees are now supportive, and I am pleased to inform you that we will be able to accept your manuscript pending the following amendments:

1. In the main manuscript file, please remove the blue color font.

2. Appendix
-The file of merged Appendix Tables needs to be named as "Appendix".
-Please add a table of contents to the first page of the appendix PDF file.

3. EV figures:
- As we accept only up to 5 EV figures, please move at least 2 EV figures to the Appendix pdf, using the nomenclature Appendix Figure S1, etc, and please move their legends to the Appendix file.
- Please also update the callouts in the main article, and make sure that all Appendix figures are called for.
- Please upload EV figures as individual, high-resolution figures.

4. Source data: please note that source data needs to be directly linked to specific figures.
-Source data for main figures: should be uploaded as one (zipped) file /figure, and named as "manuscriptID_SourceDataForFigure x"

-Source data for Expanded View and Appendix figures: uploaded as a single ZIP file containing all the Source Data for Expanded View and Appendix content. Within the ZIP file, the Source Data should be included in individual folders pertaining to the figure/table that the Source Data is for.

5. Every published paper now includes a 'Synopsis' to further enhance discoverability. Synopses are displayed on the journal webpage and are freely accessible to all readers. They include a short stand first (maximum of 300 characters, including space) as well as 2-5 one sentence bullet points that summarise the paper. Please write the bullet points to summarise the key NEW findings. They should be designed to be complementary to the abstract - i.e. not repeat the same text. We encourage inclusion of key acronyms and quantitative information (maximum of 30 words / bullet point). Please use the passive voice. Please attach these in a separate file or send them by email, we will incorporate them accordingly.

6. Synopsis image: the text becomes somewhat blurry when the image is adjusted to the required resolution (550 px width). This can be solved by increasing the text size. Please provide a new image (PNG format, 550 px width x 400-600 px height) with better readability.

7. The Paper Explained: EMBO Molecular Medicine articles are accompanied by a summary of the articles to emphasize the major findings in the paper and their medical implications for the non-specialist reader. Please provide a draft summary of your article highlighting
- the medical issue you are addressing,
- the results obtained and
- their clinical impact.
This may be edited to ensure that readers understand the significance and context of the research. Please refer to any of our published articles for an example.

8. For more information: There is space at the end of each article to list relevant web links for further consultation by our readers. Could you identify some relevant ones and provide such information as well? Some examples are patient associations, relevant databases, OMIM/proteins/genes links, author's websites, etc...

9. As part of the EMBO Publications transparent editorial process initiative (see our Editorial at http://embomolmed.embopress.org/content/2/9/329), EMBO Molecular Medicine will publish online a Review Process File (RPF) to accompany accepted manuscripts.
a. In the event of acceptance, this file will be published in conjunction with your paper and will include the anonymous referee reports, your point-by-point response and all pertinent correspondence relating to the manuscript. Let us know if you do NOT agree with this.
b. Please note that the Authors checklist will be published at the end of the RPF.

I look forward to seeing a revised version of your manuscript as soon as possible.

Kind regards
Jingyi

Jingyi Hou
Editor
EMBO Molecular Medicine

*** Instructions to submit your revised manuscript ***

*** PLEASE NOTE *** As part of the EMBO Publications transparent editorial process initiative (see our Editorial at https://www.embopress.org/doi/pdf/10.1002/emmm.201000094), EMBO Molecular Medicine will publish online a Review Process File to accompany accepted manuscripts.

In the event of acceptance, this file will be published in conjunction with your paper and will include the anonymous referee reports, your point-by-point response and all pertinent correspondence relating to the manuscript. If you do NOT want this file to be published, please inform the editorial office at contact@embomolmed.org.

To submit your manuscript, please follow this link:

https://embomolmed.msubmit.net/cgi-bin/main.plex

When submitting your revised manuscript, please include:

1) a .docx formatted version of the manuscript text (including Figure legends and tables)

2) Separate figure files*

3) supplemental information as Expanded View and/or Appendix. Please carefully check the authors guidelines for formatting Expanded view and Appendix figures and tables at https://www.embopress.org/page/journal/17574684/authorguide#expandedview

4) a letter INCLUDING the reviewer's reports and your detailed responses to their comments (as Word file).

5) The paper explained: EMBO Molecular Medicine articles are accompanied by a summary of the articles to emphasize the major findings in the paper and their medical implications for the non-specialist reader. Please provide a draft summary of your article highlighting
- the medical issue you are addressing,
- the results obtained and
- their clinical impact.
This may be edited to ensure that readers understand the significance and context of the research.
Please refer to any of our published articles for an example.

6) For more information: There is space at the end of each article to list relevant web links for further consultation by our readers. Could you identify some relevant ones and provide such information as well? Some examples are patient associations, relevant databases, OMIM/proteins/genes links, author's websites, etc...

7) Author contributions: the contribution of every author must be detailed in a separate section.

8) EMBO Molecular Medicine now requires a complete author checklist (https://www.embopress.org/page/journal/17574684/authorguide) to be submitted with all revised manuscripts. Please use the checklist as guideline for the sort of information we need WITHIN the manuscript. The checklist should only be filled with page numbers were the information can be found. This is particularly important for animal reporting, antibody dilutions (missing) and exact values and n that should be indicted instead of a range.

9) Every published paper now includes a 'Synopsis' to further enhance discoverability. Synopses are displayed on the journal webpage and are freely accessible to all readers. They include a short stand first (maximum of 300 characters, including space) as well as 2-5 one sentence bullet points that summarise the paper. Please write the bullet points to summarise the key NEW findings. They should be designed to be complementary to the abstract - i.e. not repeat the same text. We encourage inclusion of key acronyms and quantitative information (maximum of 30 words / bullet point). Please use the passive voice. Please attach these in a separate file or send them by email, we will incorporate them accordingly.

You are also welcome to suggest a striking image or visual abstract to illustrate your article. If you do please provide a jpeg file 550 px-wide x 400-px high.

10) A Conflict of Interest statement should be provided in the main text

11) Please note that we now mandate that all corresponding authors list an ORCID digital identifier. This takes <90 seconds to complete. We encourage all authors to supply an ORCID identifier, which will be linked to their name for unambiguous name identification.

Currently, our records indicate that the ORCID for your account is 0000-0001-7300-6604.

Please click the link below to modify this ORCID:
Link Not Available

12) The system will prompt you to fill in your funding and payment information. This will allow Wiley to send you a quote for the article processing charge (APC) in case of acceptance. This quote takes into account any reduction or fee waivers that you may be eligible for. Authors do not need to pay any fees before their manuscript is accepted and transferred to our publisher.

*Additional important information regarding Figures

Each figure should be given in a separate file and should have the following resolution:
Graphs 800-1,200 DPI
Photos 400-800 DPI
Colour (only CMYK) 300-400 DPI"

Figures are not edited by the production team. All lettering should be the same size and style; figure panels should be indicated by capital letters (A, B, C etc). Gridlines are not allowed except for log plots. Figures should be numbered in the order of their appearance in the text with Arabic numerals. Each Figure must have a separate legend and a caption is needed for each panel.

*Additional important information regarding figures and illustrations can be found at https://bit.ly/EMBOPressFigurePreparationGuideline. See also figure legend preparation guidelines: https://www.embopress.org/page/journal/17574684/authorguide#figureformat

The system will prompt you to fill in your funding and payment information. This will allow Wiley to send you a quote for the article processing charge (APC) in case of acceptance. This quote takes into account any reduction or fee waivers that you may be eligible for. Authors do not need to pay any fees before their manuscript is accepted and transferred to our publisher.

***** Reviewer's comments *****

Referee #1 (Comments on Novelty/Model System for Author):

The topic is improtant and closely related to the translational research.

Referee #1 (Remarks for Author):

The authors have addressed my concerns and the revised version is improved. I have no further questions.

Referee #3 (Remarks for Author):

The authors have addressed all the concerns raised by the Reviewers and have improved the manuscript accordingly.

The authors performed the requested editorial changes.

5th Nov 2021

Dear Dr. Chen,

Please find enclosed the final reports on your manuscript. We are pleased to inform you that your manuscript is accepted for publication and is now being sent to our publisher to be included in the next available issue of EMBO Molecular Medicine.

We would like to remind you that as part of the EMBO Publications transparent editorial process initiative, EMBO Molecular Medicine will publish a Review Process File online to accompany accepted manuscripts. If you do NOT want the file to be published or would like to exclude figures, please immediately inform the editorial office via e-mail.

Please read below for additional IMPORTANT information regarding your article, its publication and the production process.

Congratulations on your interesting work,

Jingyi Hou

Jingyi Hou
Editor
EMBO Molecular Medicine

Follow us on Twitter @EmboMolMed
Sign up for eTOCs at embopress.org/alertsfeeds

EMBO PRESS

YOU MUST COMPLETE ALL CELLS WITH A PINK BACKGROUND ↓

PLEASE NOTE THAT THIS CHECKLIST WILL BE PUBLISHED ALONGSIDE YOUR PAPER

Corresponding Author Name: Liang Chen, Qian Zhou
Journal Submitted to: EMBO Molecular Medicine
Manuscript Number: EMM-2021-14502

Reporting Checklist For Life Sciences Articles (Rev. June 2017)

This checklist is used to ensure good reporting standards and to improve the reproducibility of published results. These guidelines are consistent with the Principles and Guidelines for Reporting Preclinical Research issued by the NIH in 2014. Please follow the journal's authorship guidelines in preparing your manuscript.

A- Figures

1. Data

The data shown in figures should satisfy the following conditions:

- the data were obtained and processed according to the field's best practice and are presented to reflect the results of the experiments in an accurate and unbiased manner.
- figure panels include only data points, measurements or observations that can be compared to each other in a scientifically meaningful way.
- graphs include clearly labeled error bars for independent experiments and sample sizes. Unless justified, error bars should not be shown for technical replicates.
- if n< 5, the individual data points from each experiment should be plotted and any statistical test employed should be justified
- Source Data should be included to report the data underlying graphs. Please follow the guidelines set out in the author ship guidelines on Data Presentation.

2. Captions

Each figure caption should contain the following information, for each panel where they are relevant:

- a specification of the experimental system investigated (eg cell line, species name).
- the assay(s) and method(s) used to carry out the reported observations and measurements
- an explicit mention of the biological and chemical entity(ies) that are being measured.
- an explicit mention of the biological and chemical entity(ies) that are altered/varied/perturbed in a controlled manner.

- the exact sample size (n) for each experimental group/condition, given as a number, not a range;
- a description of the sample collection allowing the reader to understand whether the samples represent technical or biological replicates (including how many animals, litters, cultures, etc.).
- a statement of how many times the experiment shown was independently replicated in the laboratory.
- definitions of statistical methods and measures:
  - common tests, such as t-test (please specify whether paired vs. unpaired), simple $\chi 2$ tests, Wilcoxon and Mann-Whitney tests, can be unambiguously identified by name only, but more complex techniques should be described in the methods section;
  - are tests one-sided or two-sided?
  - are there adjustments for multiple comparisons?
  - exact statistical test results, e.g., P values = x but not P values < x;
  - definition of 'center values' as median or average;
  - definition of error bars as s.d. or s.e.m.

Any descriptions too long for the figure legend should be included in the methods section and/or with the source data.

USEFUL LINKS FOR COMPLETING THIS FORM

http://www.antibodypedia.com
http://1degreebio.org
http://www.equator-network.org/reporting-guidelines/improving-bioscience-research-repor
http://grants.nih.gov/grants/olaw/olaw.htm
http://www.mrc.ac.uk/Ourresearch/Ethicsresearchguidance/Useofanimals/index.htm
http://ClinicalTrials.gov
http://www.consort-statement.org
http://www.consort-statement.org/checklists/view/32-consort/66-title
http://www.equator-network.org/reporting-guidelines/reporting-recommendations-for-tumo
http://datadryad.org
http://figshare.com
http://www.ncbi.nlm.nih.gov/gap
http://www.ebi.ac.uk/ega
http://biomodels.net/
http://biomodels.net/miriam/
http://jjj.biochem.sun.ac.za
http://oba.od.nih.gov/biosecurity/biosecurity_documents.html
http://www.selectagents.gov/

In the pink boxes below, please ensure that the answers to the following questions are reported in the manuscript itself. Every question should be answered. If the question is not relevant to your research, please write NA (non applicable). We encourage you to include a specific subsection in the methods section for statistics, reagents, animal models and human subjects.

B- Statistics and general methods

Please fill out these boxes ↓ (Do not worry if you cannot see all your text once you press return)

1.a. How was the sample size chosen to ensure adequate power to detect a pre-specified effect size?	Samples size was set such that statistical significance can be reached.
1.b. For animal studies, include a statement about sample size estimate even if no statistical methods were used.	Statistical analysis was done on all experiment that we used mice. we have indicated the mouse numbers in Materials and Methods and in Figure legends.
2. Describe inclusion/exclusion criteria if samples or animals were excluded from the analysis. Were the criteria pre-established?	In experiment when we used Carfilzomib or other treatments (as indicatated in the manuscript) to treat transgenic mice, computed tomography was conducted to confirm lung cancer in mice. Mice bearing no noticable lung cancers before treatment were excluded from analysis.
3. Were any steps taken to minimize the effects of subjective bias when allocating animals/samples to treatment (e.g. randomization procedure)? If yes, please describe.	In our treatment studies, we chose a cohort of mice bearing similar burden of lung cancer. These mice were then randomized to different treatments.
For animal studies, include a statement about randomization even if no randomization was used.	The mice were randomized for treatment. We have indicated this in main text.
4.a. Were any steps taken to minimize the effects of subjective bias during group allocation or/and when assessing results (e.g. blinding of the investigator)? If yes please describe.	ImageJ software was used to analyze the CT images for determining tumor burdens.
4.b. For animal studies, include a statement about blinding even if no blinding was done	Statement was included in material and methods.
5. For every figure, are statistical tests justified as appropriate?	Yes, statistical tests were justified as appropriate.
Do the data meet the assumptions of the tests (e.g., normal distribution)? Describe any methods used to assess it.	Not applicable
Is there an estimate of variation within each group of data?	No, there was no estimate of variation within each group of data.

Is the variance similar between the groups that are being statistically compared?	All data in our experiments were statistically analyzed.

C- Reagents

6. To show that antibodies were profiled for use in the system under study (assay and species), provide a citation, catalog number and/or clone number, supplementary information or reference to an antibody validation profile. e.g., Antibodypedia (see link list at top right), 1DegreeBio (see link list at top right).	Antibodies used in our study have catalog number and source company informations in Materials and Methods and Appendix Table S1. Dilution information was provided in figure legends or Materials and Methods and Appendix Table S1.
7. Identify the source of cell lines and report if they were recently authenticated (e.g., by STR profiling) and tested for mycoplasma contamination.	We didn't used any of the cell lines listed in Database of Cross-Contaminated or Misidentified Cell Lines (Amanda Capes-Davis and R. Ian Freshney. (https://www.nature.com/articles/492186a).

* for all hyperlinks, please see the table at the top right of the document

D- Animal Models

8. Report species, strain, gender, age of animals and genetic modification status where applicable. Please detail housing and husbandry conditions and the source of animals.	C57BL/6J mice were purchased from Beijing Vital River Laboratory Animal Technology Co. Ltd.. Mice used in our study is housed in SPF animal facility. They are of C57BL/6 background. These details were stated in Materials and Methods.
9. For experiments involving live vertebrates, include a statement of compliance with ethical regulations and identify the committee(s) approving the experiments.	Yes, this information is stated in Materials and Methods.All animals were housed in specific pathogen-free conditions and breeding and all animal procedures were conducted in strict accordance with guidelines for the care and use of laboratory approved by the Institute of Laboratory Animal Science, Jinan University.
10. We recommend consulting the ARRIVE guidelines (see link list at top right) (PLoS Biol. 8(6), e1000412, 2010) to ensure that other relevant aspects of animal studies are adequately reported. See author guidelines, under 'Reporting Guidelines'. See also: NIH (see link list at top right) and MRC (see link list at top right) recommendations. Please confirm compliance.	Yes, this study is carried out with strict accordance with these international guidelines.

E- Human Subjects

11. Identify the committee(s) approving the study protocol.	Ethnic committee of Jinan University
12. Include a statement confirming that informed consent was obtained from all subjects and that the experiments conformed to the principles set out in the WMA Declaration of Helsinki and the Department of Health and Human Services Belmont Report.	Informed consent was obtained from all subjects. The experiments conformed to the principles set out in the WMA Declaration of Helsinki and the Department of Health and Human Services Belmont Report.
13. For publication of patient photos, include a statement confirming that consent to publish was obtained.	Not applicable
14. Report any restrictions on the availability (and/or on the use) of human data or samples.	Not applicable
15. Report the clinical trial registration number (at ClinicalTrials.gov or equivalent), where applicable.	Not applicable
16. For phase II and III randomized controlled trials, please refer to the CONSORT flow diagram (see link list at top right) and submit the CONSORT checklist (see link list at top right) with your submission. See author guidelines, under 'Reporting Guidelines'. Please confirm you have submitted this list.	Not applicable
17. For tumor marker prognostic studies, we recommend that you follow the REMARK reporting guidelines (see link list at top right). See author guidelines, under 'Reporting Guidelines'. Please confirm you have followed these guidelines.	Not applicable

F- Data Accessibility

18: Provide a "Data Availability" section at the end of the Materials & Methods, listing the accession codes for data generated in this study and deposited in a public database (e.g. RNA-Seq data: Gene Expression Omnibus GSE39462, Proteomics data: PRIDE PXD000208 etc.) Please refer to our author guidelines for 'Data Deposition'.<br><br>Data deposition in a public repository is mandatory for:<br>a. Protein, DNA and RNA sequences<br>b. Macromolecular structures<br>c. Crystallographic data for small molecules<br>d. Functional genomics data<br>e. Proteomics and molecular interactions	Not applicable
19. Deposition is strongly recommended for any datasets that are central and integral to the study; please consider the journal's data policy. If no structured public repository exists for a given data type, we encourage the provision of datasets in the manuscript as a Supplementary Document (see author guidelines under 'Expanded View' or in unstructured repositories such as Dryad (see link list at top right) or Figshare (see link list at top right).	Western blot photos are provided as Sourse data.
20. Access to human clinical and genomic datasets should be provided with as few restrictions as possible while respecting ethical obligations to the patients and relevant medical and legal issues. If practically possible and compatible with the individual consent agreement used in the study, such data should be deposited in one of the major public access-controlled repositories such as dbGAP (see link list at top right) or EGA (see link list at top right).	Not applicable
21. Computational models that are central and integral to a study should be shared without restrictions and provided in a machine-readable form. The relevant accession numbers or links should be provided. When possible, standardized format (SBML, CellML) should be used instead of scripts (e.g. MATLAB). Authors are strongly encouraged to follow the MIRIAM guidelines (see link list at top right) and deposit their model in a public database such as Biomodels (see link list at top right) or JWS Online (see link list at top right). If computer source code is provided with the paper, it should be deposited in a public repository or included in supplementary information.	Not applicable

G- Dual use research of concern

22. Could your study fall under dual use research restrictions? Please check biosecurity documents (see link list at top right) and list of select agents and toxins (APHIS/CDC) (see link list at top right). According to our biosecurity guidelines, provide a statement only if it could.	Not applicable
